# Entropy of Charge Inversion in DNA including One-Loop Fluctuations

**DOI:** 10.3390/e25101373

**Published:** 2023-09-24

**Authors:** Matthew D. Sievert, Marilyn F. Bishop, Tom McMullen

**Affiliations:** 1 Department of Physics, New Mexico State University, Las Cruces, NM 88003-8001, USA; 2 Department of Physics, Virginia Commonwealth University, Richmond, VA 23284-2000, USA; tmcmulle@vcu.edu

**Keywords:** entropy, DNA, biomolecules, polyelectrolyte, fluctuations, charge inversion, correlations, field theory, one-loop order

## Abstract

The entropy and charge distributions have been calculated for a simple model of polyelectrolytes attached to the surface of DNA using a field-theoretic method that includes fluctuations to the lowest one-loop order beyond mean-field theory. Experiments have revealed correlation-driven behavior of DNA in charged solutions, including charge inversion and condensation. In our model, the condensed polyelectrolytes are taken to be doubly charged dimers of length comparable to the distance between sites along the phosphate chains. Within this lattice gas model, each adsorption site is assumed to have either a vacancy or a positively charged dimer attached with the dimer oriented either parallel or perpendicular to the double-helix DNA chain. We find that the inclusion of the fluctuation terms decreases the entropy by ∼50% in the weak-binding regime. There, the bound dimer concentration is low because the dimers are repelled from the DNA molecule, which competes with the chemical potential driving them from the solution to the DNA surface. Surprisingly, this decrease in entropy due to correlations is so significant that it overcompensates for the entropy increase at the mean-field level, so that the total entropy is even lower than in the absence of interactions between lattice sites. As a bonus, we present a transparent exposition of the methods used that could be useful to students and others wishing to use this formulation to extend this calculation to more realistic models.

## 1. Introduction

Entropy is often described as a measure of disorder. Its importance is that the free energy of a system involves a trade-off between the entropy and the energy. The simplest example of this is the Helmholtz free energy F=E−TS, which applies to a system with a fixed number of particles and a fixed volume at constant temperature. Because of the complexity of biological systems, there are many instances in which the TS term of the free energy either dominates the energy term, or at least plays an important role in the free energy minimization that determines equilibrium and drives the dynamics of the system.

As a consequence, the determination of the optimal structure of many biologically important systems requires minimization of the free energy, and hence this competition between the internal energy and entropy. Often in biological systems the changes in entropy are greater than the changes in internal energy. For instance, in the hydrophobic effect, an unfolded protein lowers the entropy by ordering the water molecules, and so the protein prefers to be in the folded state [1,2]. In the chloroplast stroma, it has been shown that there is an entropy-driven attraction that determines the chloroplast ultrastructure through spontaneous Mg${}^{2+}$-induced stacking of membranes [3].

In sickle hemoglobin, the aggregation of monomers into polymers [4] is also entropy driven, with the internal energy and entropy in a delicate balance. In fact, Cao and Ferrone [5] measured the chemical potential $\mu_{PC}$, which is the “glue” that holds the polymer together, and found that the entropic contribution to this chemical potential is around −11 kcal/mol, which is more than 100% of the chemical potential itself. Another example is F-actin, whose fibrils stack in cross-linked rafts when positive alkaline earth ions are in the solution [6]. In F-actin, counter-ions form one-dimensional charge density waves that have a periodicity equal to twice the actin monomer spacing, coupling to twist distortions of the oppositely charged actin filaments [7]. These phenomena and others owe their existence to Coulomb interactions between the constituent parts, and the geometry of the underlying structure can also play a vital role.

Thermodynamically, the distribution of charge in the solution is governed by a competition between energy and entropy. The bound state in which the ions are condensed on the surface of the macro-ion/polyelectrolyte is energetically favored, and the continuum state, in which the ions are free to drift in any direction, is entropically favored. The balance between these two factors in minimizing the free energy has been shown to vary significantly based on the geometry of the macro-ion [8]. This interplay between electrostatics and geometry is particularly important for polyelectrolytes in solution, and they have been shown to produce a wide array of intriguing phenomena in many different systems. One example is DNA, which in the presence of a physiological salt solution (a 0.1 molar solution of NaCl) is usually negatively charged, with one double-helix DNA molecule strongly repelling another. However, if DNA is in a dilute salt solution in which a positively charged polyelectrolyte, such as spermine or sperimidine, has been added to the solution, it has been shown to roll up into a tightly packed torus [8,9,10,11]. In fact, DNA is usually in a very compact state in cells and viruses.

Historically, the most common description of charged solutions has been the Poisson–Boltzmann theory, but continued research has indicated that charged species in solution can have far more complex and counterintuitive effects than simple charge screening [8]. In 1969, Manning proposed [12,13] that a portion of the ambient ions condense onto (i.e., attach to) the surface of a charged macro-ion, partially neutralizing the bare charge. This occurs up to the point at which the energy required to condense another ion equals the available thermal energy $k_{B}T$ at temperature *T*, where $k_{B}$ is the Boltzmann constant. The proposed effect, later termed “Manning condensation”, marked a significant departure from linearized Poisson–Boltzmann theory that predicted only exponential screening. In Manning’s treatment of polyelectrolytes, which are long chains of charged subunits, the ion condensation was addressed [8] separately from the ions that remain in solution, which were treated using linearized Poisson–Boltzmann theory [12,13].

Further refinements in the treatment of these ambient-ion solutions have been motivated in part by surprising effects observed in DNA that cannot be accounted for using Poisson–Boltzmann theory. By using multivalent cations, modifying the salts in the solutions, and even using alcohol solvents, it is possible to cause the DNA to undergo a radical structural transition into a variety of new geometries, including rod-like bundles and toroids [14]. The toroidal structures in particular have received considerable attention in the biological community, because such toroidal packing motifs are employed by spermidine and other molecules to contain their own DNA in small volumes [15]. A variety of techniques, including cryo-transmission electron microscopy [15] and UV spectroscopy [16], have enabled direct observation of the formation of these toroidal condensates, and similar studies on other biological polyelectrolytes like F-actin [6] indicate that condensation is a general phenomenon. If the electrical interactions between the polyelectrolyte, such as DNA, and the ambient-ion solution are purely those predicted by the Poisson–Boltzmann description, then two like-charged polyelectrolytes always repel one another, and such condensates will not form. For these condensates to be stable, the net electrical force between like-charged polyelectrolytes must be attractive, which is incompatible with the Poisson–Boltzmann theory of ions in solution.

Many of the approaches to understanding the role of polyelectrolytes in biological systems have adopted models of continuum electrostatics, and some of these have focused on solving the Poisson–Boltzmann equation for a cylindrical model of DNA in the presence of multivalent ions [8,17]. However, it has been shown that including charge correlations is essential to understanding these systems [18,19]. Recognizing the need for including fluctuations beyond the mean field, Ha and Liu used a field-theoretic approach that produced a systematic expansion taking these effects into account [20,21,22,23]. They employed a simplified model of a DNA particle as a charged cylindrical rod composed of cylindrical charged segments and considered these rod-like particles in the presence of polyvalent counterions. Their first model consisted of two rod-like DNA molecules [20], and they found attraction between the rods. When Podgornik and Parsegian suggested that the fluctuation forces between such rod-like polyelectrolytes might not be pairwise additive [24], Ha and Liu extended their calculation to include bundles of rod-like particles and included a one-dimensional form factor depending on the ionic size in order to incorporate short-ranged correlations along the rod length approximately [21,22]. They found that the breakdown of pairwise additivity can lead to qualitatively new behavior, although they still found that the rods attracted one another under some conditions. Shklovskii then pointed out that the previous results seemed to be independent of radius, and that, in a highly correlated system of negative macro-ion charged cylindrical rods in the presence of positive counterion charges, those positive charges can form a Wigner crystal, which will be three-dimensional and closely packed for small rod radii and form two-dimensional Wigner crystals on the surfaces of rods for large radii [25]. Ha and Liu saw merit in the arguments for the large radius limit but disagreed with the the two-dimensional crystal lattice limit, which they said was due to Shklovskii incorrectly assuming pairwise additive interactions between various surfaces, which would make that limit invalid [22].

To show how the radius of a DNA chain could affect multivalent-counterion-induced attraction between negatively charged DNA chains, Ha and Liu used a modified model of DNA [23]. They assumed, as before, that a DNA rod is a stack of short cylinders that have a finite radius and length, but to treat two-dimensional charge fluctuations on the surface of the rod, they divided the counterions into two classes, free and condensed, with the condensed counterions modifying the local charge at the surface of the cylinder, giving rise to charge fluctuations on the two-dimensional surface of each cylinder. With this model, they found that the competition between attractive and repulsive interactions tended to balance one another, resulting in no attraction at all for thick rods. They also found that for a valence of the polyvalent cations greater than around three, the spacing of the chains in a bundle and the size of bundles appears nearly independent of the nature of the bundling agent. This is because the increased valence leads to a stronger screening as well as a stronger attraction.

**Figure 1:**
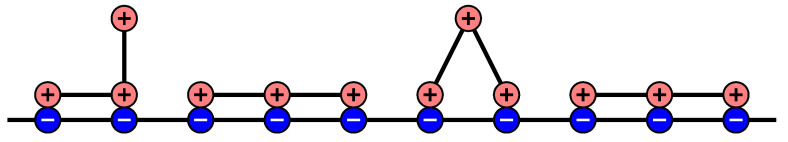
Charge fractionalization occurs when only a fraction of the positive charges of the polyelectrolyte attach to the negatively charged surface. The charges that are not attached then cause the surface to become positively charged. This shows a freely jointed polyelectrolyte chain of charge 3e; adapted from Figure 1b of Nguyen and Shklovskii [26].

Recognizing that geometry was playing a crucial role in the attraction, Nguyen and Shklovskii [26,27] proposed a simple theory of charge inversion that considers the structure of the polyelectrolyte together with its electrostatic interaction with the substrate. The idea was that there is fractionalization of charge in which a polyelectrolyte can either neutralize the charge or reverse it depending on how it attaches. In that model, a single double-helix strand of DNA is represented by a rigid cylinder with two one-dimensional lattices of negative charges −e in helices around the surface to represent DNA’s double helix of negatively charged phosphates. In order to model the polyelectrolyte solution in which the DNA is immersed, they take the positively charged species to be freely jointed chains. These adsorbing species have multiple charges that may partially attach to the surface, with excess charge protruding into the solution, as shown in Figure 1. They assumed that all the negative charges on the DNA are neutralized by the polyelectrolytes, so that there will be no vacancies, and because there is excess charge dangling into the solution, the charge on the surface can be not only neutralized but reversed. They also assume that the only role of the background salt solution is to provide screening for the interaction between the polyelectrolyte chains. Since they assumed no vacancies on the one-dimensional chains, they could easily count the possible configurations. In calculating the electrical potential, they took the negative charges of the DNA to be spread uniformly on the surface of the cylindrical DNA surface and used simple electrostatic arguments with screened potentials to calculate the energies involved. For polyelectrolytes in a 0.1M NaCl solution with DNA, Nguyen and Shklovskii [26] give in their Equation (6) an estimate of the net charge inversion, the charge density on DNA divided by its bare charge density. The largest value of that estimate is about 0.07.

Since the simple calculation of Nguyen and Shklovskii [26] did not include the discrete nature of the phosphate sites in calculating the electrostatics and used a simple version of the entropy, Bishop and McMullen [28] decided to use a more rigorous formalism to calculate the charge reversal in DNA. Starting with the simplifying assumption that the polyelectrolytes could be considered as dimers (two units in length) on DNA, Bishop and McMullen allowed for vacancies and used a model of the discrete location of phosphates on the DNA surface, with interactions between charges located on the various sites. In that model, doubly charged polyelectrolyte molecules attached to the DNA either parallel or perpendicular to the DNA surface, and the model confirmed that under the right conditions, the charge on the surface could be inverted. The use of the lattice model allowed them to accommodate vacancies, which is not possible if the charge is treated in a continuum manner. Inspired by the work of Ha and Liu [20,21,22,23], they used a field-theoretic approach that employs a loop expansion, where the mean field contains polyelectrolyte ions adsorbed on the surface of the DNA, similar to their approach. While the general formalism for the one-loop expansion was presented in that paper, the numerical calculations stopped at the mean-field level, and the current work extends that to include fluctuations for the one-loop method. The emphasis here is on the entropy and the role it plays in the configuration of charged species attached to the lattice. The details of this model will be explained in detail in the next section.

The results of these theories indicate that the correlation effects in the solution are also strongly dependent on the system geometry. To understand this, consider the electric potential outside spherical, cylindrical, and planar positively charged surfaces in vacuum. For spheres, the potential decays as the inverse of the distance; for the cylinder, the potential decreases logarithmically with the distance; and for the plane, the potential decreases linearly with the distance. Gelbart et al. [8] assert that in an ambient-ion solution, the entropy of the point ions varies logarithmically with their concentration, and therefore, with the distance from the cylinder. Thus, they conclude that, in a crude comparison, for spherical geometry, the $r^{-1}$ potential is dominated by the logarithmic entropy; for planar geometry, the entropy is dominated by the linear potential; and for cylindrical geometry, both the energy and the entropy vary logarithmically [29]. In this paper, we will incorporate the impact of the ions in solution on the free energy through the chemical potential of these ions, as detailed in Appendix B.

The helical geometry of DNA, then, sits precisely balanced on the fulcrum between energy- and entropy-driven processes under physiological conditions. The geometry of the biomolecule plays an even more crucial role when the highly charged ions in solution are not point charges but have geometries of their own. Such is the case for DNA immersed in a solution of polyelectrolytes. These polyelectrolytes can be proteins or other fragments of biomolecules which are routinely found in the nucleus [30], so that, again, the central biological processes occur in precisely the most difficult regimes to model.

For charge inversion on DNA with polyelectrolytes, “physiological conditions” require incorporating the combined effects of charge correlation, thermodynamic fluctuations, crowding, and geometrical considerations all at once. As we have discussed in this introduction, there has been considerable work in addressing all of these issues. Some approaches treat only the geometry, with no interactions [31]. Others include both geometry and interactions, but use a continuum model for electrostatics that neglects discreteness effects [32]. The lattice gas dimer model is unique in its simultaneous consideration of all these effects, and the thermodynamic and geometrical idealizations it makes can be systematically improved.

While the formalism of Bishop and McMullen [28] included both a mean-field theory and corrections due to fluctuations or charge correlations, the computed results were obtained only at the mean-field level. In this paper, this simple model is extended to calculate the fluctuation corrections and the entropy of the system. The purpose is to determine the importance of the fluctuation terms for inverting the charge and to see whether these terms have a significant impact on the entropy of the system. A preliminary version of this work was in Sievert’s master’s thesis [33]. In Section 2 of this paper, we outline the model of the charged binding sites on DNA and present the computation of the entropy due only to the hard-core repulsion that prevents multiple binding on the same site. In Section 3, we explain the geometry of the double helix and show how it can be represented as a one-dimensional lattice, and in Section 4, derive the form of the potential and determine the orthogonal transformation that diagonalizes it. In Section 5, we use functional integral techniques to derive the partition function, which uses a Gaussian integral identity to perform the sum over configurations exactly, at the expense of an integral over a new auxiliary field. In Section 6, we show how the grand canonical potential can be computed order by order, in which the first two terms are the mean field and the one-loop correction to the mean field. In Section 7, we examine the saddle-point equation that defines the mean field, and this is used to calculate the entropy, the number per site of all species, and the charge per site. In Section 8, we find the explicit form for the inverse Hartree-field-fluctuation propagator. In Section 9, we include the one-loop order terms in the calculations to reveal the effects of fluctuations. Finally, in Section 10, we compare the results of the various approximations. Details concerning the Gaussian integral identity and the chemical potential of dimers in solution are given in Appendices Appendix A and Appendix B.

## 2. The Noninteracting DNA Lattice Gas

**Figure 2:**
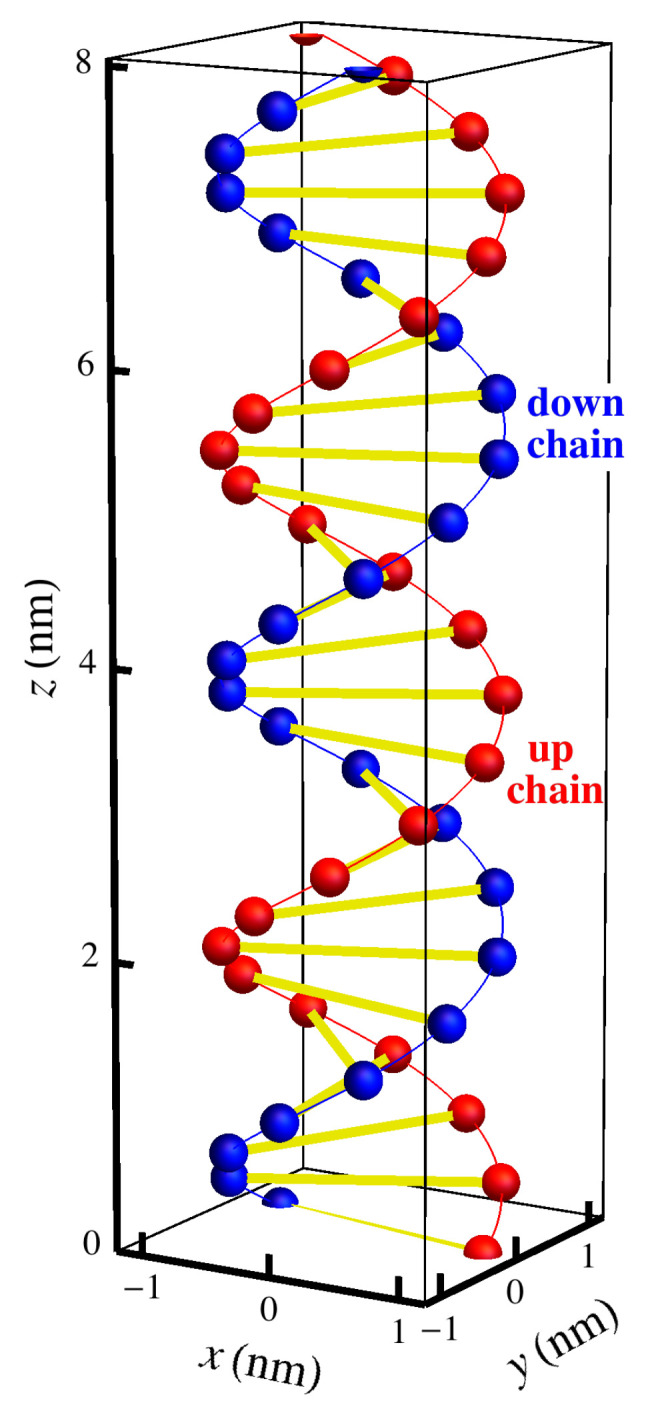
Model of DNA as two helical chains of phosphates, shown as red and blue balls, with the yellow lines representing the base pairs. These have been labeled “up” and “down” chains, corresponding to the direction of the carbon atoms in the sugar backbone.

**Figure 3:**
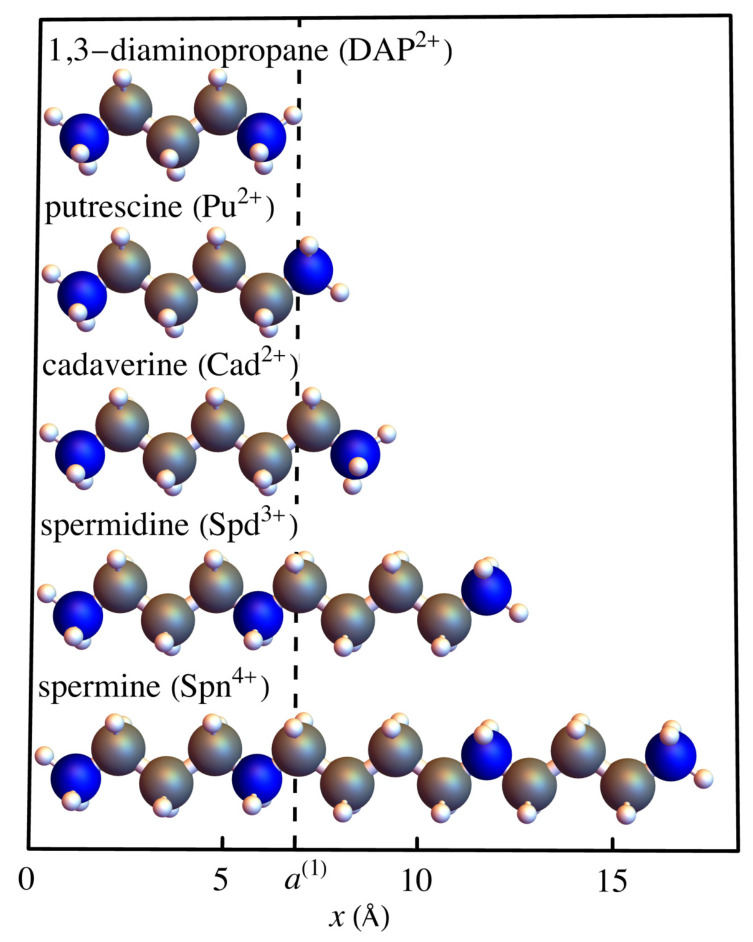
Polyamines as candidates for attachment to DNA. The diamines, including 1,3-diaminopropane (DAP${}^{2+}$), putrescine (Put${}^{2+}$), and cadaverine (Cad${}^{2+}$), have charge +2e, with sufficient length to attach to adjacent phosphates along the DNA chain, with DAP${}^{2+}$ being the best fit. These are the most likely polyelectrolytes that the dimers in our model represent. Higher-charged polyamines spermidine (Spd${}^{3+}$) and spermine (Spn${}^{4+}$), shown for comparison, are considerably longer. The lattice spacing $a^{\left(1\right)}$ between phosphates along a helix is shown as a dashed line for comparison. Here, the gray spheres correspond to carbon atoms, the blue spheres to nitrogen atoms, and the small white spheres to hydrogen atoms.

Our model system starts with a simplified version of the DNA molecule itself, as shown in Figure 2, with double-helical chains of phosphate ions connected by base pairs. The phosphate ions are represented by red balls and blue balls, with the base pairs represented by yellow lines. The two different chains are labeled “up chain” and “down chain”, which correspond to the direction of the carbon atoms in the sugar backbone. We will assume that each phosphate site is a point charge that has charge −e, where *e* is magnitude of the charge of an electron, and that there are no charges either between the phosphate sites along the helical chain or in the vicinity of the base pairs. In solution are polyelectrolyte ions of charge +2e, which we call dimers. These dimers are assumed to be the length of the spacing between two phosphate ions along a helical chain, which is $a^{\left(1\right)}=0.684$ nm =6.84 Å, which is longer than a diatomic molecule. Possible candidates for these species are the diamines, 1,3-diaminopropane (DAP${}^{2+}$), putrescine (Put${}^{2+}$), and cadaverine (Cad${}^{2+}$). An extension of this model could include more highly charged spermidine (Spd${}^{3+}$) or spermine (Spn${}^{4+}$). These polyamines are shown in Figure 3, with the lattice spacing $a^{\left(1\right)}$ shown as the dashed line for comparison. Although we will only consider the doubly charged species in our model, the model and analysis could be extended to these more highly charged species in the future.

Deng and Bloomfield have shown, using Raman difference spectra, for the systems they studied, that in the presence of spermidine or spermine these polyvalent cations bind electrostatically near the DNA phosphates [34]. Van Dam et al. agree with this conclusion and suggest that the A form of DNA is stabilized by polyamines fitting perfectly between the phosphate ions [35]. They conclude that DAP, which has charge +2e, probably has the best fit to the phosphate lattice of all the polyamines, and that longer species, like spermine and sperimidine, are longer and probably also interact with base pairs. In our model, we are assuming that there is no interaction with the base pairs.

**Figure 4:**
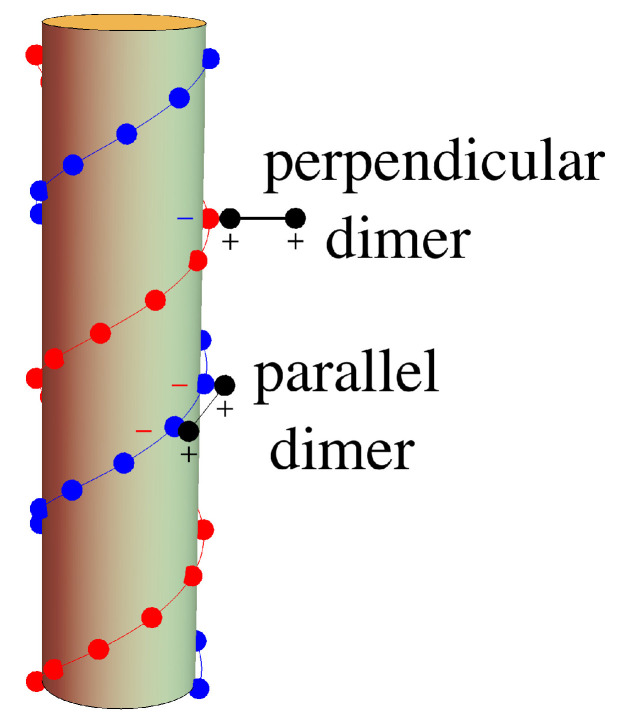
Double-helix of negatively charged phosphate chains of red and blue balls are wrapped around a cylinder. A parallel dimer attaching to one of the chains would neutralize two sites, while a perpendicular dimer would have one excess charge dangling into the solution, causing that site to have a net positive charge.

The choice of Bishop and McMullen [28] to model dimers was motivated by the wealth of studies (refs. [36,37], among others) in the literature about dimer models in other branches of physics, as well as for geometrical simplicity. We will continue to use this same model. Dimers, the shortest polyelectrolytes, have only two possible orientations when adsorbed on DNA, assuming that the length of the dimer is comparable to the helical spacing between the charged sites on DNA, as shown in Figure 4. As we stated earlier, this should not be confused with a diatomic molecule, which would not be long enough to span the space distance between two phosphate ions on one of the helical chains of DNA.

In our model, a dimer lying on the cylindrical surface of the molecule must lie parallel to the helical strands, neutralizing the charge on two adjacent sites. Otherwise, the dimer must adsorb perpendicular to the cylindrical surface, extending one end radially out from the central axis of the cylinder and inverting the charge on a single site. Charge inversion by dimers, then, is quite similar to charge inversion due to two species of point ions: one monovalent species representing parallel-adsorbed dimers and one divalent species representing perpendicular-adsorbed dimers. In previous work, Bishop and McMullen [28] modeled the adsorption of dimers in a lattice gas model as a two-component solution of point ions, allowing the possibility of vacancies, and used field-theoretic methods to describe the thermodynamics of the system. They carried their calculations to a mean-field level of approximation of the inverted charge on DNA, but did not calculate the entropy. Their work confirmed the possibility of charge inversion within this model.

Those computations yielded the charge per site on the DNA helix as a function of the chemical potential, or equivalently of the concentration of the polyelectrolyte in solution. While the Nguyen–Shklovskii [26] calculation assumed complete filling of the lattice, the Bishop–McMullen approach allowed for vacancies, represented as negatively charged sites, in addition to the neutral or positively charged sites arising from dimer adsorption. However, the lattice gas model, which assumes all sites are equivalent, does not take into account that the parallel dimer occupies two sites. In this model, the binding energy of the parallel dimer is taken to be $\varepsilon^{\left(\|\right)}$ and for the perpendicular dimer $\varepsilon^{\left(⊥\right)}$, and these energies can be adjusted to account for the difference in occupancy. In practice, the occupation of two sites is mimicked by making the binding energy of the parallel dimer twice as large as that of the perpendicular dimer, and we will use the same approach here in the numerical calculations. The assignment of binding energies to these species is a simple way of including the complicated electrostatics that allows the polyelectrolyte ions to bind to the surface of the DNA. This is analogous to the “fermion” model of Ha and Liu [22], which assumes that each site can either be empty or occupied by one counterion. Here, as in the earlier Bishop and McMullen work, we have three species, parallel dimers, perpendicular dimers, and vacancies.

This approach allows us to describe adsorption of any of the species independently for each site. Any configuration of the DNA–dimer complex in this model can then be described by identifying the type of dimer (parallel, perpendicular, or none) adsorbed on each site on the DNA molecule. Such a model also resembles the lattice gas model of condensed matter physics [38], which treats the ways of distributing particles of different types onto a regular lattice of sites. In this section, we assume that the three different species on the lattice do not interact with one another, and in this case, the structure of the lattice does not matter. We call this the noninteracting model. This does not actually mean that there are no interactions. The interaction of the polyelectrolytes with the DNA can actually be quite strong, depending on the values of $\varepsilon^{\left(\|\right)}$ and $\varepsilon^{\left(⊥\right)}$, and their attachment to the lattice is analogous to what Ha and Liu call condensed counterions [22]. Also, we will assume that there is only one species per site, which corresponds to a hard-core repulsion between sites.

In developing the formalism, it is convenient to consider a vacancy as a third species γ of particle, because then we can impose the constraint that each site is singly occupied, either by a parallel dimer (γ=‖), a perpendicular dimer (γ=⊥), or a vacancy (γ=v). For each our three species of “particle” that can reside on our lattice sites, we define a relative charge $q_{\gamma }$ in units of the magnitude *e* of the charge of an electron. These relative charges are then $q_{v}=-1$ for vacancies, $q_{\|}=0$ for parallel dimers, and $q_{⊥}=+1$ for perpendicular dimers. We will assume that the binding energy $\varepsilon_{\gamma }$ of species γ to the lattice depends only on the type of species and not the location. We will be specifying each lattice point by its location *ℓ* along helix *b*, with the pair (ℓ,b) specifying that lattice position. Then, the quantity $n_{\ell ,b}^{\gamma }$ will be the number of particles of species γ on the lattice site at *ℓ* on chain *b*. For each chain, *ℓ* extends from −N to N, such that the total number of sites is $N_{\mathrm{sites}}=2\left(2N+1\right)$, and we employ periodic boundary conditions. There will also be free polyelectrolyte ions in solution, and their influence on the stability of the adsorbed dimers will be through their chemical potential.

We begin by considering the Hamiltonian $H_{\mathrm{NI}}$ for this noninteracting system (that is, with no interactions between different sites aside from the hard-core repulsion that blocks double occupancy), which is

(1) $$H_{\mathrm{NI}}=\sum_{\ell ,b}\sum_{\gamma }\varepsilon_{\gamma }n_{\ell ,b}^{\left(\gamma \right)}.$$

Because we have a system that exchanges particles with its surroundings, specifically the ions in the solution surrounding the DNA, which attach to the surface, we work in the grand canonical ensemble. If the system contains $N_{\gamma }$ particles of type γ in equilibrium with its surroundings, with an average internal energy *E*, the grand canonical potential $\Omega_{G}$, which is a function of the temperature *T*, volume *V*, and chemical potentials $\mu_{\gamma }$ for each species γ, is written as [38,39],

(2) $$\Omega_{G}\left(T,V,\left\{\mu_{\gamma }\right\}\right)=E-TS-\sum_{\gamma }\mu_{\gamma }N_{\gamma }\;,$$

where the number of particles of type γ is given by

(3) $$N_{\gamma }=\sum_{\ell ,b}n_{\ell ,b}^{\left(\gamma \right)}\;.$$

Then, the entropy can be written in terms of this potential as

(4) $$S=-{\left(\frac{\partial \Omega_{G}}{\partial T}\right)}_{V,\{\mu_{\gamma }\}}=k_{B}\beta^{2}{\left(\frac{\partial \Omega_{G}}{\partial \beta }\right)}_{V,\{\mu_{\gamma }\}}\;,$$

where $\beta =\frac{1}{k_{B}T}$ and $k_{B}$ is Boltzmann’s constant. In this partial derivative, the volume and all the chemical potentials $\mu_{\gamma }$ of all species γ are held constant. It is convenient to define the grand canonical partition function $Z_{G}$ in terms of the grand canonical potential $\Omega_{G}$ as

(5) $$Z_{G}=\sum_{\mathrm{configurations}}e^{-\beta (E-\sum_{\gamma }\mu_{\gamma }N_{\gamma })}=e^{-\beta \Omega_{G}}\;,$$

where the sum over configurations includes all the accessible states of the system. The grand canonical potential can alternatively be written in terms of the partition function as

(6) $$\Omega_{G}=-\frac{\ln Z_{G}}{\beta }\;,$$

and this allows us to write an expression for the entropy in terms of $Z_{G}$ as

(7) $$S=k_{B}\ln Z_{G}-\frac{k_{B}\beta }{Z_{G}}{\left(\frac{\partial Z_{G}}{\partial \beta }\right)}_{V,\{\mu_{\gamma }\}}\;.$$

For the noninteracting lattice, the average internal energy is represented here by the Hamiltonian (1), $E=H_{NI}$, and the noninteracting grand canonical partition function $Z_{\mathrm{NI}}$ becomes

(8) $$Z_{\mathrm{NI}}=\sum_{\mathrm{configurations}}e^{-\beta \sum_{\ell ,b}\sum_{\gamma }\left(\varepsilon_{\gamma }-\mu_{\gamma }\right)n_{\ell ,b}^{\left(\gamma \right)}}\;,$$

where we have suppressed the *G* subscript for simplicity.

Since a vacant site does not really correspond to a particle, we recognize that energy and chemical potential of the vacancy must be related such that $\varepsilon^{\left(v\right)}-\mu_{v}=0$. In addition, because the dimers adsorbed on the surface and those in solution are in equilibrium, $\mu_{\|}=\mu_{⊥}=\mu_{\mathrm{dimer}}$ is the chemical potential of dimers in solution at the appropriate concentration. We, thus, need an estimate for $\mu_{\mathrm{dimer}}$. Approximating the dimer as a uniformly charged cylinder, this value is shown in Appendix B to be $\beta \mu_{\mathrm{dimer}}≃0.79$ at the physiological temperature of T≃310 K.

The average occupancy ${\langle n^{\left(\gamma \right)}\rangle }_{\mathrm{NI}}$ for species γ per site is found by taking the derivative of this partition function with respect to $\mu_{\gamma },$

(9) $${\langle n^{\left(\gamma \right)}\rangle }_{\mathrm{NI}}=\frac{1}{N_{\mathrm{sites}}\beta Z_{\mathrm{NI}}}\frac{\partial Z_{\mathrm{NI}}}{\partial \mu_{\gamma }}\;.$$

This can be verified by taking the derivative of Equation (8).

(10) $${\langle n^{\left(\gamma \right)}\rangle }_{\mathrm{NI}}=\frac{1}{N_{\mathrm{sites}}Z_{\mathrm{NI}}}\sum_{\mathrm{configurations}}\sum_{\ell ,b}n_{\ell ,b}^{\left(\gamma \right)}e^{-\beta \sum_{\ell ,b}\sum_{\gamma }\left(\varepsilon_{\gamma }-\mu_{\gamma }\right)n_{\ell ,b}^{\left(\gamma \right)}}\;,$$

which is by definition the average number per site of species γ.

Continuing with the evaluation of the partition function, we note that the exponential of a sum can be written as the product of exponentials, enabling us to rewrite the partition function (8) as

(11) $$Z_{\mathrm{NI}}=\sum_{\mathrm{configurations}}\prod_{\ell ,b}\prod_{\gamma }{\left[e^{-\beta \left(\varepsilon_{\gamma }-\mu_{\gamma }\right)}\right]}^{n_{\ell ,b}^{\left(\gamma \right)}}\;.$$

In order to simplify and to appreciate the physical meaning of this expression, it is useful to define the relative activity [40] of species γ in the noninteracting lattice gas model, given by

(12) $$a_{\mathrm{NI}}^{\left(\gamma \right)}=e^{-\beta \left(\varepsilon_{\gamma }-\mu_{\gamma }\right)}\;.$$

Note that this is independent of the lattice site *ℓ* or chain *b*. The partition function can then be written in the simple form

(13) $$Z_{\mathrm{NI}}=\sum_{\mathrm{configurations}}\prod_{\gamma }\prod_{\ell ,b}{\left[a_{\mathrm{NI}}^{\left(\gamma \right)}\right]}^{n_{\ell ,b}^{\left(\gamma \right)}}\;.$$

Because we have assumed that there can be only one species per site, parallel dimer, perpendicular dimer, or vacancy, the sum over configurations can now be performed over each site separately, where there are three possible configurations

(14) $$\left\{n_{\ell ,b}^{\left(⊥\right)},n_{\ell ,b}^{\left(\|\right)},n_{\ell ,b}^{\left(v\right)}\right\}=\left\{1,0,0\right\}\;,\;\left\{0,1,0\right\}\;,\;\mathrm{or}\;\left\{0,0,1\right\}\;.$$

This is the same as saying that $n_{\ell ,b}^{\left(\gamma \right)}=1$ for one and only one of γ=⊥, ‖, or *v*, and is zero otherwise. This means that

(15) $$\sum_{\begin{array}{c} \mathrm{single-site}\\ \mathrm{configurations} \end{array}}\prod_{\gamma =⊥,\|,v}{\left[a_{\mathrm{NI}}^{\left(\gamma \right)}\right]}^{n_{\ell ,b}^{\left(\gamma \right)}}=\sum_{\gamma }a_{\mathrm{NI}}^{\left(\gamma \right)}\;,$$

and so the grand partition function is

(16) $$Z_{\mathrm{NI}}=\prod_{\ell ,b}\left(\sum_{\gamma }a_{\mathrm{NI}}^{\left(\gamma \right)}\right)\;.$$

Since every term in the product is the same, the expression in parentheses is simply raised to the power $N_{\mathrm{sites}}$, giving

(17) $$Z_{\mathrm{NI}}={\left(\sum_{\gamma }a_{\mathrm{NI}}^{\left(\gamma \right)}\right)}^{N_{\mathrm{sites}}}\;.$$

At this point, we can easily see that we can obtain the average number per site using our derivative form from Equation (9) as

(18) $${\langle n^{\left(\gamma \right)}\rangle }_{\mathrm{NI}}=\frac{1}{N_{\mathrm{sites}}\beta Z_{\mathrm{NI}}}\frac{\partial }{\partial \mu_{\gamma }}{\left(\sum_{\gamma^{\prime }}a_{\mathrm{NI}}^{\left(\gamma^{\prime }\right)}\right)}^{N_{\mathrm{sites}}}\;.$$

Taking the derivative and substituting the expression for $Z_{\mathrm{NI}}$ in the denominator, we have

(19) $${\langle n^{\left(\gamma \right)}\rangle }_{\mathrm{NI}}=\frac{N_{\mathrm{sites}}}{N_{\mathrm{sites}}\beta {\left(\sum_{\gamma^{\prime \prime }}a_{\mathrm{NI}}^{\left(\gamma^{\prime \prime }\right)}\right)}^{N_{\mathrm{sites}}}}{\left(\sum_{\gamma^{\prime }}a_{\mathrm{NI}}^{\left(\gamma^{\prime }\right)}\right)}^{N_{\mathrm{sites}}-1}\frac{\partial }{\partial \mu_{\gamma }}a_{\mathrm{NI}}^{\left(\gamma \right)}\;,$$

where the derivative of the activity is

(20) $$\frac{\partial }{\partial \mu_{\gamma }}a_{\mathrm{NI}}^{\left(\gamma \right)}=\frac{\partial }{\partial \mu_{\gamma }}e^{-\beta \left(\varepsilon_{\gamma }-\mu_{\gamma }\right)}=\beta a_{\mathrm{NI}}^{\left(\gamma \right)}\;.$$

Using this in the expression for the mean site occupancy in Equation (19) and canceling $N_{\mathrm{sites}}-1$ factors of the sum over γ, the mean occupancy for species γ is given by

(21) $${\langle n^{\left(\gamma \right)}\rangle }_{\mathrm{NI}}=\frac{a_{\mathrm{NI}}^{\left(\gamma \right)}}{\sum_{\gamma^{\prime }}a_{\mathrm{NI}}^{\left(\gamma^{\prime }\right)}}\;.$$

**Figure 5:**
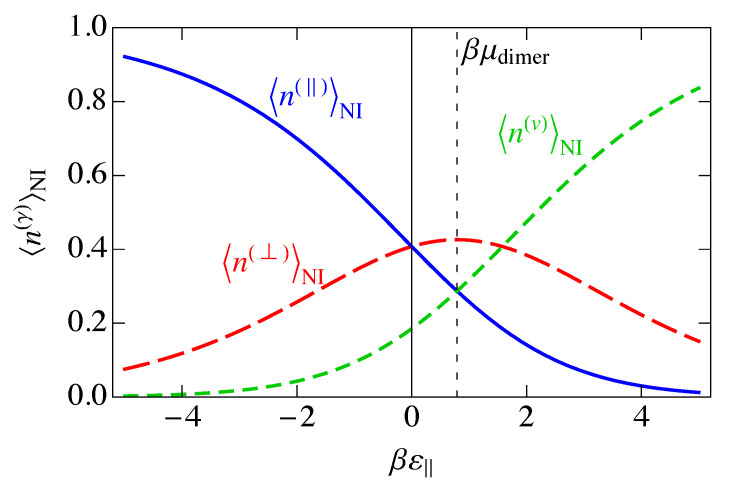
Plots of the mean occupation numbers ${\langle n^{\left(\gamma \right)}\rangle }_{NI}$ in the noninteracting lattice gas model. Curves are shown for γ=‖ (blue, solid), ⊥ (red, long-dashed), and *v* (green, short-dashed). The parameters used in the calculation are $\beta \mu_{\mathrm{dimer}}=0.79$ and $\varepsilon_{\|}=2\varepsilon_{⊥}$. For negative $\varepsilon_{\|}-\mu_{\mathrm{dimer}}$, dimers are attracted to the lattice, while for positive values of this quantity, they are repelled.

In Figure 5, we show the mean occupancies for the three species versus $\varepsilon_{\|}$, with $\varepsilon_{\|}=2\varepsilon_{⊥}$. Negative $\varepsilon_{\|}$ corresponds to an attraction of dimers to the lattice, and we see that parallel adsorption of dimers dominates because of the stronger binding, followed by perpendicular adsorption, with vacancies becoming nonexistent. Positive $\varepsilon_{\|}$ corresponds to a repulsion of dimers from the lattice, so that at large $\varepsilon_{\|}$, the ordering is reversed, for the same reasons. At small positive $\varepsilon_{\|}$, the dimers are repelled from the lattice, but this competes with the chemical potential, which tries to put dimers back onto the lattice. In this low-coverage regime, perpendicular adsorption dominates, while vacancies dominate for large $\beta \varepsilon_{\|}$ because the dimers would prefer to stay in solution. For $\varepsilon_{\|},\varepsilon_{⊥}\to 0$, the parallel and perpendicular mean occupancies become the same. Similarly, where $\varepsilon_{\|}-\mu_{\mathrm{dimer}}=0$, ${\langle n^{\left(\|\right)}\rangle }_{\mathrm{NI}}={\langle n^{\left(v\right)}\rangle }_{\mathrm{NI}}$ because, as mentioned earlier, $\varepsilon_{v}-\mu_{\mathrm{dimer}}$ is always zero. Also, ${\langle n^{\left(⊥\right)}\rangle }_{\mathrm{NI}}={\langle n^{\left(v\right)}\rangle }_{\mathrm{NI}}$ where $\varepsilon_{\|}=2\mu_{\mathrm{dimer}}$, because that is where $\varepsilon_{⊥}=\mu_{\mathrm{dimer}}$. It is at this point where ${\langle n^{\left(⊥\right)}\rangle }_{\mathrm{NI}}$ becomes a maximum.

The average charge per site can now be determined by multiplying $q_{\gamma }$, the charge of species γ, by $n_{\ell ,b}^{\left(\gamma \right)}$ the occupation of species γ on site *ℓ* of chain *b*,

(22) $$\rho_{\ell b}=\sum_{\gamma }q_{\gamma }n_{\ell b}^{\left(\gamma \right)}\;.$$

Since we assume that every site has either a parallel dimer, a perpendicular dimer, or a vacancy, with single occupancy, then for a given site and species, $n_{\ell b}^{\left(\gamma \right)}$ is either 0 or 1. The average charge per site is then the sum of the averages of the individual terms,

(23) $$\rho_{\mathrm{NI}}\equiv {\langle \rho_{\ell b}\rangle }_{\mathrm{NI}}=\sum_{\gamma }q_{\gamma }{\langle n^{\left(\gamma \right)}\rangle }_{\mathrm{NI}}\;.$$

**Figure 6:**
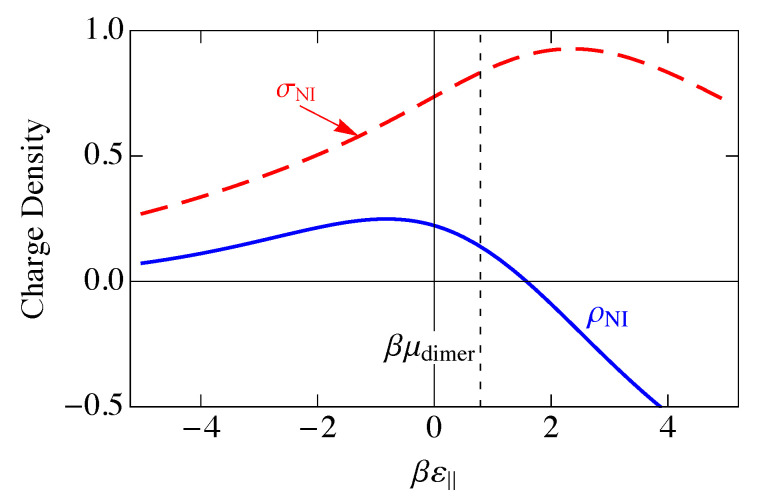
Plots of the charge per site for the noninteracting lattice gas model. The charge per site $\rho_{NI}$ is shown as the solid blue curve, and the standard deviation of the charge per site, $\sigma_{c}$, is shown as the dashed red curve. The parameters used in the calculation are $\beta \mu_{\mathrm{dimer}}=0.79$ and $\varepsilon_{\|}=2\varepsilon_{⊥}$. As in Figure 5, dimers are attracted to the lattice when $\varepsilon_{\|}-\mu_{\mathrm{dimer}}<0$, and dimers are repelled from the lattice when $\varepsilon_{\|}-\mu_{\mathrm{dimer}}>0$. Note that as $\beta \varepsilon_{\|}\to \infty$, all the dimers will detach from the DNA, and the charge per site $\rho_{NI}$ will approach −1.

This charge per site is plotted as the solid blue curve in Figure 6. Because a parallel dimer has no charge, $q_{\|}=0$, a perpendicular dimer has a positive unit charge, $q_{⊥}=+1$, and a vacancy has a negative unit charge, $q_{v}=-1$, the total mean-field charge per site $\rho_{c}$ in Figure 6 is the difference between the curves ${\langle n^{\left(⊥\right)}\rangle }_{\mathrm{NI}}$ and ${\langle n^{v}\rangle }_{\mathrm{NI}}$ in Figure 5 and goes to zero where those two curves cross.

A measure of the magnitude of the charge fluctuations is given by the average of the square of the charge on a site minus the square of its average, which is the charge variance $\sigma_{NI}^{2}$, given by

(24) $$\sigma_{\mathrm{NI}}^{2}={\langle \rho^{2}\rangle }_{\mathrm{NI}}-\rho_{\mathrm{NI}}^{2}\;,$$

where

(25) $${\langle \rho^{2}\rangle }_{\mathrm{NI}}={\langle \rho_{\ell b}^{2}\rangle }_{\mathrm{NI}}\;,$$

and

(26) $$\rho_{\ell b}^{2}={\left[\sum_{\gamma }q_{\gamma }n_{\ell b}^{\left(\gamma \right)}\right]}^{2}=\sum_{\gamma ,\gamma^{\prime }}q_{\gamma }q_{\gamma^{\prime }}n_{\ell b}^{\left(\gamma \right)}n_{\ell b}^{\left(\gamma^{\prime }\right)}\;.$$

The product $n_{\ell b}^{\left(\gamma \right)}n_{\ell b}^{\left(\gamma^{\prime }\right)}$ describes a double occupancy of site (ℓ,b) by species γ and $\gamma^{\prime }$. Since we have required single occupancy, then we must have $\gamma =\gamma^{\prime }$. Also, since $n_{\ell b}^{\left(\gamma \right)}$ can only take the values 0 or 1,

(27) $${\left[n_{\ell b}^{\left(\gamma \right)}\right]}^{2}=n_{\ell b}^{\left(\gamma \right)}\;.$$

Then, the charge per site reduces to

(28) $$\rho_{\ell b,\mathrm{NI}}^{2}=\sum_{\gamma }q_{\gamma }^{2}n_{\ell b,\mathrm{NI}}^{\left(\gamma \right)}\;.$$

Taking the thermal average of both sides, we have

(29) $${\langle \rho^{2}\rangle }_{\mathrm{NI}}\equiv {\langle \rho_{\ell b}^{2}\rangle }_{\mathrm{NI}}=\sum_{\gamma }q_{\gamma }^{2}{\langle n^{\left(\gamma \right)}\rangle }_{\mathrm{NI}}\;.$$

In Figure 6, we have plotted the standard deviation in the charge per site, $\sigma_{\mathrm{NI}}$, which is the square root of the charge variance, for the noninteracting model. As we saw in Figure 5, there are three distinct regions, $\varepsilon_{\|}\ll 0$, $\varepsilon_{\|}\gg 0$, and $\varepsilon_{\|}$ near $\mu_{\mathrm{dimer}}$. These three regions are also reflected in Figure 6. At large negative $\varepsilon_{\|}$, parallel adsorption dominates, which leads to ρ≈0. At large positive $\varepsilon_{\|}$, vacancies dominate, leading to ρ<0, and for $\varepsilon_{\|}$ near $\mu_{\mathrm{dimer}}$, there is a small window where perpendicular adsorption of dimers dominates, leading to positive values of ρ. Correspondingly, the fluctuations, represented by the standard deviation $\sigma_{NI}$, become small when $|\varepsilon_{\|}|$ becomes large, and the fluctuations are largest at small $|\varepsilon_{\|}|$, when there are comparable numbers of all species. The phenomenon of charge inversion is demonstrated in Figure 6 because the average charge is positive, indicating that sufficiently many dimers adsorb in a perpendicular configuration to invert the charge on the molecule from negative to positive. The magnitude of charge inversion increases in the weak-binding limit $\varepsilon_{⊥},\varepsilon_{\|}\to 0$. The maximum of this inversion in Figure 6 is about 0.25, which is much larger than the estimate of 0.07 from the work of Nguyen and Shklovskii [26], as discussed in the Introduction. That work treated the charge on DNA as a continuum, while we have used the discrete lattice of phosphates in a lattice gas model. We have not included interactions between sites yet, although we have assumed that there can only be one species per site, either a parallel dimer, a perpendicular dimer, or a vacancy. We will see later in this paper that interactions reduce this value somewhat, but it will still be much larger than 0.07.

The noninteracting entropy $S_{\mathrm{NI}}$ can be obtained from the partition function using Equation (7) as

(30) $$S_{\mathrm{NI}}=k_{B}\ln\left[{\left(\sum_{\gamma }a_{\mathrm{NI}}^{\left(\gamma \right)}\right)}^{N_{\mathrm{sites}}}\right]-\frac{k_{B}\beta }{{\left(\sum_{\gamma^{\prime }}a_{\mathrm{NI}}^{\left(\gamma^{\prime }\right)}\right)}^{N_{\mathrm{sites}}}}\frac{\partial }{\partial \beta }{\left(\sum_{\gamma }a_{\mathrm{NI}}^{\left(\gamma \right)}\right)}^{N_{\mathrm{sites}}}\;.$$

Because the derivative of the activity with respect to β can be written in terms of its logarithm as

(31) $$\beta \frac{\partial }{\partial \beta }a_{\mathrm{NI}}^{\left(\gamma \right)}=a_{\mathrm{NI}}^{\left(\gamma \right)}\ln a_{\mathrm{NI}}^{\left(\gamma \right)}\;,$$

the entropy can be written in the simple form

(32) $$\frac{S_{\mathrm{NI}}}{k_{B}N_{\mathrm{sites}}}=-\sum_{\gamma }\frac{a_{\mathrm{NI}}^{\left(\gamma \right)}}{\sum_{\gamma^{\prime \prime }}a_{\mathrm{NI}}^{\left(\gamma^{\prime \prime }\right)}}\ln\left(\frac{a_{\mathrm{NI}}^{\left(\gamma \right)}}{\sum_{\gamma^{\prime }}a_{\mathrm{NI}}^{\left(\gamma^{\prime }\right)}}\right)\;.$$

Substituting the mean occupancy for the noninteracting model from Equation (21) allows us to write the dimensionless entropy per site for the noninteracting model in a simplified form as

(33) $$\frac{S_{\mathrm{NI}}}{k_{B}N_{\mathrm{sites}}}=-\sum_{\gamma }{\langle n^{\left(\gamma \right)}\rangle }_{\mathrm{NI}}\ln{\langle n^{\left(\gamma \right)}\rangle }_{\mathrm{NI}}\;,$$

which agrees with the standard result for the entropy of mixing of an ideal solution with species γ=(‖,⊥,v) [40].

**Figure 7:**
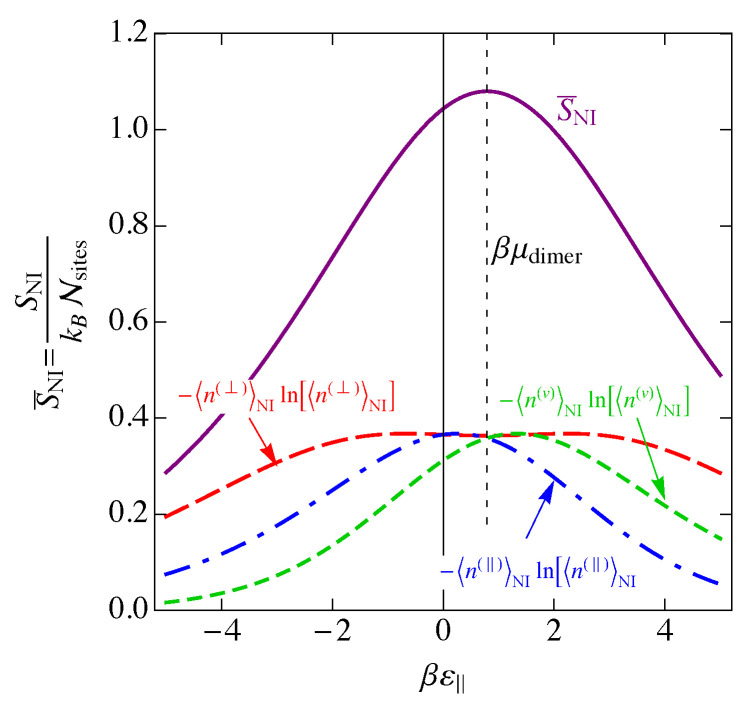
The entropy $S_{NI}$ per site in the noninteracting lattice gas model. The parameters used in the calculation are $\beta \mu_{\mathrm{dimer}}=0.79$ and $\varepsilon_{\|}=2\varepsilon_{⊥}$. The dashed curves are the individual contributions in Equation (33).

In Figure 7, we show the entropy $S_{NI}$ of the noninteracting model as a function of the binding energy $\beta \varepsilon_{\|}$, assuming $\varepsilon_{\|}=2\varepsilon_{⊥}$. We also show the individual contributions of Equation (33) to the entropy. The entropy is a maximum when the disorder is greatest, and this occurs when the numbers of each of the species are as close to equal as possible, which occurs at $\varepsilon_{\|}=\mu_{\mathrm{dimer}}$, the maximum of $\langle n^{\left(⊥\right)}\rangle$ in Figure 5.

## 3. Geometry of the Charged Double Helix of DNA

**Figure 8:**
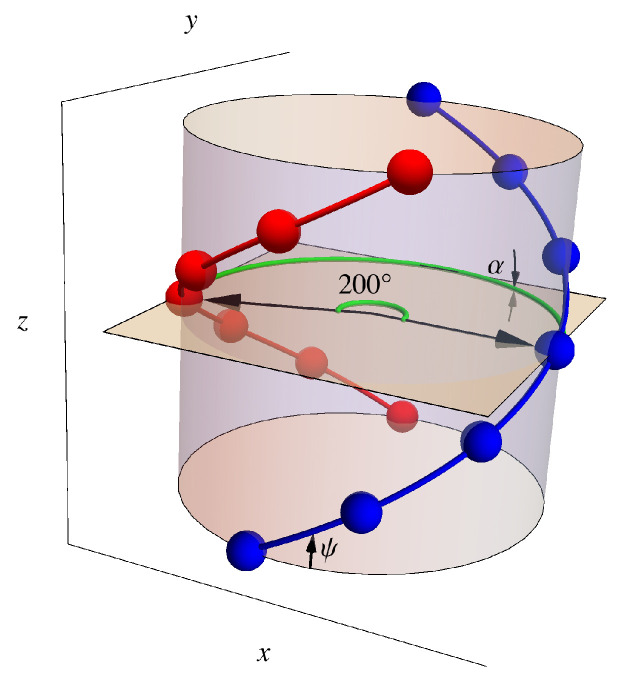
A segment of a single DNA strand, with the two helices depicted as chains of red and blue balls representing the phosphates, as in Figure 2. Each helix winds along the cylindrical surface with a pitch angle ψ, and a helix of pitch angle α connects a site with its neighbor on the other chain. The helices have been stretched in the *z*-direction so that the angle α, which is only ≈0.4${}^{\circ }$, can be clearly labeled on the diagram.

While stored in the nucleus of a cell, DNA is wrapped compactly both around histone protein complexes and around itself, but on sufficiently small scales (≈150 base pairs [41], or 15 turns [30], or 50 nm [8]), DNA’s dominant geometrical structure is the familiar double-helix structure shown in Figure 2. As explained in Bishop and McMullen [28], that structure can be constructed by wrapping a flexible ladder around a cylinder such that the ladder lies flat against the surface of the cylinder. Then, the lattice sites are the intersections between the rungs and the sides of the ladder. The position of each site is the mean position of the pair of protruding oxygens that are attached to each phosphorus, which incorporates the resonant structure represented by one electron being shared by two oxygens. These sites are considered to be the locations of negative point charges (−e, where *e* is the magnitude of the charge of the electron). The locations of the protruding oxygens are those given by the SYBYL molecular modeling software, version 6.9.2 (Tripos, Inc., St. Louis, MO, USA: www.tripos.com accessed on 15 June 2006) [42] for B-form DNA, which uses X-ray data [43,44,45]. This form of DNA was used because it is the most relevant physiologically. In the model shown, we used all adenine–thymine base pairs. The SYBYL program used a period of exactly ten base pairs for one complete turn of the helix, which means that the relative rotation angle between adjacent pairs was exactly $\Delta \phi =36^{\circ }$, or 1/10 of $360^{\circ }$. Each strand of the DNA then takes the shape of a helix with a characteristic radius $R_{\mathrm{DNA}}=0.946$ nm and pitch angle $\psi =29.6^{\circ }$, as shown in Figure 8. Here, the pitch angle ψ denotes the angle with respect to the xy-plane that gives the appropriate altitude per unit circumferential winding; in cylindrical coordinates, $\tan\psi =\Delta z/R_{\mathrm{DNA}}\Delta \phi$, where Δz is the distance along the *z*-axis. Each strand of DNA has a “direction”, identified by a particular carbon on the backbone structure, corresponding to the chirality of the helix. In the DNA double helix, the two strands are antiparallel and, therefore, have opposite chiralities. As a consequence, the azimuthal angle between the two helices is always a constant, $\Delta \phi =160^{\circ }$. Because this phase shift is not exactly $180^{\circ }$, the chains have unequal separation in the clockwise and counterclockwise azimuthal directions. The larger gap is referred to as the *major groove*, and the smaller as the *minor groove*.

The oxygen atoms occur at regular intervals along each strand, separated by a helix segment of arc length $a^{\left(1\right)}=0.684$ nm. It is these sites, located at regular intervals along the helix, to which dimers will adsorb. These negatively charged sites do not occur at the same altitudes on both strands, however. Rather, there is a vertical separation Δz=0.023 nm between corresponding sites on the two strands. With the relative phase of the strands and the vertical separation between sites on those strands taken together, corresponding sites on the two strands may be viewed as connected by a helical segment of arc length $a^{\left(2\right)}=3.34$ nm at a pitch angle of $\alpha =0.394^{\circ }$. This geometry is shown in Figure 8.

When positively charged dimers approach the DNA molecule, they will be attracted to the negative charges at the sites on the double helix. We consider dimers with a length comparable to the spacing $a^{\left(1\right)}$ between sites on a strand and having positive charges +e at either end. The dimers can then adsorb onto the surface in two possible orientations, either parallel to the strand or perpendicular to the helix axis (see Figure 4). If the dimer adsorbs parallel to the strand, the positive charges from the dimer lie directly over the negative charges on the strand, neutralizing the charge on two adjacent sites. If the dimer adsorbs perpendicular to the surface of the bounding cylinder, one end of the dimer sits atop the site, while the other extends radially outward. This perpendicular adsorption effectively inverts the charge on the site from −e to +e. In order to use a lattice gas model, this geometric constraint is loosened by having the parallel dimer block only a single site. This deficiency can be somewhat compensated for by making the binding energy of the parallel dimer twice as large, $\varepsilon_{\|}\approx 2\varepsilon_{⊥}$.

Note that because the length of the dimer (equal to the same-chain site spacing $a^{\left(1\right)}=0.684$ nm) is much smaller than both the cross-chain site spacing, $a^{\left(2\right)}=3.34$ nm, and the vertical separation, Δz=3.4 nm, between turns of the helix, other orientations of the dimer are not possible.

The problem thus described is a complex one, but the similarities with the lattice gas models of condensed matter physics provide guidelines for how to proceed. These prescriptions, however, are aimed at the treatment of a periodic crystalline lattice, and, although the DNA sites exhibit helical symmetry, they do not constitute a periodic lattice in the strict sense of the term. However, an appropriate choice of coordinates can take advantage of the helical symmetry, so that, in these new coordinates, the positions of the sites will fall on a regular one-dimensional lattice.

**Figure 9:**
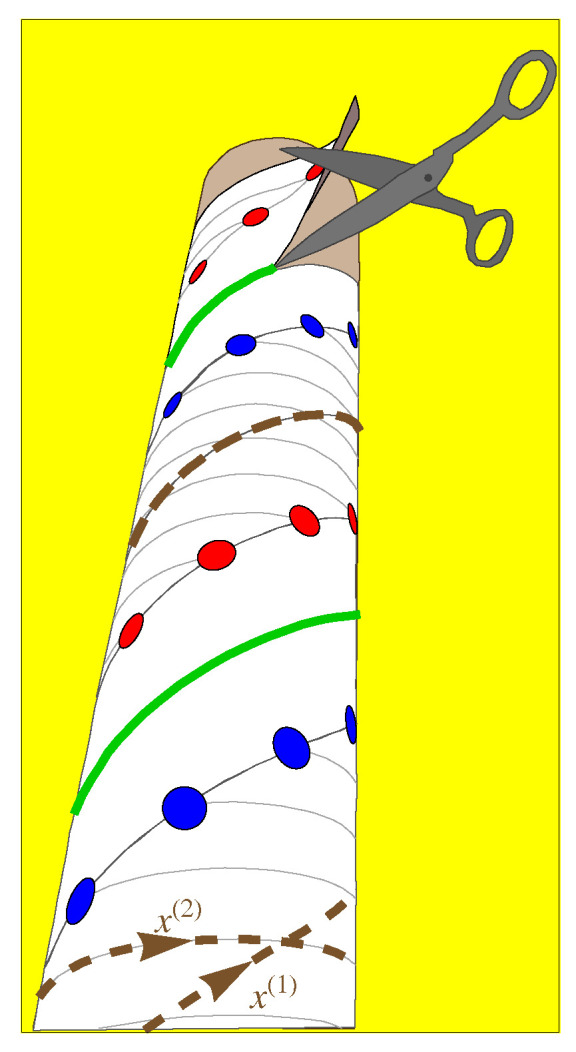
DNA double helix wrapped around a cylindrical tube, with the “up” (↑) chain phosphates depicted as red balls, and the “down” (↓) chain phosphates depicted as blue balls, as in Figure 2 and Figure 8. If the scissors cut along the solid green line, which is the center of the minor groove, then the paper can be put on a flat surface, with the double helix forming a one-dimensional chain. The coordinate $x^{\left(1\right)}$ lies along the dashed brown line in the major groove, midway between the up and down chains, and $x^{\left(2\right)}$ lies along a path on the surface of the cylinder that connects the two phosphates on the two sub-chains that are connected by base pairs.

**Figure 10:**
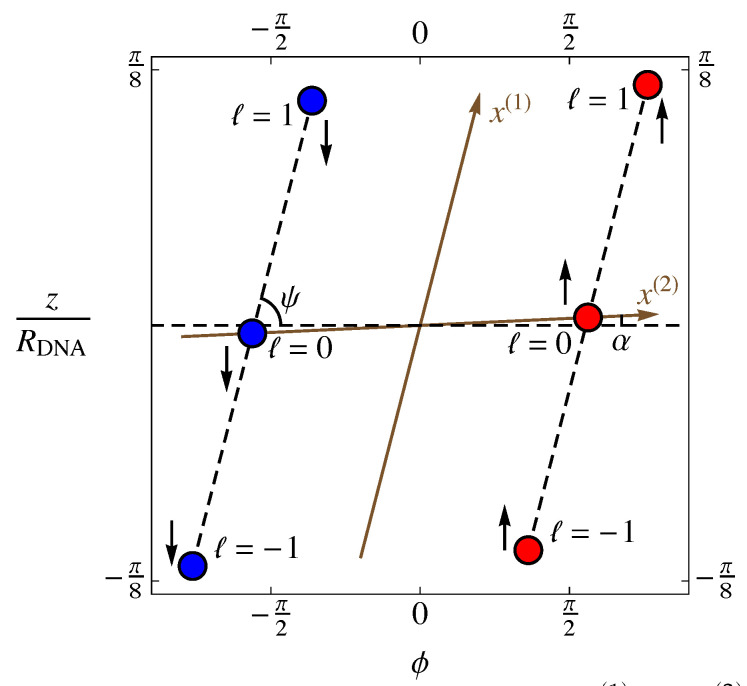
Diagram of the new coordinates $x^{\left(1\right)}$ and $x^{\left(2\right)}$ in terms of the cylindrical coordinates (ϕ,z). As in Figure 2, Figure 8 and Figure 9, the “up” (↑) chain phosphates are shown as red balls, and the “down” (↓) chain phosphates are shown as blue balls.

We will define these coordinates on a cylinder of radius $R_{DNA}$, as shown in Figure 8 and Figure 9. The first coordinate $x^{\left(1\right)}$ traces out a path with pitch angle ψ along a single helical strand, and the other coordinate $x^{\left(2\right)}$ traces out a path with pitch angle α that connects corresponding sites on the two strands. Geometrically, a cylinder can be regarded as flat in the sense that it has no curvature. In Figure 9, we show the way that the cylinder can be cut with scissors and unwrapped so that this lattice can be mapped on a flat surface as shown in Figure 10. If we define the origin of coordinates $x^{\left(1\right)}$ and $x^{\left(2\right)}$ to be halfway along the helical path between the partners on the two chains, as shown in Figure 9 and Figure 10, then the positions of the sites on both strands form a one-dimensional lattice in the coordinates $\left(x^{\left(1\right)},x^{\left(2\right)}\right)$.

These coordinates can be written simply in terms of the cylindrical coordinates (ϕ,z) in matrix form as

(34) $$\left[\begin{array}{c} R_{\mathrm{DNA}}\phi \\ z \end{array}\right]=\left[\begin{array}{cc} \cos\psi & \cos\alpha \\ \sin\psi & \sin\alpha \end{array}\right]\left[\begin{array}{c} x^{\left(1\right)}\\ x^{\left(2\right)} \end{array}\right].$$

Inverting this gives definitions of the two coordinates as

(35) $$x^{\left(1\right)}=\left(\frac{\sin\alpha }{\sin(\alpha -\psi )}\right)R_{\mathrm{DNA}}\phi -\left(\frac{\cos\alpha }{\sin(\alpha -\psi )}\right)z$$

and

(36) $$x^{\left(2\right)}=-\left(\frac{\sin\psi }{\sin(\alpha -\psi )}\right)R_{\mathrm{DNA}}\phi +\left(\frac{\cos\psi }{\sin(\alpha -\psi )}\right)z.$$

With these definitions, the difference in coordinates between adjacent sites on the same strand is $\Delta x^{\left(1\right)}=a^{\left(1\right)}$, and the difference in coordinates between corresponding sites on the two strands is $\Delta x^{\left(2\right)}=a^{\left(2\right)}$. That is, $a^{\left(1\right)}$ is the distance along the helical path of a single chain from one phosphate ion to the next, and $a^{\left(2\right)}$ is the distance along a helical path from a phosphate ion on one chain to its partner phosphate ion on the other chain.

**Figure 11:**
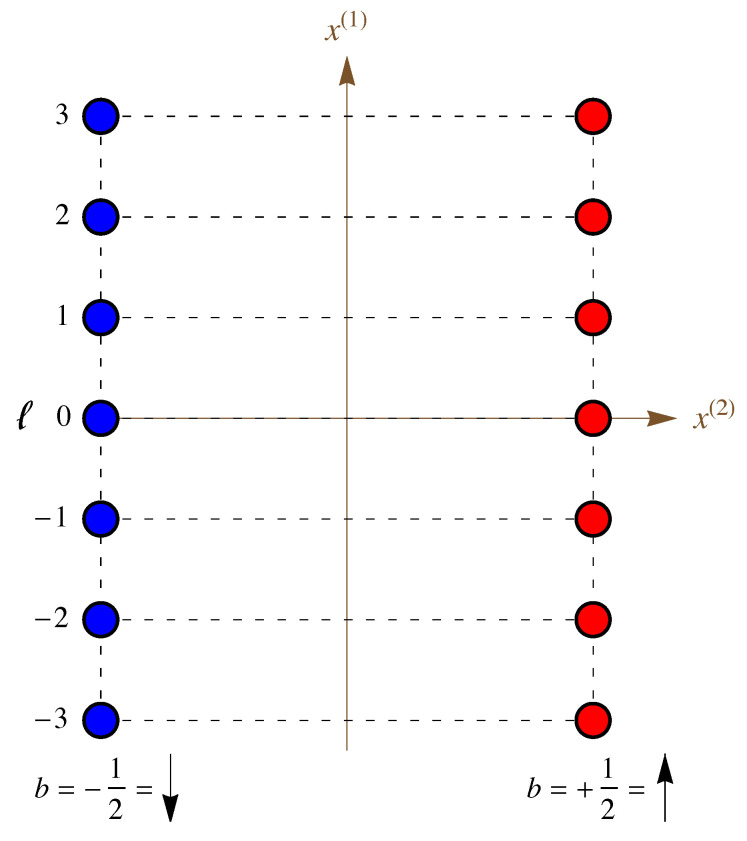
Unwinding the paper wrapped on the cylindrical tube in Figure 9 produces the “ladder” shown here. In the new coordinates $x^{\left(1\right)}$ and $x^{\left(2\right)}$, the positions of the sites constitute a one-dimensional lattice with two sites per unit cell. Here, $b=-\frac{1}{2}$ for the “down” (↓) chain of blue balls and $b=\frac{1}{2}$ for the “up” (↑) chain of red balls.

Next, we define a lattice index *ℓ*, which specifies the cell (altitude on the double helix), and chain index *b*, which specifies the basis site, where $b=-\frac{1}{2}$ for the “down” (↓) chain and $b=\frac{1}{2}$ for the “up” (↑) chain, as shown in Figure 11. Using these variables, the coordinates can be written as

(37) $$\left(x^{\left(1\right)},x^{\left(2\right)}\right)=\left(\ell a^{\left(1\right)},ba^{\left(2\right)}\right)\;,$$

where

(38) $$\ell =0,\pm 1,\pm 2,\ldots ;\;b=\pm \frac{1}{2}\;.$$

Thus, although the sites on the DNA molecule do not constitute a periodic lattice in real space, they do constitute a lattice in an appropriately defined coordinate space (see Figure 11). As we will see, however, this choice of coordinates will make the form of the interaction potential more complicated as a result.

The use of $\left(x^{\left(1\right)},x^{\left(2\right)}\right)$ instead of the cylindrical coordinates (ϕ,z) indicates a more fundamental shift in our description of the DNA double helix. The two-dimensional surface on which the helices lie is a cylinder of radius $R_{DNA}$, and the helices inherit the cylinder’s geometric properties. The geometry of the cylinder, however, is locally indistinguishable from the geometry of the flat plane. One common consequence of this is that it is possible to smoothly wrap a flat sheet of paper around a cylinder. In contrast, it is not possible to smoothly wrap a sheet of paper around a sphere; this problem is well known because of the geometrical distortions that occur in flat maps of a spherical Earth. Maps of a cylindrical surface, however, have no such distortions. This geometrical difference is quantified by the Riemannian curvature tensor, which vanishes for both the cylinder and the plane, but not for the sphere [46]. This means that the local geometry of the cylinder behaves in exactly the same way as the local geometry of the plane, so that a helix on a cylinder is geometrically equivalent to a line on a plane.

Our choice of coordinates is simply a map of the cylindrical surface that reduces the double helix to two parallel lines, as shown in Figure 11. The result of this map is that we have a linear lattice with two phosphate sites per cell, with the cells labeled from ℓ=−N to N. All 2N+1 cells are identical, and the lattice can be imagined to satisfy periodic boundary conditions.

The analogy describing DNA as a ladder wrapped around a cylinder of radius $R_{\mathrm{DNA}}$ then has a true mathematical basis, because the local structure of the double helix is equivalent to the structure of a “ladder”—a one-dimensional chain with a unit cell containing one site from each strand. If interactions are ignored, then the problem is described simply by this one-dimensional lattice.

## 4. DNA Interaction Potential

The geometrical structure of the double helix is important in determining the electrostatic energy $U_{\mathrm{int}}$ of the sites (with and without dimers) interacting with one another. Any thermal fluctuations in the positions of the charged lattice sites are omitted for simplicity. The full Hamiltonian must contain these contributions and can be written as

(39) $$H=\sum_{\ell ,b}\sum_{\gamma }\varepsilon_{\gamma }n_{\ell ,b}^{\left(\gamma \right)}+U_{\mathrm{int}}\;,$$

where the interaction energy $U_{\mathrm{int}}$ is the total interaction energy between all the charges on all the sites. If $V_{\left(\ell_{1},b_{1}\right),\left(\ell_{2},b_{2}\right)}$ is the screened Coulomb energy between a charge $q_{\gamma_{1}}$ at site $\ell_{1}$ on chain $b_{1}$ with another charge $q_{\gamma_{2}}$ at site $\ell_{2}$ on chain $b_{2}$, then $U_{\mathrm{int}}$ can be written as

(40) $$U_{\mathrm{int}}=\frac{1}{2}\sum_{\gamma_{1},\gamma_{2}}\sum_{\left(\ell_{1},b_{1}\right),\left(\ell_{2},b_{2}\right)}q_{\gamma_{1}}n_{\ell_{1},b_{1}}^{\left(\gamma_{1}\right)}V_{\left(\ell_{1},b_{1}\right),\left(\ell_{2},b_{2}\right)}n_{\ell_{2},b_{2}}^{\left(\gamma_{2}\right)}q_{\gamma_{2}}\;,$$

where $n_{\ell ,b}^{\left(\gamma \right)}$ is the number of particles of species γ on the lattice site at (ℓ,b) and $q_{\gamma }$ is the charge of that species, in units of the magnitude *e* of the charge of an electron. The factor of 1/2 is to ensure that we are not double counting when we sum over all lattice sites, and we implicitly exclude same-site interactions $\left(\ell_{1},b_{1}\right)\neq \left(\ell_{2},b_{2}\right)$. The total charge on a given site is given by summing all the charges on that site, so that the total charge on the site (ℓ,b) is

(41) $$\rho_{\ell ,b}=\sum_{\gamma }q_{\gamma }n_{\ell ,b}^{\left(\gamma \right)}\;.$$

The interaction potential can then be written in terms of the total charge on each site as

(42) $$U_{\mathrm{int}}=\frac{1}{2}\sum_{\left(\ell_{1},b_{1}\right),\left(\ell_{2},b_{2}\right)}\rho_{\left(\ell_{1},b_{1}\right)}V_{\left(\ell_{1},b_{1}\right),\left(\ell_{2},b_{2}\right)}\rho_{\left(\ell_{2},b_{2}\right)}\;,$$

where V is the electrostatic interaction energy between the sites. This screened electrostatic energy between charges $q_{\gamma_{1}}$ and $q_{\gamma_{2}}$ at positions $\left(\ell_{1},b_{1}\right)$ and $\left(\ell_{2},b_{2}\right)$ (in SI units) is

(43) $$V_{\left(\ell_{1},b_{1}\right),\left(\ell_{2},b_{2}\right)}=\frac{e^{2}}{4\pi \epsilon }\frac{e^{-q_{s}d_{b_{1}b_{2}}\left(\ell_{1}-\ell_{2}\right)}}{d_{b_{1}b_{2}}\left(\ell_{1}-\ell_{2}\right)}\;,$$

where ϵ is the electric permittivity of the medium between the two charges, and $d_{b_{1}b_{2}}\left(\ell_{1}-\ell_{2}\right)$ is the straight-line distance between the two charges. Distances along the chain are invariant under translations by a lattice spacing, and so $d_{b_{1}b_{2}}\left(\ell_{1}-\ell_{2}\right)$ depends only on the difference between the lattice site indices $\ell_{1}-\ell_{2}$. As in the noninteracting lattice gas model, we have three species of “particles” on the lattice sites, vacancies of charge $q_{v}=-1$, parallel (‖) dimers of charge $q_{\|}=0$, and perpendicular (⊥) dimers of charge $q_{⊥}=+1$.

Under physiological conditions, the presence of monovalent salt ions such as Na${}^{+}$ leads to screening of the bare charges. Traditionally, screening is treated by modeling the ions as a continuous density, resulting in the nonlinear Poisson–Boltzmann equation [19,47]. The Poisson–Boltzmann equation is not analytically solvable, so it is often further approximated by linearization. The resulting Thomas–Fermi model is analytically solvable and gives the screened Coulomb (or Yukawa) potential between two charges given in Equation (43), where $\epsilon =78.5\epsilon_{0}$ is the permittivity of water (with $\epsilon_{0}$ the permittivity of free space) and $q_{s}$ is the magnitude of the screening wave vector [48]. Various names are ascribed to both the nonlinear and linearized equations, including Debye–Hückel, Thomas–Fermi, and Poisson–Boltzmann, but we will always refer to the Poisson–Boltzmann equation when we mean the nonlinear form and the (linearized) Thomas–Fermi equation when we mean the linearized form.

One must be cautious in using the screened Coulomb potential for screened interactions. The continuous density approximation from which the nonlinear Poisson–Boltzmann equation was derived constitutes a form of mean-field theory [19], which fails to describe any of the effects due to correlations between the ions such as charge inversion and condensation. Thus, we cannot model screening of the DNA molecule by the dimers using the Thomas–Fermi model, or even the Poisson–Boltzmann equation. Instead, we must treat the dimers as individual particles and compute their interactions so that we do not ignore correlation effects.

For the screening due to the monovalent salt, however, the valence involved is small enough and the concentration is low enough that a mean-field treatment is generally believed to be a good approximation [19,49]. Thus, we will treat the interaction energy $U_{\mathrm{int}}$ in Equation (42) using the screened Coulomb potential for the dimer–dimer interactions, with the screening vector $q_{s}$ given in the Thomas–Fermi model as [48]

(44) $$q_{s}=\sqrt{2n\frac{\beta e^{2}}{\epsilon }}=\sqrt{8\pi n\ell_{B}}\;,$$

where *n* is the concentration of ions and $\ell_{B}=\beta e^{2}/\left(4\pi \epsilon \right)$ is the Bjerrum length [32]. The Bjerrum length is a characteristic length scale equal to the distance at which two proton charges interact with energy $k_{B}T$. It should be noted that these expressions differ slightly in the literature because of various authors’ choices of units for the electrostatics. Here, we use SI units. We assume that the DNA is submerged in a 0.1 molar NaCl solution and that the dielectric constant is $\kappa =\frac{\epsilon }{\epsilon_{0}}=78.5$ and the temperature is *T* = 37 ${}^{\circ }$C = 310 K. For these numbers, the Bjerrum length is $\ell_{B}=0.79$ nm and the screening length is $q_{s}^{-1}=0.98$ nm.

By choosing a coordinate system based on the arc lengths around the surface of the cylinder, we have managed to align the sites into a regular lattice, but the potential $V_{\left(\ell_{1},b_{1}\right),\left(\ell_{2},b_{2}\right)}$ depends on the *straight-line* distance between the sites rather than the surface arc-length connecting them. We must then express Equation (43) in terms of the straight-line distance function $d_{b_{1}b_{2}}\left(\ell_{1}-\ell_{2}\right)$ between the sites at $\left(\ell_{1}a^{\left(1\right)},b_{1}a^{\left(2\right)}\right)$ and $\left(\ell_{2}a^{\left(1\right)},b_{2}a^{\left(2\right)}\right)$. The straight-line distance in cylindrical coordinates between two points on the surface of a cylinder of radius $R_{\mathrm{DNA}}$ is

(45) $$\mathrm{distance}\left(\phi_{1},z_{1},\phi_{2},z_{2}\right)={\left\{2R_{\mathrm{DNA}}^{2}\left[1-\cos\left(\phi_{1}-\phi_{2}\right)\right]+{\left(z_{1}-z_{2}\right)}^{2}\right\}}^{\frac{1}{2}},$$

and applying our change in variables (35) and (36) gives the straight-line distance between sites as

(46) $$\begin{array}{ccc} d_{b_{1}b_{2}}\left(\ell \right) & = & R_{\mathrm{DNA}}\left\{2\left[1-\cos\left(\frac{a^{\left(1\right)}}{R_{\mathrm{DNA}}}\ell \cos\psi +\frac{a^{\left(2\right)}}{R_{\mathrm{DNA}}}b\cos\alpha \right)\right]\right.\\ & & +{\left.{\left[\frac{a^{\left(1\right)}}{R_{\mathrm{DNA}}}\ell \sin\psi +\frac{a^{\left(2\right)}}{R_{\mathrm{DNA}}}b\sin\alpha \right]}^{2}\right\}}^{\frac{1}{2}}\;, \end{array}$$

where $\ell =\ell_{1}-\ell_{2}$ and $b=b_{1}-b_{2}$. Because of the translational invariance in the lattice, the distance in Equation (46) depends only on the differences between the lattice and basis indices, and not their values individually. From this relation, we see the symmetry property of the distance,

(47) $$\begin{array}{cc} d_{b_{1}b_{2}}\left(\ell \right) & =d_{b_{2}b_{1}}\left(-\ell \right) \end{array}$$

(48) $$\begin{array}{cc} d_{↑↑}\left(\ell \right) & =d_{↓↓}\left(-\ell \right)\;. \end{array}$$

**Figure 12:**
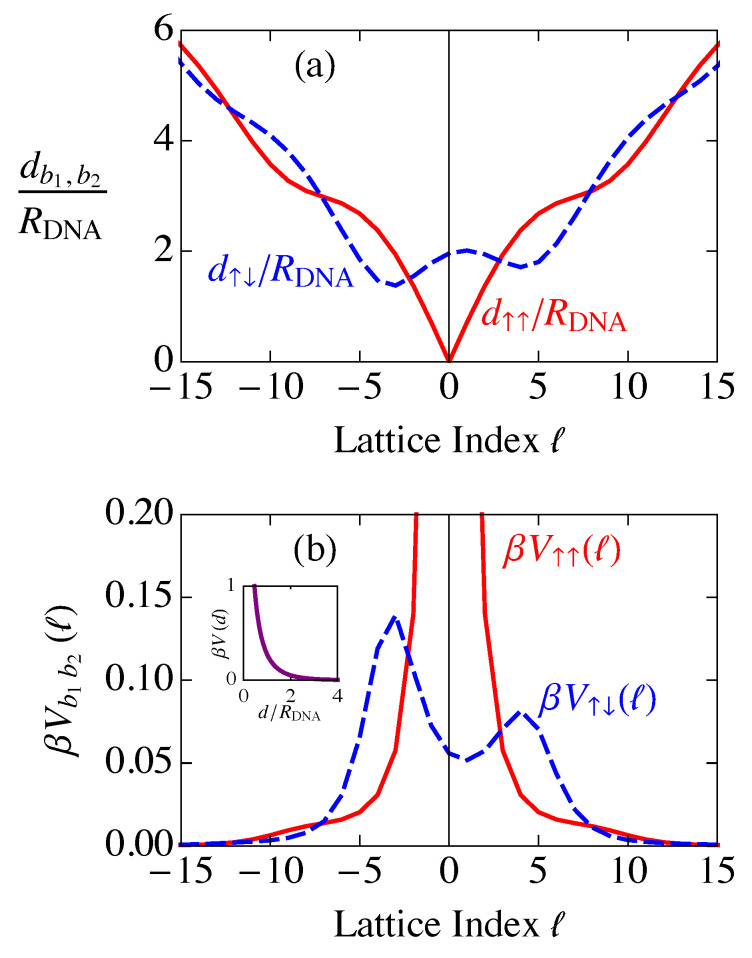
(**a**) Distance $d_{b_{1}b_{2}}\left(\ell \right)/R_{\mathrm{DNA}}$ between sites, and (**b**) screened Coulomb potential $V_{b_{1}b_{2}}\left(\ell_{1}-\ell_{2}\right)$ as a function of lattice index *ℓ*, within the same chain (↑↑) and between the two chains (↑↓). The red curve (↑↑), representing same-chain repulsion, is an even function, and the blue curve (↑↓), representing opposite-chain repulsion, is neither even nor odd. The point ℓ=0 for the same chain repulsion is omitted because it would correspond to a site interacting with itself. The inset in (**b**) is the screened Coulomb interaction as a function of the straight-line distance *d* between charges. At the physiological temperature of T=37 ${}^{\circ }$C (300.15 K), $\beta \equiv 1/k_{B}T\approx$ 1/(27 meV).

Since $b_{1}$ and $b_{2}$ are either $+\frac{1}{2}$ or $-\frac{1}{2}$, $b=b_{1}-b_{2}$ takes the three values b=0 for interactions of one site on a single chain with all other sites on the same chain, b=1 for interactions between a site on the “up” chain with with all the sites on the “down” chain, and b=−1 for interactions of one site on the “down” chain with all the sites on the “up” chain. We will use an up arrow ↑ to indicate $b_{1,2}=\frac{1}{2}$, and a down arrow ↓ to indicate $b_{1,2}=-\frac{1}{2}$. Plots of $d_{↑↑}\left(\ell \right)/R_{\mathrm{DNA}}$ and $d_{↑↓}\left(\ell \right)/R_{\mathrm{DNA}}$ are shown in Figure 12a. There are wiggles in the curve because, as the chain wraps around the cylinder (see Figure 4), the distances between lattice sites can become larger or smaller than they would be on a linear chain.

In order to understand the consequences of the screened Coulomb potential (43) depending on the straight-line distances *d* between sites, we define a new notation for the potential, which is expressed in terms of the difference in lattice site indices $\ell_{1}-\ell_{2}$,

(49) $$V_{b_{1}b_{2}}\left(\ell_{1}-\ell_{2}\right)\equiv V_{\left(\ell_{1},b_{1}\right),\left(\ell_{2},b_{2}\right)}\;.$$

This potential decays exponentially as a function of the straight-line distance *d* (inset of Figure 12b). However, as a function of the lattice index *ℓ*, as in Figure 12b, there are corresponding “wiggles” in the decay. This occurs because the distance between the sites decreases slightly as the helix completes a turn. These wiggles are present for every turn the helix makes, but the effect on the potential becomes negligibly small after about the first turn. Thus, by using the indices *ℓ* and *b* to describe the relationships between sites, we have reduced the DNA double-helix structure to a regular one-dimensional lattice with two sites per cell at the cost of an irregular potential due to the geometrical structure of the DNA molecule. The potentials for other combinations of $b_{1}$ and $b_{2}$ can be related to those in Figure 12b through the symmetry relations

(50) $$\begin{array}{cc} V_{b_{1}b_{2}}\left(\ell \right) & =V_{b_{2}b_{1}}\left(-\ell \right) \end{array}$$

(51) $$\begin{array}{cc} V_{↑↑}\left(\ell \right) & =V_{↓↓}\left(-\ell \right)\;. \end{array}$$

Fourier transforming the site index *ℓ* block-diagonalizes this matrix into 2×2 blocks, corresponding to the chain indices $b_{1}$ and $b_{2}$. Explicitly, the Fourier transform is

(52) $$\tilde{V}=F^{†}VF\;,$$

where the elements of F are independent of $b_{1}$ and $b_{2}$ and are given by

(53) $$F_{\left(\ell ,b_{1}\right),\left(k,b_{2}\right)}=\frac{1}{\sqrt{2N+1}}e^{ik\ell }\;,$$

and the dagger denotes the adjoint (complex conjugate transpose). We use periodic boundary conditions so that the wave vector *k* appearing here, which is dimensionless, takes the 2N+1 values

(54) k=−NΔk⋯NΔk,

where

(55) $$\Delta k=\left(\frac{2\pi }{2N+1}\right)\;.$$

If N is large, the range of *k* is essentially continuous from −π to π.

The matrix elements of this Fourier transform are

(56) $${\tilde{V}}_{\left(k_{1},b_{1}\right),\left(k_{2},b_{2}\right)}=\frac{1}{2N+1}\sum_{\ell_{1},\ell_{2}=-N}^{N}e^{-ik_{1}\ell_{1}}V_{\left(\ell_{1}b_{1}\right),\left(\ell_{2},b_{2}\right)}e^{ik_{2}\ell_{2}}\;.$$

Substituting for V from Equation (49), we have

(57) $${\tilde{V}}_{\left(k_{1},b_{1}\right),\left(k_{2},b_{2}\right)}=\frac{1}{2N+1}\sum_{\ell_{1},\ell_{2}=-N}^{N}e^{-i(k_{1}\ell_{1}-k_{2}\ell_{2})}V_{b_{1}b_{2}}\left(\ell_{1}-\ell_{2}\right)\;.$$

Since we regard the chain as having periodic boundary conditions, changing the summation indices to $\ell =\ell_{1}-\ell_{2}$ and $\ell_{2}$ gives

(58) $${\tilde{V}}_{\left(k_{1},b_{1}\right),\left(k_{2},b_{2}\right)}=\frac{1}{2N+1}\sum_{\ell =-N}^{N}e^{-ik_{1}\ell }V_{b_{1}b_{2}}\left(\ell \right)\sum_{\ell_{2}=-N}^{N}e^{-i\left(k_{1}-k_{2}\right)\ell_{2}}\;,$$

where the sum over $\ell_{2}$ becomes

(59) $$\sum_{\ell_{2}=-N}^{N}e^{-i\left(k_{1}-k_{2}\right)\ell_{2}}=\left(2N+1\right)\delta_{k_{1},k_{2}}\;.$$

We can now write the Fourier transform of $V_{b_{1}b_{2}}\left(\ell \right)$ as

(60) $${\tilde{V}}_{b_{1}b_{2}}\left(k\right)=\sum_{\ell =-N}^{N}e^{-ik\ell }V_{b_{1}b_{2}}\left(\ell \right)\;.$$

Substituting this back into the matrix, we have

(61) $${\tilde{V}}_{\left(k_{1},b_{1}\right),\left(k_{2},b_{2}\right)}=\delta_{k_{1},k_{2}}{\tilde{V}}_{b_{1}b_{2}}\left(k_{1}\right)\;.$$

Thus, the Fourier transform $\tilde{V}$ of V is diagonal in the *k* indices and contains 2×2 blocks

(62) $$\tilde{V}\left(k\right)=\left[\begin{array}{cc} {\tilde{V}}_{↓↓}\left(k\right) & {\tilde{V}}_{↓↑}\left(k\right)\\ {\tilde{V}}_{↑↓}\left(k\right) & {\tilde{V}}_{↑↑}\left(k\right) \end{array}\right]\;.$$

The symmetries (50) of the screened Coulomb potential on the DNA lattice are reflected in the Fourier transforms (60) as well:

(63) $$\begin{array}{cc} {\tilde{V}}_{b_{1}b_{2}}\left(k\right) & ={\tilde{V}}_{b_{2}b_{1}}{\left(k\right)}^{*}={\tilde{V}}_{b_{2}b_{1}}\left(-k\right) \end{array}$$

(64) $$\begin{array}{cc} {\tilde{V}}_{↑↑}\left(k\right) & ={\tilde{V}}_{↓↓}\left(k\right)\;. \end{array}$$

**Figure 13:**
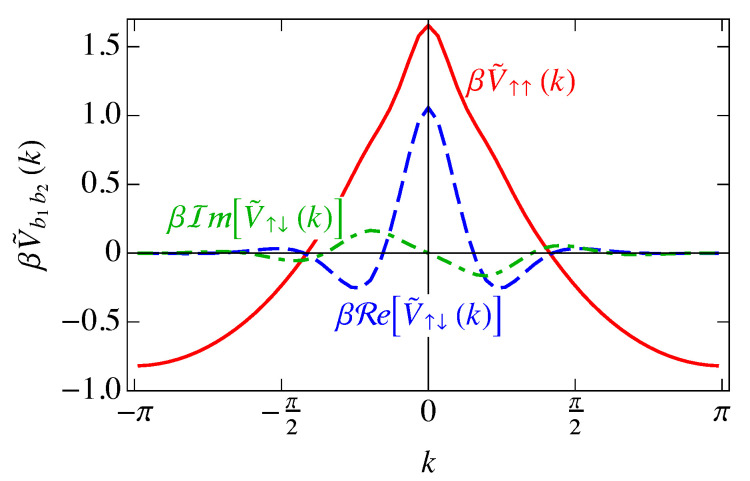
Plots of the Fourier transforms ${\tilde{V}}_{↑↑}\left(k\right)$ (solid red curve) and ${\tilde{V}}_{↑↓}\left(k\right)$ against the dimensionless wave vector *k*. The symbols Re and Im correspond to the real (blue dashed curve) and imaginary parts (green dot-dashed curve) of the complex Fourier transform, respectively.

Also, because $V_{↑↑}\left(\ell \right)$ is an even function of *ℓ*, ${\tilde{V}}_{↑↑}\left(k\right)$ is real. The Fourier transforms (60) are plotted in Figure 13.

As can be seen in Figure 13, the Fourier transforms $\tilde{V}\left(k\right)$ have regions of *k* that are negative, which is a result of transforming with respect to our helical coordinate system. When the screened Coulomb potential (43) is Fourier transformed with respect to the straight-line distance *d*, the function is positive definite. We have instead Fourier transformed the potential as a function of the lattice index *ℓ*, resulting in the “wiggles” in Figure 12b caused by the turns of the helix. These deviations from the exponential decay of V(d) result in the deviations here from the positive-definite Fourier transform.

Using the symmetry operations in Equations (63) and (64) obeyed by the Fourier transforms allows us to simplify the 2×2 blocks $\tilde{V}\left(k\right)$ in Equation (62) to

(65) $$\tilde{V}\left(k\right)=\left[\begin{array}{cc} {\tilde{V}}_{↑↑}\left(k\right) & {\tilde{V}}_{↑↓}^{*}\left(k\right)\\ {\tilde{V}}_{↑↓}\left(k\right) & {\tilde{V}}_{↑↑}\left(k\right) \end{array}\right].$$

**Figure 14:**
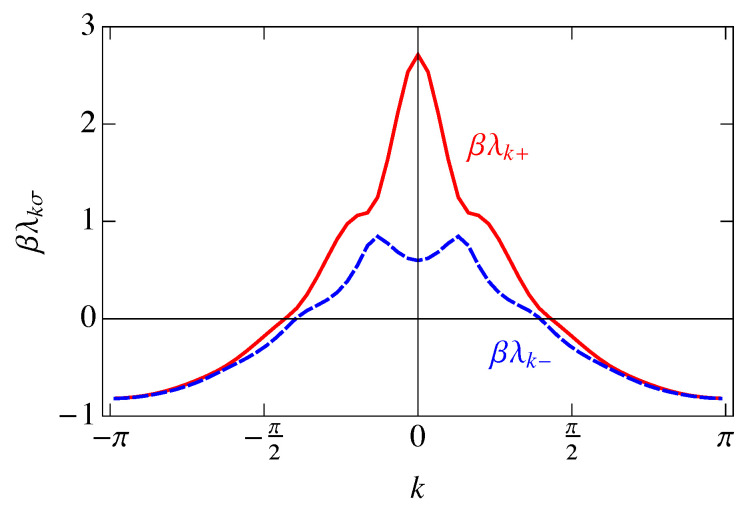
Plots of the eigenvalues $\lambda_{k\pm }$ of the 2×2 blocks of $\tilde{V}\left(k\right)$.

This matrix is easily diagonalized, yielding the real eigenvalues

(66) $$\lambda_{k,\pm }={\tilde{V}}_{↑↑}\left(k\right)\pm \left|{\tilde{V}}_{↑↓}\left(k\right)\right|,$$

which are plotted in Figure 14. The transformation that diagonalizes $\tilde{V}\left(k\right)$ is a unitary matrix ξ(k) whose columns are the eigenvectors of $\tilde{V}\left(k\right)$. Choosing the $\lambda_{k+}$ eigenvector to be first, this transformation matrix is given by

(67) $$\xi \left(k\right)=\frac{1}{\sqrt{2}}\left[\begin{array}{cc} 1 & 1\\ \frac{{\tilde{V}}_{↑↓}\left(k\right)}{|{\tilde{V}}_{↑↓}\left(k\right)|} & -\frac{{\tilde{V}}_{↑↓}\left(k\right)}{|{\tilde{V}}_{↑↓}\left(k\right)|} \end{array}\right].$$

This diagonalizes the 2×2 matrix $\tilde{V}\left(k\right)$ according to

(68) $$\left[\begin{array}{cc} \lambda_{+}\left(k\right) & 0\\ 0 & \lambda_{-}\left(k\right) \end{array}\right]=\xi^{†}\left(k\right)\tilde{V}\left(k\right)\xi \left(k\right).$$

We can write the combined process of Fourier transforming V into block-diagonal form and then diagonalizing the 2×2 blocks $\tilde{V}\left(k\right)$ as a single unitary transformation M given by

(69) $$M_{\left(\ell ,b),(k,\sigma \right)}=\frac{\xi_{b,\sigma }\left(k\right)}{\sqrt{2N+1}}e^{ik\ell },$$

which completely diagonalizes V such that

(70) $$\Lambda =M^{†}VM.$$

Here, Λ is a diagonal matrix, with each element along the diagonal given by:

(71) $$\Lambda_{\left(k_{1},\sigma_{1}\right),\left(k_{2},\sigma_{2}\right)}=\delta_{k_{1},k_{2}}\delta_{\sigma_{1},\sigma_{2}}\lambda_{k_{1}\sigma_{1}}\;,$$

where σ is either + or −.

Although we have diagonalized the potential with a unitary transformation, the fact that the transformation matrix is complex means that the charge per site in the diagonal basis could also be complex, which is physically undesirable. However, the potential matrix in the position basis is real and symmetric, which means that it should be diagonalizable by a real orthogonal transformation matrix W. In fact, the transformation matrix is not unique, since there is a degeneracy in the eigenvalues, since $\lambda_{k,\sigma }=\lambda_{-k,\sigma }$. This means that we can choose eigenvectors that are real by taking two orthogonal linear combinations of the eigenvectors of the unitary transformation M for *k* and −k. Those two new eigenvectors, which represent the columns (k,σ) for k>0 and k<0 of the new transformation matrix W, are given by

(72) $$W_{\left(\ell ,b),(k,\sigma \right)}=\frac{1}{\sqrt{2}}\left\{M_{\left(\ell ,b),(k,\sigma \right)}+M_{\left(\ell ,b),(-k,\sigma \right)}\right\}\;,\;k>0\;,$$

and

(73) $$W_{\left(\ell ,b),(k,\sigma \right)}=i\frac{1}{\sqrt{2}}\left\{M_{\left(\ell ,b),(k,\sigma \right)}-M_{\left(\ell ,b),(-k,\sigma \right)}\right\}\;,\;k<0\;.\;$$

Since $\xi_{b\sigma }\left(-k\right)=\xi_{b\sigma }^{*}\left(k\right)$, these can be written in terms of real and imaginary parts as

(74) W(ℓ,b),(k,σ)=2(2N+1)Reeikℓξbσ(k),k>0.

and

(75) W(ℓ,b),(k,σ)=−2(2N+1)Imeikℓξbσ(k),k<0.

when k=0, the orthogonal transformation is the same as the unitary transformation, that is

(76) $$W_{\left(\ell ,b),(0,\sigma \right)}=M_{\left(\ell ,b),(0,\sigma \right)}=\frac{\xi_{b,\sigma }\left(0\right)}{\sqrt{2N+1}}\;,$$

where

(77) $$\xi \left(0\right)=\frac{1}{\sqrt{2}}\left[\begin{array}{cc} 1 & 1\\ 1 & -1 \end{array}\right]\;.$$

Here, the columns are labeled by the eigenvalue σ=±, and the rows are labeled by the chain b=−1/2 for the first row and b=1/2 for the second row.

This transformation can be written in a more compact form by introducing the unitary transformation matrix Z, whose elements are defined by

(78) $$Z_{\left(k\sigma \right),\left(k^{\prime }\sigma^{\prime }\right)}=\delta_{\sigma ,\sigma^{\prime }}\left\{\begin{array}{cc} \frac{i}{\sqrt{2}}\left(\delta_{k,k^{\prime }}-\delta_{k,-k^{\prime }}\right) & k<0\\ \delta_{k,k^{\prime }} & k=0\\ \frac{1}{\sqrt{2}}\left(\delta_{k,k^{\prime }}+\delta_{k,-k^{\prime }}\right) & k>0 \end{array}\right.\;\;\;.$$

Then, the orthogonal transformation is

(79) W=MZ.

In deriving the thermodynamic quantities for the interacting lattice gas model, it will be convenient to be able to use this orthogonal transformation to diagonalize the potential.

## 5. Partition Function for Interacting DNA Chains

For the interacting lattice gas, the partition function is similar to the noninteracting one in Equations (5) and (8). However, here the full Hamiltonian H from Equation (39) is used, and then the partition function is given by

(80) $$Z_{G}=\sum_{\mathrm{configurations}}e^{-\beta \left(H-\sum_{\ell ,b}\sum_{\gamma }\mu_{\gamma }n_{\ell ,b}^{\left(\gamma \right)}\right)}\;.$$

The full Hamiltonian, including the interaction term from Equation (42) written in matrix form, is

(81) $$H=\sum_{\ell ,b}\sum_{\gamma }\varepsilon_{\gamma }n_{\ell ,b}^{\left(\gamma \right)}+\frac{1}{2}\rho^{T}V\rho \;.$$

In Section 4, we showed that the orthogonal transformation matrix W diagonalizes V. In order to streamline the calculation, it will be useful to insert the identity matrix, in the form $WW^{T}$, to the left and right of V in the interaction term of the Hamiltonian, which gives

(82) $$H=\sum_{\ell ,b}\sum_{\gamma }\varepsilon_{\gamma }n_{\ell ,b}^{\left(\gamma \right)}+\frac{1}{2}\left(\rho^{T}W\right)\left(W^{T}VW\right)\left(W^{T}\rho \right)\;,$$

where we have grouped factors to show the transformed quantities.

The Hamiltonian, with the interaction term written in the diagonal basis, can then be written as

(83) $$\begin{array}{ccc} H & = & \sum_{\ell ,b}\sum_{\gamma }\varepsilon_{\gamma }n_{\ell ,b}^{\left(\gamma \right)}+\frac{1}{2}{\tilde{\rho }}^{2}\Lambda \\ & = & \sum_{\ell ,b}\sum_{\gamma }\varepsilon_{\gamma }n_{\ell ,b}^{\left(\gamma \right)}+\frac{1}{2}\sum_{k,\sigma }{\left({\tilde{\rho }}_{k,\sigma }\right)}^{2}\lambda_{k,\sigma }\;, \end{array}$$

where $\tilde{\rho }=W^{T}\rho$ is the transformed charge per site. We have not transformed the first term in the Hamiltonian to the diagonal basis because it is more convenient later in the calculation to have it in the position basis. Substituting this form into the partition function and writing the resulting expression as two separate exponentials, we have

(84) $$Z_{G}=\sum_{\mathrm{configurations}}e^{-\beta \;\sum_{\ell ,b}\sum_{\gamma }\left(\varepsilon_{\gamma }-\mu_{\gamma }\right)n_{\ell ,b}^{\left(\gamma \right)}}e^{-\frac{\beta }{2}\sum_{k,\sigma }{\tilde{\rho }}_{k,\sigma }^{2}\lambda_{k,\sigma }}\;.$$

We can write the exponential of the sum in the interaction term as a product of exponentials as

(85) $$Z_{G}=\sum_{\mathrm{configurations}}e^{-\beta \;\sum_{\ell ,b}\sum_{\gamma }\left(\varepsilon_{\gamma }-\mu_{\gamma }\right)n_{\ell ,b}^{\left(\gamma \right)}}\prod_{k,\sigma }e^{-\frac{\beta }{2}{\tilde{\rho }}_{k,\sigma }^{2}\lambda_{k,\sigma }}\;.$$

From this expression, we can see that the mean site occupancy for the interacting lattice gas model can be obtained in the same way as in the noninteracting case (9),

(86) $$\langle n^{\left(\gamma \right)}\rangle =\frac{1}{N_{\mathrm{sites}}\beta Z_{G}}\frac{\partial Z_{G}}{\partial \mu_{\gamma }}\;,$$

by differentiating the partition function with respect to the chemical potential.

In the noninteracting case, since all the information about the configuration was contained in $n_{\ell ,b}^{\left(\gamma \right)}$, we were able to decouple the sum over configurations from the sum over lattice sites because the activity was independent of position, and $n_{\ell ,b}^{\left(\gamma \right)}$ only appeared in its exponent. This is because only linear factors of $n_{\ell ,b}^{\left(\gamma \right)}$ were in the exponent in the partition function. While it is still true that $n_{\ell ,b}^{\left(\gamma \right)}$ contains the configuration dependence, it now appears quadratically in the exponent of the partition function, since ${\tilde{\rho }}_{k,\sigma }$ contains linear factors of the $n_{\ell ,b}^{\left(\gamma \right)}$, and the exponent in the interaction term contains ${\tilde{\rho }}_{k,\sigma }^{2}$. This makes it impossible to decouple the sum over configurations from the sum over lattice sites. However, we can use an integral identity, known as the Hubbard–Stratonovich transformation, to replace the term quadratic in ${\tilde{\rho }}_{k,\sigma }$ in the exponential with a term linear in ${\tilde{\rho }}_{k,\sigma }$, meaning that there will only be linear terms in $n_{\ell ,b}^{\left(\gamma \right)}$. This requires introducing the integral over an auxiliary field ${\tilde{\Delta }}_{k,\sigma }$. Then, the sum over configurations is possible to achieve exactly, although at the cost of having to integrate over the auxiliary fields.

The integral identity, which is a complicated way of writing unity, as is shown in Appendix A, is given by

(87) $$\int \sqrt{\frac{\beta \lambda }{-2\pi }}d\tilde{\Delta }\;e^{\frac{\beta }{2}\lambda {\tilde{\Delta }}^{2}-\beta \lambda \tilde{\Delta }\tilde{\rho }}e^{\frac{\beta }{2}\lambda {\tilde{\rho }}^{2}}=1\;,$$

where the integration path is over the real axis from −∞ to *∞* when λ<0 and over the imaginary axis from −i∞ to i∞ when λ>0. In the previous work by Bishop and McMullen [28], the problem was performed in the position basis, and this integral identity, known as the Hubbard–Stratonovich transformation, was written with the matrix version of the potential in the exponent, as given in Negele and Orland [50]. This form creates a dilemma when there are negative and possibly zero eigenvalues of the potential, and it is difficult to determine the path of integration, since it changes depending on the sign of the eigenvalues. Zero eigenvalues would make the determinant of the matrix in that formula zero, and one then finds that there is a division by zero in the formula. By transforming to the diagonal basis, all these difficulties are avoided, since there is a separate integral for each eigenvalue, and if a particular eigenvalue is zero, the identity is not used at all.

To use the identity in Equation (87), we make the identifications that $\lambda =\lambda_{k,\sigma }$, $\tilde{\rho }={\tilde{\rho }}_{k,\sigma }$, and $\tilde{\Delta }={\tilde{\Delta }}_{k,\sigma }$. Substituting this “1” into the partition function, we have

(88) $$\begin{array}{ccc} Z_{G} & = & \sum_{\mathrm{configurations}}\prod_{k,\sigma }\int \sqrt{\frac{\beta \lambda_{k,\sigma }}{-2\pi }}d{\tilde{\Delta }}_{k,\sigma }e^{\frac{\beta }{2}\lambda_{k,\sigma }{\tilde{\Delta }}_{k,\sigma }^{2}-\beta \lambda_{k,\sigma }{\tilde{\Delta }}_{k,\sigma }{\tilde{\rho }}_{k,\sigma }}e^{\frac{\beta }{2}\lambda_{k,\sigma }{\tilde{\rho }}_{k,\sigma }^{2}}\times \\ & & \times e^{-\beta \sum_{\ell ,b}\sum_{\gamma }\left(\varepsilon_{\gamma }-\mu_{\gamma }\right)n_{\ell ,b}^{\left(\gamma \right)}}e^{-\frac{\beta }{2}{\tilde{\rho }}_{k,\sigma }^{2}\lambda_{k,\sigma }}\;. \end{array}$$

Now, we see that the two exponentials containing ${\tilde{\rho }}_{k,\sigma }^{2}$ cancel, as we planned. Of course, we have gained this convenience by introducing the auxiliary field ${\tilde{\Delta }}_{k,\sigma }$, and we will have to perform the integral over this variable at a later stage. However, since ${\tilde{\Delta }}_{k,\sigma }$ does not depend on the configuration of the system, we can bring the sum over configurations and the leading exponential factors in the partition function inside the integral, and the expression for the partition function becomes

(89) $$\begin{array}{ccc} Z_{G} & = & \int \left(\prod_{k,\sigma }\sqrt{\frac{\beta \lambda_{k,\sigma }}{-2\pi }}d{\tilde{\Delta }}_{k,\sigma }\right)\sum_{\mathrm{configurations}}\exp\left\{-\beta \sum_{\ell ,b}\sum_{\gamma }\left(\varepsilon_{\gamma }-\mu_{\gamma }\right)n_{\ell ,b}^{\left(\gamma \right)}\right.+\\ & & \left.+\sum_{k,\sigma }\left[\frac{\beta }{2}\lambda_{k,\sigma }{\tilde{\Delta }}_{k,\sigma }^{2}-\beta \lambda_{k,\sigma }{\tilde{\rho }}_{k,\sigma }{\tilde{\Delta }}_{k,\sigma }\right]\right\}\;. \end{array}$$

It will be useful to define an action S such that the partition function can be written in the form

(90) $$Z_{G}=\int D\left[\tilde{\Delta }\right]\sum_{\mathrm{configurations}}e^{-S[\tilde{\Delta }]},$$

where

(91) $$S\left[\tilde{\Delta }\right]=\beta \left\{\sum_{\ell ,b}\sum_{\gamma }\left(\varepsilon_{\gamma }-\mu_{\gamma }\right)n_{\ell ,b}^{\left(\gamma \right)}+\sum_{k_{,}\sigma }\left[-\frac{\beta }{2}\lambda_{k,\sigma }{\tilde{\Delta }}_{k,\sigma }^{2}+\beta \lambda_{k,\sigma }{\tilde{\rho }}_{k,\sigma }{\tilde{\Delta }}_{k,\sigma }\right]\right\}$$

and

(92) $$D\left[\tilde{\Delta }\right]=\left(\prod_{k,\sigma }\sqrt{\frac{\beta \lambda_{k,\sigma }}{-2\pi }}d{\tilde{\Delta }}_{k,\sigma }\right)\;.$$

Note that the factors of $\beta \lambda_{k,\sigma }$ are included here in the grand differential $D[\tilde{\Delta }]$, rather than incorporating them in the action $S[\tilde{\Delta }]$, as performed in Bishop and McMullen [28] and in Sievert [33]. When these factors appear in the action, they introduce a term of the form $\ln\sqrt{\det(\beta v)}$. This adds a large constant value to the mean-field results, which is subtracted out when the fluctuation terms are included. It actually has no real physical meaning and is part of the normalization of the integral [51].

We see that by diagonalizing first, our auxiliary fields are in the diagonal basis of the potential. If we transform the terms containing $\varepsilon_{\gamma }$ and $\mu_{\gamma }$ to this basis, they will no longer be diagonal. Therefore, we will leave those terms in the position basis. It will also be convenient to have the term containing $\sum_{k_{,}\sigma }\lambda_{k,\sigma }{\tilde{\rho }}_{k,\sigma }{\tilde{\Delta }}_{k,\sigma }$ in the position basis also. Therefore, the expression for the transformed charge per site is

(93) $$\begin{array}{ccc} {\tilde{\rho }}_{k,\sigma } & = & \sum_{\ell ,b}W_{\left(k,\sigma ),(\ell ,b\right)}^{T}\sum_{\gamma }\rho_{\ell ,b}^{\left(\gamma \right)}\\ & = & \sum_{\ell ,b}W_{\left(k,\sigma ),(\ell ,b\right)}^{T}\sum_{\gamma }q_{\gamma }n_{\left(\ell ,b\right)}^{\left(\gamma \right)}\;, \end{array}$$

in terms of the quantities in real space.

Substituting this into the action, we have

(94) $$S\left[\tilde{\Delta }\right]=\beta \left\{\sum_{\ell ,b}\sum_{\gamma }\left(\varepsilon_{\gamma }-\mu_{\gamma }\right)n_{\left(\ell ,b\right)}^{\left(\gamma \right)}-\sum_{k_{,}\sigma }\left[\frac{1}{2}\lambda_{k,\sigma }{\tilde{\Delta }}_{k,\sigma }^{2}+{\tilde{\Delta }}_{k,\sigma }\lambda_{k,\sigma }\sum_{\ell ,b}W_{\left(k,\sigma ),(\ell ,b\right)}^{T}\sum_{\gamma }q_{\gamma }n_{\left(\ell ,b\right)}^{\left(\gamma \right)}\right]\right\}\;.$$

It is convenient to identify the last term in this expression as a self-energy of species γ,

(95) $${\tilde{\Sigma }}_{\ell ,b}^{\left(\gamma \right)}\left[\tilde{\Delta }\right]=\left(\sum_{k,\sigma }{\tilde{\Delta }}_{k,\sigma }\lambda_{k,\sigma }W_{\left(k,\sigma ),(\ell ,b\right)}^{T}\right)q_{\gamma }\;.$$

By transforming ${\tilde{\Delta }}_{k,\sigma }$ to the position basis as

(96) $${\tilde{\Delta }}_{k,\sigma }=\sum_{\ell^{\prime },b^{\prime }}\Delta_{\ell^{\prime },b^{\prime }}W_{\left(\ell^{\prime },b^{\prime }\right),\left(k,\sigma \right)}\;,$$

this self-energy can also be written in the position basis as

(97) $$\begin{array}{ccc} \Sigma_{\ell ,b}^{\left(\gamma \right)}\left[\Delta \right] & = & {\tilde{\Sigma }}_{\ell ,b}^{\left(\gamma \right)}\left[\tilde{\Delta }\right]\\ & = & q_{\gamma }\sum_{\ell^{\prime },b^{\prime }}\Delta_{\ell^{\prime },b^{\prime }}\left(\sum_{k,\sigma }W_{\left(\ell^{\prime },b^{\prime }\right),\left(k,\sigma \right)}\lambda_{k,\sigma }W_{\left(k,\sigma ),(\ell ,b\right)}^{T}\right)\\ & = & q_{\gamma }\sum_{\ell^{\prime },b^{\prime }}\Delta_{\ell^{\prime },b^{\prime }}V_{\left(\ell^{\prime },b^{\prime }\right),\left(\ell ,b\right)}\;. \end{array}$$

This will be a useful form to use when we discuss the saddle-point, or mean-field, approximation.

Keeping the first term in the position basis and the second term in the diagonal basis, we now write the action in a compact form, combining the self-energy term with the first term in the action to obtain

(98) $$S\left[\tilde{\Delta }\right]=\beta \left\{\sum_{\ell ,b}\sum_{\gamma }\left(\varepsilon_{\gamma }+\Sigma_{\ell ,b}^{\left(\gamma \right)}\left[\Delta \right]-\mu_{\gamma }\right)n_{\left(\ell ,b\right)}^{\left(\gamma \right)}-\sum_{k_{,}\sigma }\left[\frac{1}{2}\lambda_{k,\sigma }{\tilde{\Delta }}_{k,\sigma }^{2}\right]\right\}\;.$$

Analogous to the noninteracting case, we define the activity as

(99) $${\tilde{a}}_{\ell ,b}^{\left(\gamma \right)}\left[\tilde{\Delta }\right]=a_{\ell ,b}^{\left(\gamma \right)}\left[\Delta \right]=e^{-\beta (\varepsilon_{\gamma }+\Sigma_{\ell ,b}^{\left(\gamma \right)}\left[\Delta \right]-\mu_{\gamma })}\;.$$

With this definition, the partition function becomes

(100) $$Z_{G}=\int D\left[\tilde{\Delta }\right]\sum_{\mathrm{configurations}}\left(\prod_{\ell ,b}\prod_{\gamma }{\left\{a_{\ell ,b}^{\left(\gamma \right)}\left[\Delta \right]\right\}}^{n_{\left(\ell ,b\right)}^{\left(\gamma \right)}}\right)e^{\beta \sum_{k_{,}\sigma }\left[\frac{1}{2}\lambda_{k,\sigma }{\tilde{\Delta }}_{k,\sigma }^{2}\right]}\;.$$

The trace over configurations is now performed over each site separately, exactly as we did for the noninteracting case, where $n_{\left(\ell ,b\right)}^{\left(\gamma \right)}=1$ for one and only one of γ=‖,⊥, or *v*, and zero otherwise. Thus, performing the trace gives

(101) $$\sum_{\begin{array}{c} \mathrm{single-site}\\ \mathrm{configurations} \end{array}}\prod_{\gamma }{\left\{{\tilde{a}}_{\ell ,b}^{\left(\gamma \right)}\left[\tilde{\Delta }\right]\right\}}^{n_{\left(\ell ,b\right)}^{\left(\gamma \right)}}=\sum_{\gamma }{\tilde{a}}_{\ell ,b}^{\left(\gamma \right)}\left[\tilde{\Delta }\right]\;\;,$$

and the grand partition function becomes

(102) $$Z_{G}=\int D\left[\tilde{\Delta }\right]e^{-S_{\mathrm{eff}}\left[\tilde{\Delta }\right]},$$

where the effective action is

(103) $$S_{\mathrm{eff}}\left[\tilde{\Delta }\right]=-\frac{\beta }{2}\sum_{k_{,}\sigma }\lambda_{k,\sigma }{\tilde{\Delta }}_{k,\sigma }^{2}-\sum_{\ell ,b}\ln\left(\sum_{\gamma }{\tilde{a}}_{\ell ,b}^{\left(\gamma \right)}\left[\tilde{\Delta }\right]\right)\;.$$

This is the general result, which is in principle exact. To understand what the Hubbard–Stratonovich transformation has accomplished for us, recall that the interaction energy $U_{\mathrm{int}}$ posed two difficulties: the additional configuration dependence and the coupling between the sites. By introducing auxiliary fields through the Hubbard–Stratonovich transformation (87), we managed to separate these two complications so that one factor $\exp\left[-\frac{\beta }{2}\sum_{k_{,}\sigma }\lambda_{k,\sigma }{\tilde{\Delta }}_{k,\sigma }^{2}\right]$ contains interactions between field fluctuations but no configuration dependence, and another factor $\exp\left[\beta \sum_{k,\sigma }\lambda_{k,\sigma }{\tilde{\rho }}_{k,\sigma }{\tilde{\Delta }}_{k,\sigma }\right]$ is configuration-dependent (through ${\tilde{\rho }}_{k,\sigma }$) but decoupled. This allowed us to define a modified activity (99) that incorporated all the configuration dependence, making it possible to evaluate the sum over configurations directly, as in the noninteracting case.

What we have achieved in using the Hubbard–Stratonovich transformation is to replace the interaction part of the partition function with its functional integral representation. From the form of Equation (100), we see that the largest contribution to the partition function comes from the values of ${\tilde{\Delta }}_{k,\sigma }^{2}$ for which the effective action $S_{\mathrm{eff}}$ is stationary, i.e., at a saddle point. Thus, a Taylor series expansion of the effective action (103) about the saddle point will yield an order-by-order approximation to the exact partition function (102).

## 6. Expansion in Powers of the Auxiliary Field

Since it is not possible to evaluate the integral in the partition function of Equation (102) exactly, we will approximate it by expanding the action in Equation (103) about a mean field $\Delta_{c}$, and include fluctuations to the lowest order about this mean field. This mean field is determined by the saddle point of the integrand in Equation (102), which is the largest contribution to the integral.

We can write the effective action, then, as an expansion about this uniform saddle point. As an aid to keeping track of the order of the expansions, we add a multiplicative factor *m* in front of the effective action:

(104) $$\begin{array}{ccc} mS_{\mathrm{eff}}\left(\tilde{\Delta }={\tilde{\Delta }}_{c}+\delta \tilde{\Delta }/\sqrt{m}\right) & = & mS_{\mathrm{eff}}\left[{\tilde{\Delta }}_{c}\right]+{\left.\sum_{k,\sigma }\frac{\delta {\tilde{\Delta }}_{k,\sigma }}{\sqrt{m}}m\frac{\partial S_{\mathrm{eff}}\left[\tilde{\Delta }\right]}{\partial {\tilde{\Delta }}_{k,\sigma }}\right|}_{{\tilde{\Delta }}_{c}}+\\ & + & {\left.\frac{1}{2}\sum_{k_{1},\sigma_{1},k_{2},\sigma_{2}}\frac{\delta {\tilde{\Delta }}_{k_{1},\sigma_{1}}}{\sqrt{m}}m\frac{\partial^{2}S_{\mathrm{eff}}\left[\tilde{\Delta }\right]}{\partial {\tilde{\Delta }}_{k_{1},\sigma_{1}}\partial {\tilde{\Delta }}_{k_{2},\sigma_{2}}}\right|}_{{\tilde{\Delta }}_{c}}\frac{\delta {\tilde{\Delta }}_{k_{2},\sigma_{2}}}{\sqrt{m}}+\ldots \;. \end{array}$$

Here, the expansion parameter $\delta \tilde{\Delta }$ is scaled by $m^{-1/2}$ by writing $\delta {\tilde{\Delta }}_{k,\sigma }\equiv \sqrt{m}\left[{\tilde{\Delta }}_{k,\sigma }-{\tilde{\Delta }}_{k,\sigma ,c}\right]$. The factors of *m* cancel in the quadratic term, so that it is of order $m^{0}$.

In evaluating the integral in the partition function, the largest contribution comes from the region near a saddle point, and the terms beyond the saddle point give corrections to the integral. Physically, the saddle-point solution corresponds to a mean-field approximation, and we begin with that. The saddle point is found by setting the first derivative in the expansion to zero, that is

(105) $${\left.\frac{\partial S_{\mathrm{eff}}\left[\tilde{\Delta }\right]}{\partial {\tilde{\Delta }}_{k,\sigma }}\right|}_{{\tilde{\Delta }}_{c}}=0\;.$$

The second derivatives evaluated at the saddle point are proportional to the components of a matrix ${\tilde{D}}^{-1}$, which is the inverse propagator for the fluctuations in the auxiliary Hartree field Δ that we introduced to decouple the interparticle interactions. Because we want to absorb the normalization factor in the grand differential $D[\tilde{\Delta }]$, we have defined each of the elements of ${\tilde{D}}^{-1}$ by the second derivative of $S_{\mathrm{eff}}\left[\tilde{\Delta }\right]$ divided by $-\beta \lambda_{k,\sigma }$ as

(106) $${\left({\tilde{D}}^{-1}\right)}_{\left(k_{1},\sigma_{1}\right),\left(k_{2},\sigma_{2}\right)}=-\left(\frac{1}{\beta \lambda_{k,\sigma }}\right){\left.\frac{\partial^{2}S_{\mathrm{eff}}\left[\tilde{\Delta }\right]}{\partial {\tilde{\Delta }}_{k_{1},\sigma_{1}}\partial {\tilde{\Delta }}_{k_{2},\sigma_{2}}}\right|}_{{\tilde{\Delta }}_{c}}.$$

We will show in Section 8 that, for a spatially uniform saddle point, this matrix is diagonal, that is, $S_{\mathrm{eff}}\left[\tilde{\Delta }\right]$ is diagonal in the $\delta {\tilde{\Delta }}_{k_{1},\sigma_{1}}$’s, that is,

(107) $${\left({\tilde{D}}^{-1}\right)}_{\left(k_{1},\sigma_{1}\right),\left(k_{2},\sigma_{2}\right)}={\left({\tilde{D}}^{-1}\right)}_{\left(k_{1},\sigma_{1}\right),\left(k_{1},\sigma_{1}\right)}\delta_{k_{1},k_{2}}\delta_{\sigma_{1},\sigma_{2}}\;.$$

Although the Gaussian integral can still be performed if ${\tilde{D}}^{-1}$ is not diagonal, the argument becomes much easier to follow if we assume at this point that ${\tilde{D}}^{-1}$ is diagonal, because then the Gaussian integral can be performed straightforwardly.

With this assumption that ${\tilde{D}}^{-1}$ is diagonal, the expansion of the effective action reduces to

(108) $$mS_{\mathrm{eff}}\left(\tilde{\Delta }={\tilde{\Delta }}_{c}+\delta \tilde{\Delta }/\sqrt{m}\right)≃mS_{\mathrm{eff}}\left[{\tilde{\Delta }}_{c}\right]-\frac{1}{2}\sum_{k,\sigma }\beta \lambda_{k,\sigma }{\left({\tilde{D}}^{-1}\right)}_{\left(k,\sigma ),(k,\sigma \right)}{\left(\delta {\tilde{\Delta }}_{k,\sigma }\right)}^{2}\;.$$

The grand canonical partition function can then be written as

(109) $$Z_{G}≃e^{-mS_{\mathrm{eff}}\left[{\tilde{\Delta }}_{c}\right]}\int \left(\prod_{k,\sigma }\sqrt{\frac{\beta \lambda_{k,\sigma }}{-2\pi }}d\left(\delta {\tilde{\Delta }}_{k,\sigma }\right)\right)e^{\frac{1}{2}\beta \sum_{k,\sigma }\lambda_{k,\sigma }{\left({\tilde{D}}^{-1}\right)}_{\left(k,\sigma ),(k,\sigma \right)}{\left(\delta {\tilde{\Delta }}_{k,\sigma }\right)}^{2}}\;.$$

This can be written as a product of Gaussian integrals as

(110) $$Z_{G}≃e^{-mS_{\mathrm{eff}}\left[{\tilde{\Delta }}_{c}\right]}\prod_{k,\sigma }\int \left(\sqrt{\frac{\beta \lambda_{k,\sigma }}{-2\pi }}d\left(\delta {\tilde{\Delta }}_{k,\sigma }\right)\right)e^{\frac{1}{2}\beta \lambda_{k,\sigma }{\left({\tilde{D}}^{-1}\right)}_{\left(k,\sigma ),(k,\sigma \right)}{\left(\delta {\tilde{\Delta }}_{k,\sigma }\right)}^{2}}\;,$$

and each Gaussian integral can be evaluated using the same change in variable and path of integration as was used in the Hubbard–Stratonovich transformation in Equation (87), that originally was used to define the auxiliary fields, and is discussed in Appendix A. Each Gaussian integral produces a result of the same form, regardless of whether ${\left({\tilde{D}}^{-1}\right)}_{\left(k,\sigma ),(k,\sigma \right)}$ is positive or negative. This quantity can never be zero, since we would not have defined an auxiliary field for that case, which is the same as the case in which an eigenvalue of the potential is zero. Therefore, the integral can always be evaluated, and the partition function can be written as

(111) $$\begin{array}{ccc} Z_{G} & ≃ & e^{-mS_{\mathrm{eff}}\left[{\tilde{\Delta }}_{c}\right]}\prod_{k,\sigma }\frac{1}{\sqrt{{\left({\tilde{D}}^{-1}\right)}_{\left(k,\sigma ),(k,\sigma \right)}}}\;. \end{array}$$

Note that all the factors of $\beta \lambda_{k,\sigma }$ have canceled out due to our choices of the form of the effective action $S_{\mathrm{eff}}$ and the inverse Hartree-field-fluctuation propagator ${\tilde{D}}^{-1}$. Choosing these quantities carefully allows for the correct normalization of the integral [51]. The product can now be brought inside the square root, and since ${\tilde{D}}^{-1}$ is diagonal, this gives the determinant of the matrix inside the square root, and so the partition function assumes the form

(112) $$\begin{array}{ccc} Z_{G} & ≃ & e^{-mS_{\mathrm{eff}}\left[{\tilde{\Delta }}_{c}\right]}\frac{1}{\sqrt{\det({\tilde{D}}^{-1})}}\;, \end{array}$$

and the partition function can be written as a single exponential,

(113) $$\begin{array}{ccc} Z_{G} & ≃ & e^{-\{mS_{\mathrm{eff}}\left[{\tilde{\Delta }}_{c}\right]+\frac{1}{2}\ln\det\left({\tilde{D}}^{-1}\right)\}}\;. \end{array}$$

The grand canonical potential is then

(114) $$\Omega_{G}=-\frac{1}{\beta }\ln Z_{G}≃\frac{1}{\beta }\left\{mS_{\mathrm{eff}}\left[{\tilde{\Delta }}_{c}\right]+\frac{1}{2}\ln\det\left({\tilde{D}}^{-1}\right)\right\}\;.$$

If we formally write the expansion in terms of orders in *m*, we have

(115) $$\Omega_{G}≃m\Omega_{m=1}+\Omega_{m=0}+\frac{1}{\sqrt{m}}\Omega_{m=-\frac{1}{2}}+\frac{1}{m}\Omega_{m=-1}+\cdots \;.$$

The leading term in the expansion is, therefore, given by the saddle-point value

(116) $$m\Omega_{m=1}=m\Omega_{c}=\frac{m}{\beta }S_{\mathrm{eff}}\left[{\tilde{\Delta }}_{c}\right]\;,$$

and what is known as the “one-loop” correction term is

(117) $$\Omega_{m=0}=\frac{1}{2}\ln\det\left({\tilde{D}}^{-1}\right)=\frac{1}{2}\mathrm{Tr}\ln{\tilde{D}}^{-1}.$$

## 7. The Saddle-Point, or Mean-Field, Approximation

The first task in this section is to find an equation that determines the value of the auxiliary field at the saddle-point, or mean-field, value of the effective action. In order to find this saddle point, we must set the first derivative of the effective action in Equation (103) with respect to the auxiliary field ${\tilde{\Delta }}_{k,\sigma }$ to zero. That derivative is given by

(118) $$\frac{\partial S_{\mathrm{eff}}\left[\tilde{\Delta }\right]}{\partial {\tilde{\Delta }}_{k,\sigma }}=-\beta \lambda_{k,\sigma }{\tilde{\Delta }}_{k,\sigma }-\beta \sum_{\ell ,b}\frac{1}{\left(\sum_{\gamma^{\prime }}{\tilde{a}}_{\ell ,b}^{\left(\gamma^{\prime }\right)}\left[\tilde{\Delta }\right]\right)}\sum_{\gamma }\frac{\partial {\tilde{a}}_{\ell ,b}^{\left(\gamma \right)}\left[\tilde{\Delta }\right]}{\partial {\tilde{\Delta }}_{k,\sigma }}\;.$$

The activity $a_{\ell ,b}^{\left(\gamma \right)}\left[\tilde{\Delta }\right]$ for species γ is given by Equation (99), and its derivative is

(119) $$\frac{\partial {\tilde{a}}_{\ell ,b}^{\left(\gamma \right)}\left[\tilde{\Delta }\right]}{\partial {\tilde{\Delta }}_{k,\sigma }}=-\beta {\tilde{a}}_{\ell ,b}^{\left(\gamma \right)}\left[\tilde{\Delta }\right]\frac{\partial {\tilde{\Sigma }}_{\ell ,b}^{\left(\gamma \right)}\left[\tilde{\Delta }\right]}{\partial {\tilde{\Delta }}_{k,\sigma }}\;,$$

where from Equation (95), the derivative of the self-energy is

(120) $$\frac{\partial {\tilde{\Sigma }}_{\ell ,b}^{\left(\gamma \right)}\left[\tilde{\Delta }\right]}{\partial {\tilde{\Delta }}_{k,\sigma }}=q_{\gamma }\lambda_{k,\sigma }W_{\left(k,\sigma ),(\ell ,b\right)}^{T}\;.$$

Substituting this into the derivative of the action, we have

(121) $$\frac{\partial S_{\mathrm{eff}}\left[\tilde{\Delta }\right]}{\partial {\tilde{\Delta }}_{k,\sigma }}=-\beta \lambda_{k,\sigma }{\tilde{\Delta }}_{k,\sigma }+\beta \lambda_{k,\sigma }\sum_{\ell ,b}W_{\left(k,\sigma ),(\ell ,b\right)}^{T}\frac{\sum_{\gamma }q_{\gamma }{\tilde{a}}_{\ell ,b}^{\left(\gamma \right)}\left[\tilde{\Delta }\right]}{\sum_{\gamma^{\prime }}{\tilde{a}}_{\ell ,b}^{\left(\gamma^{\prime }\right)}\left[\tilde{\Delta }\right]}\;.$$

Setting this first derivative to zero, we obtain an equation whose solution gives the auxiliary field in the saddle-point approximation,

(122) $${\tilde{\Delta }}_{\left(k,\sigma \right)}=\sum_{\ell ,b}W_{\left(k,\sigma ),(\ell ,b\right)}^{T}\left(\frac{\sum_{\gamma }\;q_{\gamma }{\tilde{a}}_{\ell ,b}^{\left(\gamma \right)}\left[\tilde{\Delta }\right]}{\sum_{\gamma^{\prime }}{\tilde{a}}_{\ell ,b}^{\left(\gamma^{\prime }\right)}\left[\tilde{\Delta }\right]}\right)\;.$$

We see that on the right-hand side of the equation is the transformation to the diagonal basis of the potential, as given in Equation (96). We could, therefore, write the auxiliary field in the position basis as

(123) $$\Delta_{\left(\ell ,b\right)}=\frac{\sum_{\gamma }q_{\gamma }a_{\ell ,b}^{\left(\gamma \right)}\left[\Delta \right]}{\sum_{\gamma^{\prime }}a_{\ell ,b}^{\left(\gamma^{\prime }\right)}\left[\Delta \right]}\;,$$

where $a_{\ell ,b}^{\left(\gamma \right)}\left[\Delta \right]$ is the activity with the self-energy in the diagonal basis ${\tilde{\Sigma }}_{\ell ,b}^{\left(\gamma \right)}\left[\tilde{\Delta }\right]$, which by Equation (97) has been replaced by the self-energy in the position basis, $\Sigma_{\ell ,b}^{\left(\gamma \right)}\left[\Delta \right]$. Either of these two equations, Equation (122) or (123), may be considered the equation giving the saddle-point, or mean-field, value of the auxiliary field, and we will refer to this as the mean-field equation. In this expression, $a_{\ell ,b}^{\left(\gamma \right)}\left[\Delta_{c}\right]$ is a function of all the $\Delta_{(\ell ,b),c}$ elements, and so, for N=30, which we have used in our calculations, there are $N_{\mathrm{sites}}=2\left(2N+1\right)=122$ coupled nonlinear equations. Therefore, for simplicity, we make the assumption that the mean field is spatially uniform, which means that

(124) $$\Delta_{(\ell ,b),c}=\Delta_{c}\;,$$

where $\Delta_{c}$ is constant. This is still a nonlinear equation in $\Delta_{c}$, and so this must be solved numerically with an iterative approach.

Before solving the mean-field equation, it is useful to have a physical interpretation of the mean auxiliary field $\Delta_{c}$. The first step in this direction is the second task of this section, which is to find the mean site occupancies of species γ at the mean-field level. This is achieved as in the noninteracting case, by taking the derivative of the partition function with respect to $\mu_{\gamma }$. At this mean-field level, the $\mu_{\gamma }$ dependence is contained in $S_{\mathrm{eff}}\left[{\tilde{\Delta }}_{c}\right]$ in Equation (113), neglecting the term containing the inverse propagator $D^{-1}$. The expression for the mean occupancy can be obtained using Equation (86) as

(125) $${\langle n^{\left(\gamma \right)}\rangle }_{c}=\frac{1}{N_{\mathrm{sites}}\beta }\frac{\partial }{\partial \mu_{\gamma }}\ln\left[e^{-S_{\mathrm{eff}}\left[{\tilde{\Delta }}_{c}\right]}\right]\;.$$

Taking the derivative of the exponential, we can cancel out the partition function in the denominator and we are left with the derivative of the effective action as

(126) $${\langle n^{\left(\gamma \right)}\rangle }_{c}=-\frac{1}{N_{\mathrm{sites}}\beta }\frac{\partial }{\partial \mu_{\gamma }}S_{\mathrm{eff}}\left[{\tilde{\Delta }}_{c}\right]\;.$$

Substituting the effective action from Equation (103), we have

(127) $${\langle n^{\left(\gamma \right)}\rangle }_{c}=\frac{1}{N_{\mathrm{sites}}\beta }\sum_{\ell ,b}\frac{1}{\left(\sum_{\gamma^{\prime }}a_{\ell ,b}^{\left(\gamma^{\prime }\right)}\left[\Delta_{c}\right]\right)}\frac{\partial a_{\ell ,b}^{\left(\gamma \right)}\left[\Delta_{c}\right]}{\partial \mu_{\gamma }}\;.$$

The derivative of the activity is

(128) $$\frac{\partial a_{\ell ,b}^{\left(\gamma \right)}\left[\Delta_{c}\right]}{\partial \mu_{\gamma }}=\beta a_{\ell ,b}^{\left(\gamma \right)}\left[\Delta_{c}\right]\;,$$

and so the mean occupancy becomes

(129) $${\langle n^{\left(\gamma \right)}\rangle }_{c}=\frac{1}{N_{\mathrm{sites}}}\sum_{\ell ,b}\frac{a_{\ell ,b}^{\left(\gamma \right)}\left[\Delta_{c}\right]}{\left(\sum_{\gamma^{\prime }}a_{\ell ,b}^{\left(\gamma^{\prime }\right)}\left[\Delta_{c}\right]\right)}\;.$$

The third task of this section is to relate the mean charge per site to the mean occupancy. The charge per site has the same form as in Equation (22) for the noninteracting model, so that at the mean-field level we obtain

(130) $$\rho_{c}={\langle \rho \rangle }_{c}=\sum_{\gamma }q_{\gamma }{\langle n^{\left(\gamma \right)}\rangle }_{c}=\frac{\sum_{\gamma }q_{\gamma }a_{\ell ,b}^{\left(\gamma \right)}\left[\Delta_{c}\right]}{\sum_{\gamma^{\prime }}a_{\ell ,b}^{\left(\gamma^{\prime }\right)}\left[\Delta_{c}\right]}\;,$$

which, like the mean site occupancy, is independent of the site index because of our choice of spatially uniform mean field. This is equivalent to the equation for the saddle-point value of the auxiliary field because the right-hand side of the equation shows that

(131) $$\rho_{c}=\Delta_{c}\;,$$

on comparison with Equation (123).

In the mean-field, or saddle-point, equation for the spatially uniform saddle point, the auxiliary field is the same for all lattice sites, and the self-energy becomes spatially uniform also and can be written as

(132) $$\Sigma_{\ell ,b}^{\left(\gamma \right)}\left[\Delta_{c}\right]\equiv \Sigma_{c}^{\left(\gamma \right)}=\rho_{c}q_{\gamma }S_{\mathrm{lattice}}\;.$$

where $S_{\mathrm{lattice}}$ is a lattice sum that is independent of the choice of lattice site (ℓ,b) for a long DNA double helix, and is given by

(133) $$S_{\mathrm{lattice}}=\sum_{\ell^{\prime },b^{\prime }}V_{\left(\ell^{\prime },b^{\prime }\right),\left(\ell ,b\right)}\;.$$

For the parameters used in the figures, $\beta S_{\mathrm{lattice}}\approx 2.71$.

Consequently, at the mean-field level, the self-energy simply represents the electrostatic energy of charge $q_{\gamma }$ interacting with the rest of the DNA lattice. This term plays the role of the self-energy Σ in many-body quantum mechanics [28,50,51]. Since the self-energy is independent of the lattice site, so is the activity, which can be written as

(134) $$a_{c}^{\left(\gamma \right)}=a_{\ell ,b}^{\left(\gamma \right)}\left[\Delta_{c}\right]=e^{-\beta (\varepsilon_{\gamma }-\mu_{\gamma }+\Sigma_{c}^{\left(\gamma \right)})}\;.$$

The simplifications made possible by the assumption of a spatially uniform saddle point allow us to rewrite the mean-field equation given by Equation (123) in an explicit form as

(135) $$\rho_{c}=\frac{\sum_{\gamma }q_{\gamma }e^{-\beta \left(\varepsilon_{\gamma }-\mu_{\gamma }+q_{\gamma }\rho_{c}S_{\mathrm{lattice}}\right)}}{\sum_{\gamma }e^{-\beta \left(\varepsilon_{\gamma }-\mu_{\gamma }+q_{\gamma }\rho_{c}S_{\mathrm{lattice}}\right)}}\;.$$

Again, recall that $\varepsilon_{v}-\mu_{v}=0$, identically, $q_{\|}=0$, and $\mu_{\|}=\mu_{⊥}=\mu_{\mathrm{dimer}}$. Therefore, when $\rho_{c}=0$, we can see from Equation (135) that $q_{⊥}e^{-\beta (\varepsilon_{⊥}-\mu_{\mathrm{dimer}})}+q_{v}=0$, and since $q_{⊥}=+1$, and $q_{v}=-1$, it follows that $\varepsilon_{⊥}=\mu_{\mathrm{dimer}}$. Therefore, this is the point at which the charge per site $\rho_{c}$ goes to zero. When $\varepsilon_{\|}=2\varepsilon_{⊥}$, the charge per site goes to zero at $\varepsilon_{\|}=2\mu_{\mathrm{dimer}}$, which is the same place it went to zero for the noninteracting model, as shown in Figure 6.

Returning to the mean site occupancy in Equation (129), the quantity inside the sum over *ℓ* and *b* is constant and the sum yields $N_{\mathrm{sites}}$, and so the average occupancy per species becomes

(136) $${\langle n^{\left(\gamma \right)}\rangle }_{c}=\frac{a_{c}^{\left(\gamma \right)}}{\left(\sum_{\gamma^{\prime }}a_{c}^{\left(\gamma^{\prime }\right)}\right)}\;.$$

**Figure 15:**
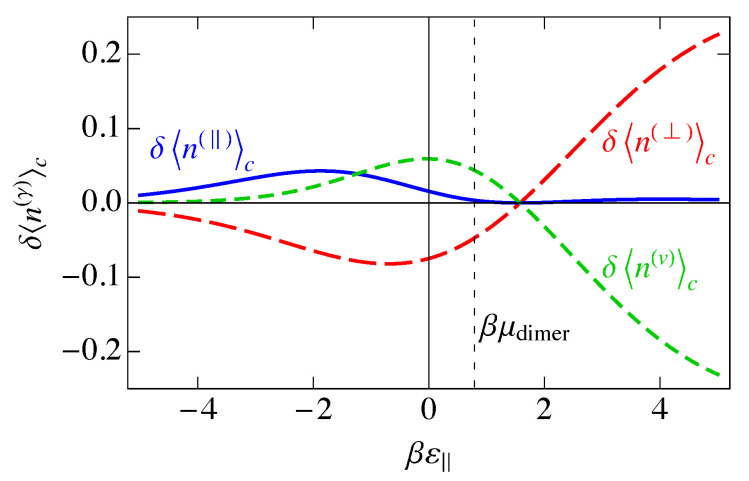
A plot of the differences $\delta {\langle n^{\left(\gamma \right)}\rangle }_{c}$ in mean-field average occupation numbers from their noninteracting values for ‖ (blue, solid),γ=⊥ (red, long-dashed), and *v* (green, short-dashed). The parameters used in the calculation are $\beta \mu_{\mathrm{dimer}}=0.79$ and $\varepsilon_{\|}=2\varepsilon_{⊥}$. The noninteracting results are shown in Figure 5.

**Figure 16:**
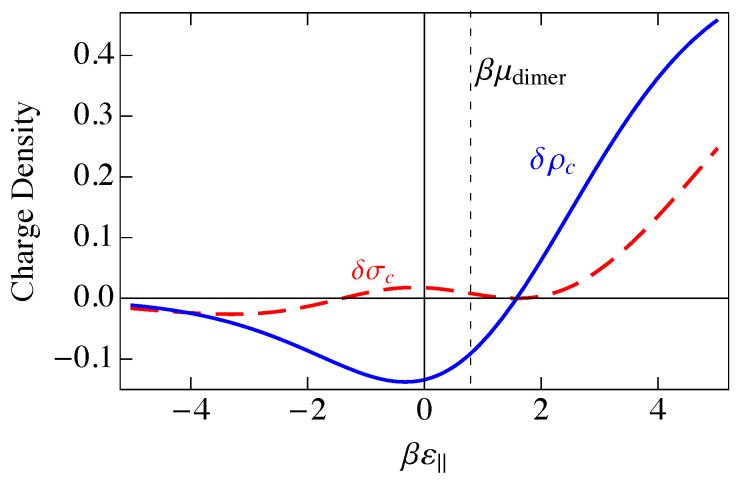
Plots of the differences in the charge per site and standard deviations of the charge per site for the mean field of the interacting lattice gas model and the noninteracting model. $\delta \rho_{c}=\rho_{c}-\rho_{\mathrm{NI}}$ is shown as the solid blue curve, and $\delta \sigma_{c}=\sigma_{c}-\sigma_{\mathrm{NI}}$ is shown as the dashed red curve. The parameters used in the calculation are $\beta \mu_{\mathrm{dimer}}=0.79$ and $\varepsilon_{\|}=2\varepsilon_{⊥}$. The noninteracting results are shown in Figure 6.

Using the mean-field value $\rho_{c}=\Delta_{c}$ of the charge per site, calculated from Equation (135), we have calculated the average occupation numbers $\langle n^{\left(\gamma \right)}\rangle$ via Equation (136) using Equation (134) for the activities. In Figure 15, we have plotted $\delta {\langle n^{\left(\gamma \right)}\rangle }_{c}={\langle n^{\left(\gamma \right)}\rangle }_{c}-{\langle n^{\left(\gamma \right)}\rangle }_{\mathrm{NI}}$, which are the differences in the mean-field occupancies from the occupancies in the noninteracting case shown in Figure 5. The corresponding change in the charge per site $\delta \rho_{c}=\rho_{c}-\rho_{\mathrm{NI}}$ is shown as the solid blue curve in Figure 16.

The shifts in occupancy plotted in Figure 15 can be primarily understood as a consequence of the electromagnetic “self-energy” $\Sigma_{c}^{\left(\gamma \right)}=\rho_{c}q_{\gamma }S_{\mathrm{lattice}}$ of each species γ interacting with the total charge $\rho_{c}$ of the lattice. Immediately, this implies that the parallel dimers are hardly modified at all, since they are electrically neutral and not directly affected by the total charge $\rho_{c}$ of the lattice. For the vacancies and perpendicular dimers, which are charged, the mean-field corrections plotted in Figure 15 and Figure 16 act to reduce the overall charge of the lattice. Below $\beta \varepsilon_{\|}\approx 2$, the total charge of the lattice is positive, so the mean-field corrections penalize the positively charged perpendicular dimers and enhance the negatively charged vacancies. Above $\beta \varepsilon_{\|}\approx 2$, the total charge of the lattice is negative, and this effect is reversed. Thus, the mean-field corrections tend to reduce (but not eliminate) the charge inversion in the weak binding region, $\beta \varepsilon_{\|}>2$, seen in Figure 5 for the non-interacting model. Note that the biggest effect of this electrostatic self-energy is to heavily penalize the “naked lattice” of negatively charged vacancies, which was the favored ground state at large positive $\varepsilon_{\|}$ for the noninteracting Hamiltonian.

The fourth task of this section is to determine the variance in the charge per site from the mean field, similar to Equation (24) for the noninteracting model, which gives a measure of the importance of spatial fluctuations in the charge per site, and can be written in the form

(137) $$\sigma_{c}^{2}={\langle \rho^{2}\rangle }_{c}-\rho_{c}^{2}\;,$$

where the average ${\langle \rho^{2}\rangle }_{c}$ of the square of the charge per site, similar to Equation (29) for the noninteracting model, is

(138) $${\langle \rho^{2}\rangle }_{c}=\sum_{\gamma }q_{\gamma }^{2}{\langle n^{\left(\gamma \right)}\rangle }_{c}=\frac{\sum_{\gamma }q_{\gamma }^{2}a_{c}^{\left(\gamma \right)}}{\sum_{\gamma^{\prime }}a_{c}^{\left(\gamma^{\prime }\right)}}\;.$$

The variance $\sigma_{c}^{2}$ will be useful for knowing the size of the charge fluctuations about the mean field, and this quantity will appear in the next level of approximation. The difference in the standard deviation $\delta \sigma_{c}=\sigma_{c}-\sigma_{\mathrm{NI}}$ is plotted as the red dashed curve in Figure 16, where $\sigma_{\mathrm{NI}}$ for the noninteracting model is plotted as the red dashed curve in Figure 6.

Using a spatially uniform saddle point, which yields a charge per site $\rho_{c}$ that is equal at the mean-field level to the auxiliary field $\Delta_{c}$ in real space, it is interesting to write down the auxiliary field in the diagonal basis, given by

(139) $${\tilde{\Delta }}_{(k,\sigma ),c}=\sum_{\ell ,b}W_{\left(k,\sigma ),(\ell ,b\right)}^{T}{\left.\langle \rho_{\ell ,b}\rangle \right|}_{c}=\rho_{c}\sum_{\ell ,b}W_{\left(k,\sigma ),(\ell ,b\right)}^{T}\;.$$

From Equations (74) and (75), we see that when k≠0, $W_{\left(k,\sigma ),(\ell ,b\right)}^{T}$ has the *ℓ* dependence of the form $e^{\pm ik\ell }$, which by Equation (59) sums to zero. The only surviving term is for k=0, which when summed over *ℓ* gives 2N+1, and we have from Equations (76) and (139)

(140) $${\tilde{\Delta }}_{(k,\sigma ),c}={\tilde{\rho }}_{(k,\sigma ),c}=\delta_{k,0}\rho_{c}\sqrt{2N+1}\sum_{b}\xi_{b,\sigma }\left(0\right)\;.$$

There are two different results corresponding to the two different eigenvalue labels σ=+ and σ=− in Equation (66). Summing over *b*, these are

(141) $$\begin{array}{ccc} {\tilde{\Delta }}_{(0,+),c} & = & {\tilde{\rho }}_{(0,+),c}=\rho_{c}\sqrt{N_{\mathrm{sites}}}\\ {\tilde{\Delta }}_{(0,-),c} & = & {\tilde{\rho }}_{(0,-),c}=0\;, \end{array}$$

where $N_{\mathrm{sites}}=2\left(2N+1\right)$. The interpretation of Equation (141) can be understood from Equation (77). The sum over *b* in Equation (140) is a sum over the element rows in Equation (77), which for σ=+ sums the mean charges per site on the two chains and for σ=− subtracts the charges per site on the two chains.

The fifth task of this section is to calculate the entropy for the spatially uniform saddle point, which is our mean-field value of the entropy. This requires the effective action at this saddle point, which becomes

(142) $$S_{\mathrm{eff},c}=-N_{\mathrm{sites}}\ln\left(\sum_{\gamma }a_{c}^{\left(\gamma \right)}\right)-\frac{\rho_{c}^{2}}{2}N_{\mathrm{sites}}\beta \;S_{\mathrm{lattice}}\;.$$

The grand canonical potential is then

(143) $$\Omega_{m=1}=\Omega_{c}=\frac{1}{\beta }S_{\mathrm{eff},c}\;.$$

We can now compute the entropy from the derivative of the grand thermodynamic potential with respect to the inverse temperature β, as in Section 2. This derivative becomes much more complicated with the introduction of $U_{\mathrm{int}}$ because the screened Coulomb potential (43) depends on β through the screening vector $q_{s}$ (44), where

(144) $$\frac{\partial q_{s}}{\partial \beta }=\frac{q_{s}}{2\beta }\;.$$

**Figure 17:**
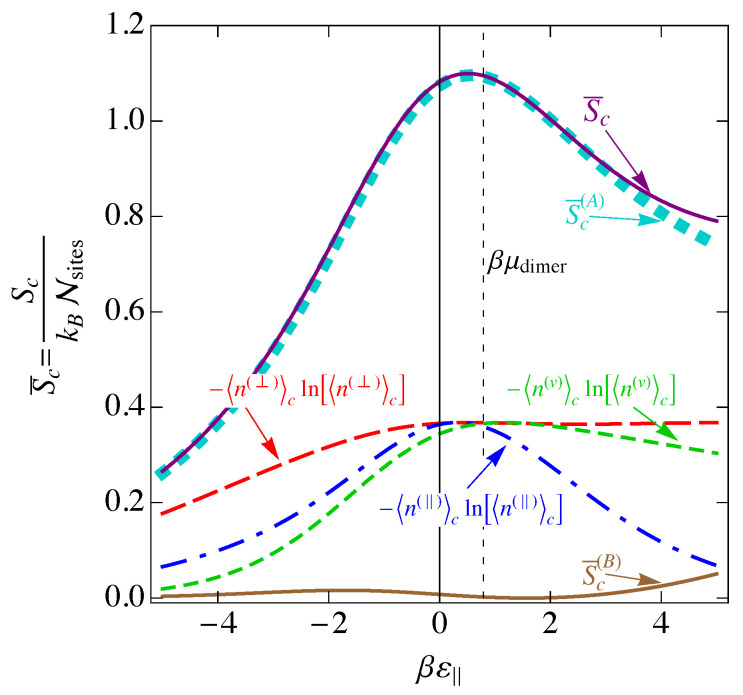
The functional form of mean-field entropy $\frac{S^{0}}{k_{B}N_{\mathrm{sites}}}$ for the interacting lattice gas model at the mean-field level. The constituent parts of the entropy are shown as well.

**Figure 18:**
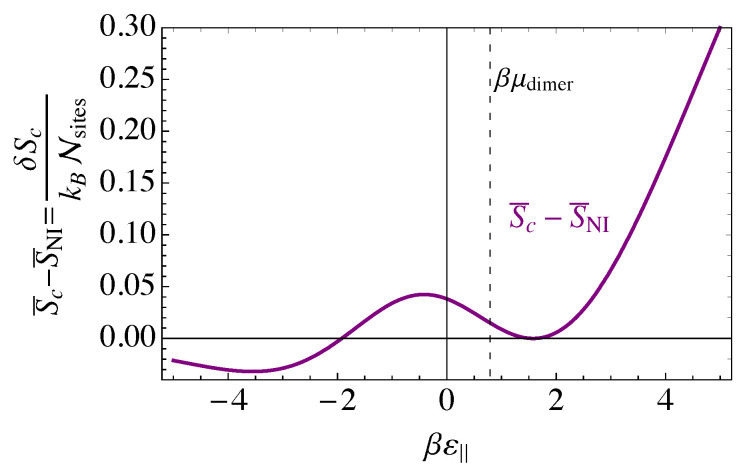
The difference between the mean-field entropy for the interacting lattice gas model and the entropy for the noninteracting model.

This introduces a β-dependence into both terms of Equation (142), so that the mean-field entropy is given by the more complex expression

(145) $${\bar{S}}_{c}=\frac{S_{c}}{k_{B}N_{\mathrm{sites}}}=\frac{S_{c}^{\left(A\right)}+S_{c}^{\left(B\right)}}{k_{B}N_{\mathrm{sites}}}\;,$$

where

(146) $${\bar{S}}_{c}^{\left(A\right)}=\frac{S_{c}^{\left(A\right)}}{k_{B}N_{\mathrm{sites}}}=-\sum_{\gamma }{\langle n^{\left(\gamma \right)}\rangle }_{c}\ln{\langle n^{\left(\gamma \right)}\rangle }_{c}$$

is the mixing entropy of the three species using the mean-field occupation numbers and

(147) $${\bar{S}}_{c}^{\left(B\right)}=\frac{S_{c}^{\left(B\right)}}{k_{B}N_{\mathrm{sites}}}=\frac{\rho_{c}^{2}}{2}\beta^{2}\;\frac{\partial S_{\mathrm{lattice}}}{\partial \beta }$$

arises directly from the self-energy $\Sigma \propto S_{\mathrm{lattice}}$. The occupation numbers ${\langle n^{\left(\gamma \right)}\rangle }_{c}$ used in this expression are those of the mean-field level, given by Equation (136) and shown in Figure 15. The mean-field entropy ${\bar{S}}^{\left(0\right)}$, given in Equation (145), is plotted in Figure 17, together with $-{\langle n^{\left(\gamma \right)}\rangle }_{c}\ln{\langle n^{\left(\gamma \right)}\rangle }_{c}$ for each species. Also shown are the two constituents ${\bar{S}}_{c}^{\left(A\right)}$ and ${\bar{S}}_{c}^{\left(B\right)}$ of the total entropy. This plot shows that, as with $S_{NI}$ in Figure 7, the mean-field entropy is greatest for $\varepsilon_{\|}$ near $\mu_{\mathrm{dimer}}$ and decreases outside this region. The difference $\delta {\bar{S}}_{c}=\frac{S_{c}-S_{\mathrm{NI}}}{k_{B}T}$ between this mean-field entropy for the interacting model and the entropy for the noninteracting model is plotted in Figure 18. Note that this curve has roughly the same shape as that of $\delta \sigma_{c}$ in Figure 16, with a large increase in disorder at large positive $\varepsilon_{\|}$ and small changes elsewhere.

By employing field-theoretic techniques borrowed from quantum mechanics, we have included the interaction energy $U_{\mathrm{int}}$ in the partition function and found a mean-field approximation to the exact partition function. However, these mean-field level calculations assume the same average charge $\rho_{c}$ for all DNA molecules in the same solution. Thus, mean-field calculations would predict a repulsive interaction between two DNA molecules, which would not lead to DNA condensation. Indeed, this repulsive interaction is all that this zeroth-order calculation can predict. It is the fluctuations in the charge from its mean-field value that enable the net attraction observed experimentally; to understand the attractive forces between DNA molecules in solution, it is necessary to extend this treatment by at least one additional order. The next order in the expansion (104) will yield Gaussian-type integrals, which are analytically solvable for the lowest-order fluctuations in the average charge and other parameters. Furthermore, once lowest-order fluctuations have been included, it will be possible to calculate thermodynamic properties of the system from the partition function to meaningful levels, such as the entropy changes due to small fluctuations in the local charge.

## 8. Inverse Hartree-Field-Fluctuation Propagator

In order to calculate the inverse Hartree-field-fluctuation propagator ${\tilde{D}}^{-1}$, we need to find the second derivatives of the effective action, evaluated at the saddle point. Since ${\tilde{D}}^{-1}$ is diagonal, from Equation (106), we can write

(148) $${\left({\tilde{D}}^{-1}\right)}_{\left(k_{1},\sigma_{1}\right),\left(k_{2},\sigma_{2}\right)}=\left(-\frac{1}{\beta \lambda_{k_{1},\sigma_{1}}}\right){\left.\frac{\partial^{2}S_{\mathrm{eff}}\left[\tilde{\Delta }\right]}{\partial {\tilde{\Delta }}_{k_{1},\sigma_{1}}\partial {\tilde{\Delta }}_{k_{2},\sigma_{2}}}\right|}_{{\tilde{\Delta }}_{c}}\;.$$

Since we already calculated the first derivative in Section 7, we need one more derivative, now with respect to ${\tilde{\Delta }}_{k_{2},\sigma_{2}}$. Substituting from Equation (121) yields

(149) $$\begin{array}{ccc} {\left({\tilde{D}}^{-1}\right)}_{\left(k_{1},\sigma_{1}\right),\left(k_{2},\sigma_{2}\right)} & = & \left(-\frac{1}{\beta \lambda_{k_{1},\sigma_{1}}}\right)\left.\frac{\partial }{\partial {\tilde{\Delta }}_{k_{2},\sigma_{2}}}\left\{-\beta \lambda_{k_{1},\sigma_{1}}{\tilde{\Delta }}_{k_{1},\sigma_{1}}+\right.\right.\\ & & {\left.\left.+\beta \lambda_{k_{1},\sigma_{1}}\sum_{\ell ,b}W_{\left(k_{1},\sigma_{1}\right),\left(\ell ,b\right)}^{T}\frac{\sum_{\gamma }q_{\gamma }{\tilde{a}}_{\ell ,b}^{\left(\gamma \right)}\left[\tilde{\Delta }\right]}{\sum_{\gamma^{\prime }}{\tilde{a}}_{\ell ,b}^{\left(\gamma^{\prime }\right)}\left[\tilde{\Delta }\right]}\right\}\right|}_{{\tilde{\Delta }}_{c}}\;. \end{array}$$

Performing the derivative, we have

(150) $$\begin{array}{ccc} {\left({\tilde{D}}^{-1}\right)}_{\left(k_{1},\sigma_{1}\right),\left(k_{2},\sigma_{2}\right)} & = & \left.\left\{1-\sum_{\ell ,b}W_{\left(k_{1},\sigma_{1}\right),\left(\ell ,b\right)}^{T}\frac{\sum_{\gamma }q_{\gamma }\frac{\partial {\tilde{a}}_{\ell ,b}^{\left(\gamma \right)}\left[\tilde{\Delta }\right]}{\partial {\tilde{\Delta }}_{k_{2},\sigma_{2}}}}{\sum_{\gamma^{\prime }}{\tilde{a}}_{\ell ,b}^{\left(\gamma^{\prime }\right)}\left[\tilde{\Delta }\right]}+\right.\right.\\ & & {\left.\left.+\sum_{\ell ,b}W_{\left(k_{1},\sigma_{1}\right),\left(\ell ,b\right)}^{T}\frac{\sum_{\gamma }q_{\gamma }{\tilde{a}}_{\ell ,b}^{\left(\gamma \right)}\left[\tilde{\Delta }\right]}{{\left(\sum_{\gamma^{\prime }}{\tilde{a}}_{\ell ,b}^{\left(\gamma^{\prime }\right)}\left[\tilde{\Delta }\right]\right)}^{2}}\sum_{\gamma^{\prime \prime }}\frac{\partial {\tilde{a}}_{\ell ,b}^{\left(\gamma^{\prime \prime }\right)}\left[\tilde{\Delta }\right]}{\partial {\tilde{\Delta }}_{k_{2},\sigma_{2}}}\right\}\right|}_{{\tilde{\Delta }}_{c}}\;. \end{array}$$

Substituting the derivative of the activity from Equations (119) and (120), we have

(151) $$\begin{array}{ccc} {\left({\tilde{D}}^{-1}\right)}_{\left(k_{1},\sigma_{1}\right),\left(k_{2},\sigma_{2}\right)} & = & \left.\left\{1+\beta \lambda_{k_{1},\sigma_{1}}\sum_{\ell ,b}W_{\left(k_{1},\sigma_{1}\right),\left(\ell ,b\right)}^{T}W_{\left(k_{2},\sigma_{2}\right),\left(\ell ,b\right)}^{T}\times \right.\right.\\ & & {\left.\left.\times \left[\frac{\sum_{\gamma }q_{\gamma }^{2}{\tilde{a}}_{\ell ,b}^{\left(\gamma \right)}}{\sum_{\gamma^{\prime }}{\tilde{a}}_{\ell ,b}^{\left(\gamma^{\prime }\right)}\left[\tilde{\Delta }\right]}-{\left(\frac{\sum_{\gamma }q_{\gamma }{\tilde{a}}_{\ell ,b}^{\left(\gamma \right)}\left[\tilde{\Delta }\right]}{\sum_{\gamma^{\prime }}{\tilde{a}}_{\ell ,b}^{\left(\gamma^{\prime }\right)}\left[\tilde{\Delta }\right]}\right)}^{2}\right]\right\}\right|}_{{\tilde{\Delta }}_{c}}\;. \end{array}$$

The quantity in square brackets is just the constant $\sigma_{c}^{2}$, the variance in the charge per site, given in Equation (137). Since W from Equations (74)–(76), is an orthogonal matrix,

(152) $$\sum_{\ell ,b}W_{\left(k_{1},\sigma_{1}\right),\left(\ell ,b\right)}^{T}W_{\left(k_{2},\sigma_{2}\right),\left(\ell ,b\right)}^{T}=\delta_{k_{1}k_{2}}\delta_{\sigma_{1}\sigma_{2}}\;,$$

and this means that the matrix is diagonal. With these substitutions, the inverse Hartree-field-fluctuation propagator becomes

(153) $${\left({\tilde{D}}^{-1}\right)}_{\left(k_{1},\sigma_{1}\right),\left(k_{2},\sigma_{2}\right)}=\delta_{k_{1}k_{2}}\delta_{\sigma_{1}\sigma_{2}}\left(1+\sigma_{c}^{2}\beta \lambda_{k_{1},\sigma_{1}}\right)\;.$$

This is the inverse propagator for the fluctuations of the auxiliary Hartree field that we introduced to decouple the interparticle interactions, expressed in the diagonal basis.

**Figure 19:**
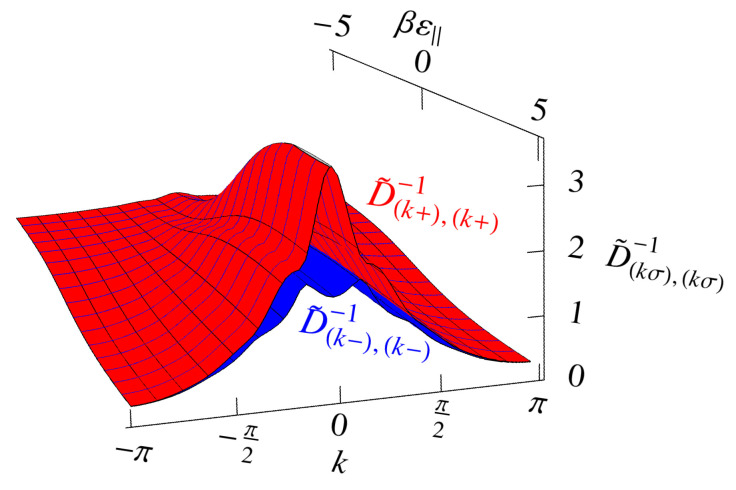
The inverse propagator for the fluctuations of the auxiliary Hartree field ${\left({\tilde{D}}^{-1}\right)}_{\left(k_{1},\sigma_{1}\right),\left(k_{2},\sigma_{2}\right)}$.

**Figure 20:**
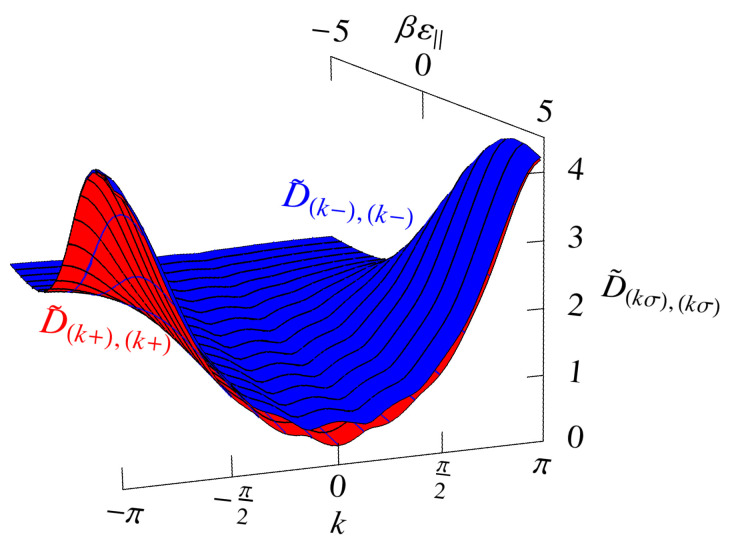
The propagator for the fluctuations of the auxiliary Hartree field ${\tilde{D}}_{\left(k_{1},\sigma_{1}\right),\left(k_{2},\sigma_{2}\right)}$.

The inverse propagator ${\left({\tilde{D}}^{-1}\right)}_{\left(k_{1},\sigma_{1}\right),\left(k_{2},\sigma_{2}\right)}$ in the diagonal basis is plotted in Figure 19 for the two eigenvalues $\lambda_{k\pm }$, shown in Figure 14. Notice that the shapes are those of the eigenvalues, but shifted upward. They both have minima at k=π, with the one corresponding to $\lambda_{k+}$ having its maximum at k=0 and the one corresponding to $\lambda_{k-}$ having maxima near k=0. The corresponding plots of the propagator ${\tilde{D}}_{\left(k_{1},\sigma_{1}\right),\left(k_{2},\sigma_{2}\right)}$ are shown in Figure 20, also in the diagonal basis. The propagator increases with $\beta \varepsilon_{\|}$ near k=±π, and this can be attributed, at least in part, to the increase in $\sigma_{c}$, shown in Figure 16, because of the dependence shown in Equation (151). Although the auxiliary field propagator normally describes the behavior of fluctuations of a physical field like the charge, its definition given by Equation (110) shows that the field here has been rescaled by the square root of the eigenvalue. This was discussed earlier in the context of entropy in the paragraph after Equation (92), and so this form is convenient for the entropy calculation, our focus here, but its interpretation is not quite so direct.

## 9. Inclusion of Lowest-Order Fluctuations

Going beyond the mean field to one-loop order requires use of the full partition function given by Equations (111) and (113). This has consequences for the site occupancies, the average charge per site, the charge variance, and the entropy. The expression for site occupancy has the same form in terms of the partition function as for the noninteracting and mean-field cases in Equations (9) and (86), and can be written explicitly for the one-loop order of approximation as

(154) $${\langle n^{\left(\gamma \right)}\rangle }_{\mathrm{tot}}=\frac{1}{N_{\mathrm{sites}}\beta Z_{G}}\frac{\partial }{\partial \mu_{\gamma }}\left[\frac{e^{-S_{\mathrm{eff}}\left[{\tilde{\Delta }}_{c}\right]}}{\sqrt{\det({\tilde{D}}^{-1})}}\right]\;,$$

which is obtained from Equation (116) with *m* set equal to one. In that expression, *m* was the parameter that was introduced in Section 6 as an aid to identifying the various orders in the expansion. This can be written as the sum of two terms,

(155) $${\langle n^{\left(\gamma \right)}\rangle }_{\mathrm{tot}}={\langle n^{\left(\gamma \right)}\rangle }_{c}+\delta \langle n^{\left(\gamma \right)}\rangle \;,$$

where ${\langle n^{\left(\gamma \right)}\rangle }_{c}$ is the occupancy of species γ at the mean-field level, given by Equation (136),

(156) $${\langle n^{\left(\gamma \right)}\rangle }_{c}=-\frac{1}{N_{\mathrm{sites}}\beta }\frac{\partial }{\partial \mu_{\gamma }}S_{\mathrm{eff}}\left[{\tilde{\Delta }}_{c}\right]\;,$$

and $\delta \langle n^{\left(\gamma \right)}\rangle$ is the correction to that mean site occupancy due to fluctuations, which is given by

(157) $$\delta \langle n^{\left(\gamma \right)}\rangle =\frac{1}{N_{\mathrm{sites}}\beta }\frac{\partial }{\partial \mu_{\gamma }}\ln\left[\det\left({\tilde{D}}^{-1}\right)\right]\;,$$

with the inverse propagator ${\tilde{D}}_{\left(k_{1},\sigma_{1}\right),\left(k_{2},\sigma_{2}\right)}^{-1}$ from Equation (153) written in matrix form as

(158) $${\tilde{D}}^{-1}=I+\sigma_{c}^{2}\beta \Lambda \;,$$

where *I* is the identity matrix and Λ is the (diagonal) matrix of eigenvalues of the potential. Since ${\tilde{D}}^{-1}$ is diagonal, its determinant is given by the product of its diagonal elements as

(159) $$\det{\tilde{D}}^{-1}=\prod_{k,\sigma }\left(1+\beta \lambda_{k,\sigma }\sigma_{c}^{2}\right)\;.$$

The logarithm of this determinant then gives the sum over logarithms of diagonal elements as

(160) $$\ln\left(\det{\tilde{D}}^{-1}\right)=\sum_{k,\sigma }\ln\left(1+\beta \lambda_{k,\sigma }\sigma_{c}^{2}\right)\;.$$

The only $\mu_{\gamma }$ dependence is contained in $\sigma_{c}^{2}$, and its derivative is given by

(161) $$\frac{\partial \sigma_{c}^{2}}{\partial \mu_{\gamma }}=\beta {\langle n^{\left(\gamma \right)}\rangle }_{c}\left[q_{\gamma }^{2}-{\langle \rho^{2}\rangle }_{c}-2\rho_{c}\left(q_{\gamma }-\rho_{c}\right)\right]\;,$$

so that the derivative of $\ln(\det D^{-1})$ is given by the coefficient

(162) $$C=\frac{1}{2N_{\mathrm{sites}}}\sum_{k,\sigma }\frac{\beta \lambda_{k\sigma }}{\left[1+\beta \lambda_{k,\sigma }\sigma_{c}^{2}\right]}\;.$$

Then, the mean fluctuation in occupancy $\delta \langle n^{\left(\gamma \right)}\rangle$ becomes

(163) $$\delta \langle n^{\left(\gamma \right)}\rangle =C{\langle n^{\left(\gamma \right)}\rangle }_{c}\left[q_{\gamma }^{2}-{\langle \rho^{2}\rangle }_{c}-2\rho_{c}\left(q_{\gamma }-\rho_{c}\right)\right]\;.$$

**Figure 21:**
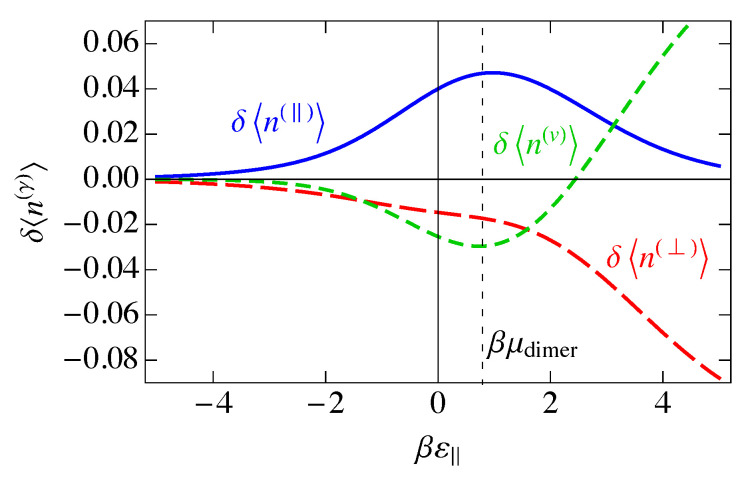
The changes in the occupancies $\delta \langle n^{\left(\gamma \right)}\rangle$ due to fluctuations. These should be compared with the changes between occupancies of the mean-field and noninteracting systems shown in Figure 15.

These changes in the mean-field occupancies at this one-loop order are plotted in Figure 21. These changes are not large, and the fluctuation corrections amount to no more than about 10% of the total. The most dramatic effects are seen in the region of large positive $\varepsilon_{\|}$, with the corrections to the vacancies and perpendicular dimers going in the opposite direction to the corrections due to the mean field in Figure 15. This can be interpreted as a relaxation of the rigid penalties imposed by the electrostatic self-energies at the mean-field level. An enhancement in parallel dimers is also seen across the whole range of $\varepsilon_{\|}$ that peaks around $\varepsilon_{\|}=\mu_{\mathrm{dimer}}$.

The mean charge per site at this one-loop-correction level of approximation is given by the same form as Equations (22) and (130) for the noninteracting and mean-field approximations and is given by

(164) $$\rho_{\mathrm{tot}}={\langle \rho \rangle }_{\mathrm{tot}}=\sum_{\gamma }q_{\gamma }{\langle n^{\left(\gamma \right)}\rangle }_{\mathrm{tot}}\;,$$

where now ${\langle n^{\left(\gamma \right)}\rangle }_{\mathrm{tot}}$ is taken from Equations (155) and (163). and the charge per site becomes

(165) $$\rho_{\mathrm{tot}}=\rho_{c}+\delta \rho_{\mathrm{tot}}\;,$$

where

(166) $$\delta \rho_{\mathrm{tot}}=\rho_{c}C\left(1-3{\langle \rho^{2}\rangle }_{c}+2\rho_{c}^{2}\right)\;.$$

**Figure 22:**
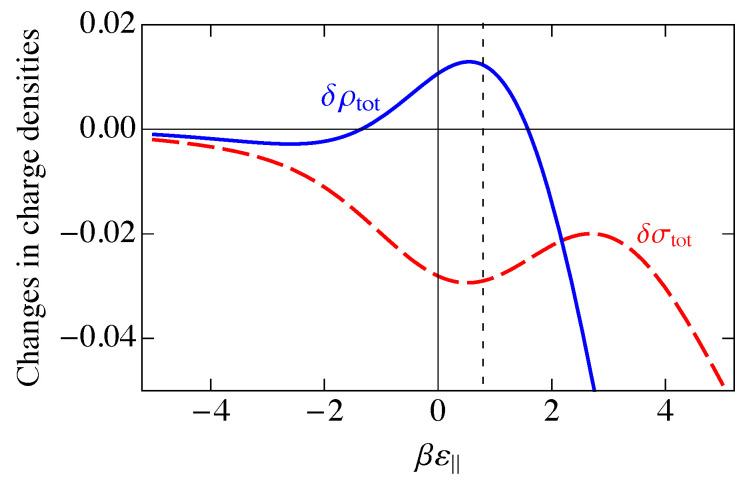
The changes in the charge per site $\delta \rho_{\mathrm{tot}}$ and in the standard deviation $\delta \sigma_{\mathrm{tot}}$ of the charge per site due to fluctuations. These are further corrections to the mean-field values of Figure 16.

The change in the charge per site $\delta \rho_{\mathrm{tot}}$ is shown as the solid blue curve in Figure 22. The most notable features are a negative contribution to the total charge above $\beta \varepsilon_{\|}\approx 2$, reflecting the addition of more negatively charged vacancies due to the fluctuations, and a positive contribution to the total charge around $\varepsilon_{\|}\approx \mu_{\mathrm{dimer}}$. Interestingly, this positive contribution is not associated with the increase in parallel dimer occupancy, since parallel dimers are electrically neutral. Rather, it is due to the fact that, although both the negatively charged vacancies and positively charged perpendicular dimers are both reduced around $\varepsilon_{\|}\approx \mu_{\mathrm{dimer}}$, the vacancies are decreased more.

The variance in the charge is given by

(167) $$\sigma_{\mathrm{tot}}^{2}={\langle \rho^{2}\rangle }_{\mathrm{tot}}-\rho_{\mathrm{tot}}^{2}\;.$$

The average of the square of the charge per site is given by

(168) $${\langle \rho^{2}\rangle }_{\mathrm{tot}}=\sum_{\gamma }q_{\gamma }^{2}{\langle n^{\left(\gamma \right)}\rangle }_{\mathrm{tot}}$$

and is written explicitly as

(169) $${\langle \rho^{2}\rangle }_{\mathrm{tot}}={\langle \rho^{2}\rangle }_{c}+\delta {\langle \rho^{2}\rangle }_{\mathrm{tot}}\;,$$

where ${\langle \rho^{2}\rangle }_{c}$ is given by Equation (138), and $\delta {\langle \rho^{2}\rangle }_{\mathrm{tot}}$ works out to be

(170) $$\delta {\langle \rho^{2}\rangle }_{\mathrm{tot}}=C\left[{\langle \rho^{2}\rangle }_{c}\left(1-{\langle \rho^{2}\rangle }_{c}-2\rho_{c}^{2}\right)-2\rho_{c}^{2}\right]\;.$$

The charge variance can then be written as

(171) $$\sigma_{\mathrm{tot}}^{2}=\sigma_{c}^{2}+\sigma_{f}^{2}\;,$$

where the fluctuation correction is

(172) $$\begin{array}{ccc} \sigma_{f}^{2} & = & -{\langle \rho^{2}\rangle }_{c}^{2}C\left(1+9\rho_{c}^{2}C\right)+{\langle \rho^{2}\rangle }_{c}C\left[\left(1+8\rho_{c}^{2}\right)+6\rho_{c}^{2}C\left(1+2\rho_{c}^{2}\right)\right]\\ & - & \rho_{c}^{2}C\left[4\left(1+\rho_{c}^{2}\right)-C\left(1+4\rho_{c}^{2}+4\rho_{c}^{4}\right)\right]\;. \end{array}$$

The standard deviation can then be written as

(173) $$\sigma_{\mathrm{tot}}=\sigma_{c}+\delta \sigma_{\mathrm{tot}}\;,$$

The change $\delta \sigma_{\mathrm{tot}}$ in the standard deviation of the charges per site due to fluctuations is given in Figure 22, where the fluctuations account for changes typically of the order of 10% of the mean-field value.

Having obtained results for the effects of fluctuations on the mean occupancies and charges per site, we now consider the corresponding corrections to the entropy. For that, we need the grand thermodynamic potential, which was expanded in Section 6 as Equations (115)–(117), with the mean-field and lowest-order fluctuation terms given by

(174) $$\Omega_{G}≃\Omega_{c}+\frac{1}{2\beta }\ln\det D^{-1}\;.$$

The corresponding expression for the interacting entropy is

(175) $$S_{\mathrm{tot}}=S_{c}+\delta S_{\mathrm{tot}}\;,$$

where the mean-field entropy $S_{c}$ was derived and the results presented in Section 7 in Equations (145)–(147). The fluctuation contribution to the entropy $\delta S_{\mathrm{tot}}$ is given by

(176) $$\delta S_{\mathrm{tot}}=k_{B}\beta^{2}\frac{\partial }{\partial \beta }\left\{\frac{1}{2\beta }\ln\left[\det\left(D^{-1}\right)\right]\right\},$$

which requires performing another derivative.

To evaluate this contribution to the entropy $\delta S_{\mathrm{tot}}$ in Equation (176), we return to Equation (160) and substitute in the fluctuation contribution to the entropy from Equation (176), which after performing derivatives gives

(177) $$\delta S_{\mathrm{tot}}=S_{f}^{\left(A\right)}+S_{f}^{\left(B\right)}\;,$$

where

(178) $$S_{f}^{\left(A\right)}=-\frac{k_{B}}{2}\sum_{k,\sigma }\ln\left(1+\beta \lambda_{k,\sigma }\sigma_{c}^{2}\right)$$

and

(179) $$S_{f}^{\left(B\right)}=\frac{k_{B}}{2}\sum_{k,\sigma }\frac{1}{\left(1+\beta \lambda_{k,\sigma }\sigma_{c}^{2}\right)}\left(\beta \lambda_{k,\sigma }\sigma_{c}^{2}+\sigma_{c}^{2}\beta^{2}\frac{\partial \lambda_{k,\sigma }}{\partial \beta }+\beta \lambda_{k,\sigma }\beta \frac{\partial \sigma_{c}^{2}}{\partial \beta }\right)\;.$$

The derivative of the eigenvalue $\lambda_{k,\sigma }$ of the potential is given by

(180) $$\frac{\partial \lambda_{k,\sigma }}{\partial \beta }=\frac{\partial }{\partial \beta }{\tilde{V}}_{↑↑}\left(k\right)+\sigma \frac{\partial }{\partial \beta }\left|{\tilde{V}}_{↑↓}\left(k\right)\right|\;,$$

where ${\tilde{V}}_{↑↑}\left(k\right)$ is the Fourier transform of the interaction potential of a chain with itself and ${\tilde{V}}_{↑↓}\left(k\right)$ is the Fourier transform of the interaction potential between the two chains. Both of these potentials depend on the screening vector $q_{s}$, and the only β dependence of the potential is contained in $q_{s}$, which is proportional to $\sqrt{\beta }$. The derivative we need is then given by Equation (144). As a consequence, the derivative of the Fourier transform of the potential is

(181) $$\frac{\partial {\tilde{V}}_{b_{1},b_{2}}\left(k\right)}{\partial \beta }=\frac{1}{2}\sum_{\ell =-N}^{N}\left[e^{-ik\ell }\frac{\partial V_{b_{1},b_{2}}\left(\ell \right)}{\partial \beta }+e^{ik\ell }\frac{\partial V_{b_{2},b_{1}}\left(\ell \right)}{\partial \beta }\right]\;,$$

where the derivative of the potential in the position basis is given by

(182) $$\frac{\partial V_{b_{1},b_{2}}\left(\ell \right)}{\partial \beta }=V_{b_{1},b_{2}}\left(\ell \right)\left(-\frac{q_{s}}{2\beta }d_{b_{1},b_{2}}\left(\ell \right)\right)\;.$$

Substituting and using the symmetry relations in Equations (47) and (50), the derivative of the Fourier transform of the potential is

(183) $$\frac{\partial {\tilde{V}}_{b_{1},b_{2}}\left(k\right)}{\partial \beta }=-\frac{q_{s}}{2\beta }\sum_{\ell =-N}^{N}e^{-ik\ell }V_{b_{1},b_{2}}\left(\ell \right)d_{b_{1},b_{2}}\left(\ell \right)\;.$$

Using these relations, the derivative of the eigenvalues becomes

(184) β2∂λk,σ∂β=−qs2∑ℓ=−NNβV↑↑(ℓ)d↑↑(ℓ)cos(kℓ)+σV↑↓(ℓ)d↑↓(ℓ)Re[V˜↑↓(k)]|V˜↑↓(k)|cos(kℓ)−Im[V˜↑↓(k)]|V˜↑↓(k)|sin(kℓ),

where Re[V˜↑↓(k)] and Im[V˜↑↓(k)] are the real and imaginary parts of ${\tilde{V}}_{↑↓}\left(k\right)$.

Equation (177) also requires the derivative of the variance $\sigma_{c}^{2}$, where $\sigma_{c}^{2}$ is given by Equation (137). This contains the average charge per site from Equation (130) and the average of the square of the charge per site from Equation (138). This derivative is

(185) $$\begin{array}{ccc} \beta \frac{\partial \sigma_{c}^{2}}{\partial \beta } & = & \frac{\rho_{c}}{1+\sigma_{c}^{2}\beta S_{\mathrm{lattice}}}\left\{\left({\left[\rho E\right]}_{\mathrm{av}}-\rho_{c}E_{c}\right)\left[2+\left(1-{\langle \rho^{2}\rangle }_{c}\right)\beta S_{\mathrm{lattice}}\right]+\right.\\ & & +\left.\rho_{c}\left(3\sigma_{c}^{2}+\rho_{c}^{2}-1\right)\beta^{2}\frac{\partial S_{\mathrm{lattice}}}{\partial \beta }\right\}-\left({\left[\rho^{2}E\right]}_{\mathrm{av}}-{\langle \rho^{2}\rangle }_{c}E_{c}\right)\;, \end{array}$$

where

(186) $$E\equiv \beta (\varepsilon_{\gamma }-\mu_{\gamma }+\rho_{c}q_{\gamma }S_{\mathrm{lattice}})\;,$$

(187) $$E_{c}=\sum_{\gamma }E{\langle n^{\left(\gamma \right)}\rangle }_{c}\;,$$

(188) $${\left[\rho E\right]}_{\mathrm{av}}=\sum_{\gamma }q_{\gamma }E{\langle n^{\left(\gamma \right)}\rangle }_{c}\;,$$

and

(189) $${\left[\rho^{2}E\right]}_{\mathrm{av}}=\sum_{\gamma }q_{\gamma }^{2}E{\langle n^{\left(\gamma \right)}\rangle }_{c}\;.$$

Here, ${\left[\cdots \right]}_{\mathrm{av}}$ is the weighted average of the specified quantity over the distribution given by the mean-field occupancies ${\langle n^{\left(\gamma \right)}\rangle }_{c}$ of the three species.

It is interesting to note that the combinations ${\left[\rho^{2}E\right]}_{\mathrm{av}}-{\langle \rho^{2}\rangle }_{c}E_{c}$ and ${\left[\rho E\right]}_{\mathrm{av}}-\rho_{c}E_{c}$ can be written in forms similar to that of the zeroth-order entropy. These forms are

(190) $${\left[\rho^{2}E\right]}_{\mathrm{av}}-{\langle \rho^{2}\rangle }_{c}E_{c}=-\sum_{\gamma }\left[{\langle n^{\left(\gamma \right)}\rangle }_{c}\ln{\langle n^{\left(\gamma \right)}\rangle }_{c}\left(1-\sum_{\gamma^{\prime }}{\langle n^{\left(\gamma^{\prime }\right)}\rangle }_{c}\ln{\langle n^{\left(\gamma^{\prime }\right)}\rangle }_{c}\right)\right]$$

and

(191) $${\left[\rho E\right]}_{\mathrm{av}}-\rho_{c}E_{c}=-\sum_{\gamma }q_{\gamma }{\langle n^{\left(\gamma \right)}\rangle }_{c}\ln{\langle n^{\left(\gamma \right)}\rangle }_{c}+\rho_{c}\sum_{\gamma }{\langle n^{\left(\gamma \right)}\rangle }_{c}\ln{\langle n^{\left(\gamma \right)}\rangle }_{c}\;.$$

**Figure 23:**
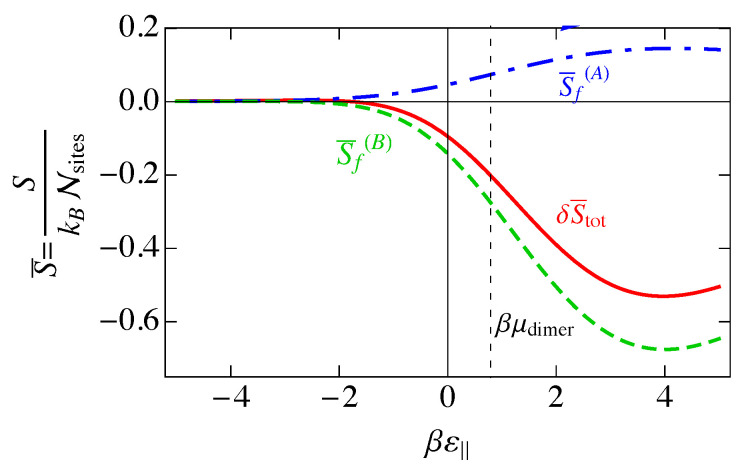
The fluctuation contribution to the entropy ${\bar{S}}_{f}=\frac{S_{f}}{k_{B}N_{\mathrm{sites}}}$ per site. The two contributions, ${\bar{S}}_{f}^{\left(A\right)}$ and ${\bar{S}}_{f}^{\left(B\right)}$, to the total entropy are also shown, and they are defined similarly.

The fluctuation entropy contribution $S_{f}^{\left(A\right)}$ is easy to calculate, given the eigenvalues $\lambda_{k,\sigma }$. Equations (180)–(191) simply collect the results that allow us to calculate $S_{f}^{\left(B\right)}$. The results for the total fluctuation entropy $\delta S_{\mathrm{tot}}$ and the two contributions to it are shown in Figure 23 for $\varepsilon_{⊥}=\frac{1}{2}\varepsilon_{\|}$. Like the mean-field entropy, the fluctuation entropy becomes larger in magnitude as the binding becomes weak. However, the fluctuation contribution is actually negative in this region, suggesting that the fluctuations are leading to increasing order. These corrections to the entropy are typically an order of magnitude larger than the difference between the mean field and noninteracting values of the entropy.

## 10. Discussion

Entropy forms an important contribution to the free energies of many biological systems. Here, we summarize the results for the entropies and particle numbers per site for a lattice gas model representing the adsorption of molecules on double-stranded DNA. The entropies and particles per site are presented at three successive levels of approximation, first, for the noninteracting system, then at the mean-field level, and finally with the inclusion of fluctuations to one-loop order. Those fluctuation corrections result from correlations induced by the electrostatic forces among the dimer molecules themselves and with the electrically charged DNA substrate.

**Figure 24:**
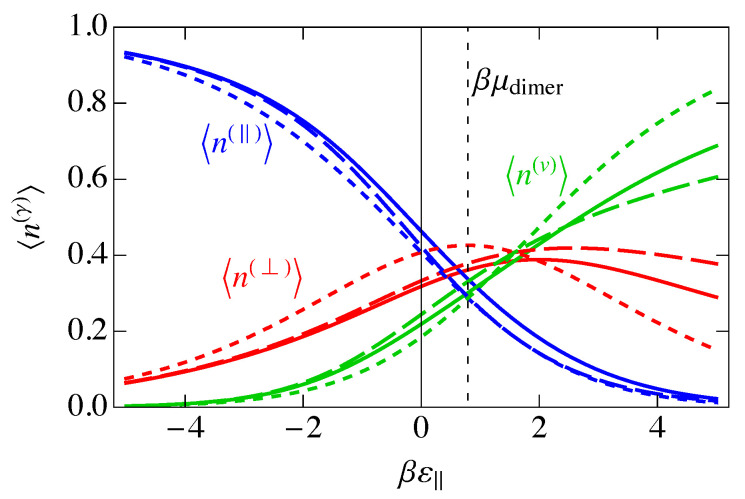
The average occupation numbers in the noninteracting approximation (${\langle n^{\left(\gamma \right)}\rangle }_{NI}$), shown as dotted curves, the mean-field approximation (${\langle n^{\left(\gamma \right)}\rangle }_{c}$), shown as dashed curves, and the model including the lowest-order corrections to the mean field, shown as solid curves, plotted for γ=‖ (blue), ⊥ (red), and *v* (green). Parameters used in the plot are $\beta \mu_{\mathrm{dimer}}=0.79$ and $\varepsilon_{\|}=2\varepsilon_{⊥}$.

**Figure 25:**
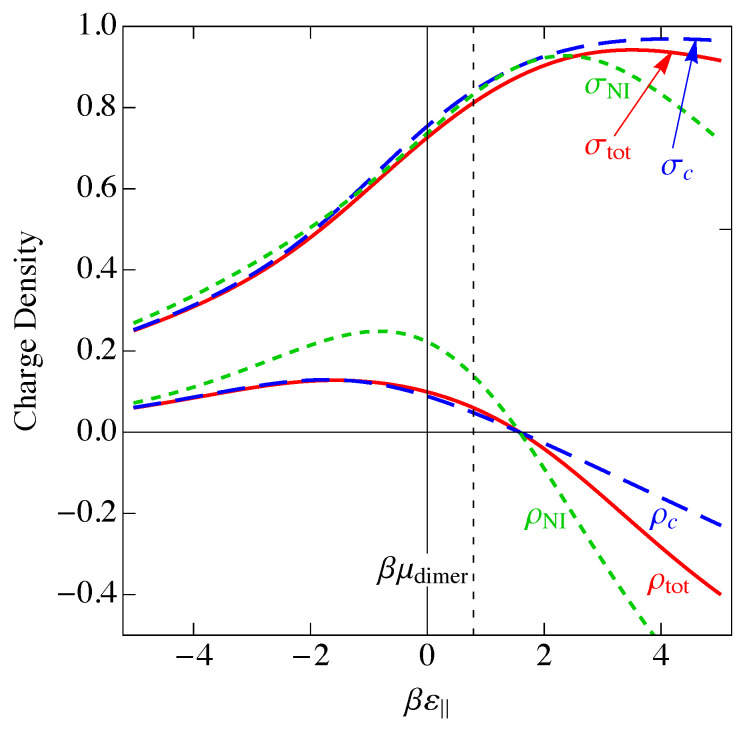
The average charge per site in the noninteracting ${\langle \rho \rangle }_{NI}$ (green, short dashes) and mean-field (blue, long dashes) systems, and with inclusion of fluctuations (red, solid). This plot assumes $\beta \mu_{\mathrm{dimer}}=0.79$ and $\varepsilon^{\left(\|\right)}=2\varepsilon^{\left(⊥\right)}$. Note that as $\beta \varepsilon_{\|}\to \infty$, all the dimers will detach from the DNA, and the charges per site $\rho_{NI}$, $\rho_{c}$, and $\rho_{tot}$ will all approach −1.

Perhaps the most transparent descriptors of the DNA system’s behavior are the average occupation numbers $\langle n^{\left(\|\right)}\rangle$, $\langle n^{\left(⊥\right)}\rangle$, and $\langle n^{\left(v\right)}\rangle$ representing the thermal average of the numbers of parallel-adsorbed dimers, perpendicular-adsorbed dimers, and vacancies, respectively, on each site. These particles per site are proportional to the probability that a given site would be found to be occupied by a particular species. These site-occupation numbers are shown in Figure 24 for the three levels of approximation. The short-dashed curves are for the noninteracting level of approximation ${\langle n^{\left(\gamma \right)}\rangle }_{\mathrm{NI}}$, the long-dashed curves for the mean-field level ${\langle n^{\left(\gamma \right)}\rangle }_{c}$, and the solid curves for the inclusion of fluctuations ${\langle n^{\left(\gamma \right)}\rangle }_{\mathrm{tot}}$. The corresponding charges per site and standard deviations of the charge density are shown in Figure 25.

The site occupancies exhibit similar qualitative behavior in all three levels of approximation. Due to the energetics of binding $\varepsilon_{\|}=2\varepsilon_{⊥}$, parallel dimers occupy the greatest fraction of sites for all negative binding energies $\left(\varepsilon_{\|},\varepsilon_{⊥}<0\right)$, followed by the perpendicular dimers, and then the vacancies, at least for the parameters used in these calculations. At weak binding ($\varepsilon_{\|},\varepsilon_{⊥}\to 0$) in the noninteracting system, the difference between these energies becomes negligible, and both $n^{\left(\|\right)}$ and $n^{\left(⊥\right)}$ approach the same value of 40%. When the binding energy $\varepsilon_{\|}$ or $\varepsilon_{⊥}$ increases and reaches the chemical potential $\mu_{\mathrm{dimer}}$ of the dimers in solution, they are repelled from the surface of the DNA and go into solution, leaving a “naked” lattice of negatively charged vacancies, with the parallel dimers being more repelled than perpendicular ones.

In contrast to the case of noninteracting sites, the inclusion of interactions between sites gives a pronounced gap of about 25% by which parallel adsorption exceeds perpendicular adsorption in the weak-binding limit. This enhancement in parallel adsorption in the mean-field approximation results from the inclusion of the charge on the perpendicular dimers in an average or mean-field sense, which penalizes the addition of positively charged perpendicular dimers when the total charge on the DNA lattice is positive. Fluctuations reduce this effect slightly. Recall that the perpendicular dimers are positively charged because one end is not attached to a negative binding site, and this means that it is energetically unfavorable to have a second one nearby. Parallel binding, on the other hand, neutralizes the negative charge on the sites, so that a DNA double strand completely covered in parallel-adsorbed dimers would be electrically neutral. The decrease in perpendicular-dimer occupancy caused by this electrostatic repulsion of like charges leads to an increase in sites occupied by vacancies. Vacancies are negatively charged and are energetically favored when the perpendicular dimers provide a positively charged region. There may, in fact, be correlations resulting from the lower electrostatic energy of a configuration in which sites alternate between perpendicular dimers and vacancies, i.e., between positively and negatively charged sites, in a Wigner-lattice-like state similar to those described in the literature [8,19,26].

The average charge $\rho_{c}$ per site depends only on the perpendicular dimers and vacancies since the parallel dimers neutralize sites, so that the charge per site is written simply as

(192) $$\rho =\sum_{\gamma }q_{\gamma }\langle n^{\left(\gamma \right)}\rangle =\langle n^{\left(⊥\right)}\rangle -\langle n^{\left(v\right)}\rangle \;,$$

and this connection can be seen in the plots of charge per site ρ in Figure 25, when compared with Figure 24. For all approximations, the average charge is positive at negative and slightly positive binding energies, indicating charge inversion. This is a result of the occupation numbers shown in Figure 24, because parallel binding, which neutralizes the charge, increasingly dominates in the strong-binding limit ($\varepsilon_{\|}=2\varepsilon_{⊥}\ll 0$). Similarly, the decrease in the magnitude of the charge inversion from the noninteracting to the mean-field approximations can be explained by the enhancement in parallel adsorption and reduction of perpendicular adsorption due to like-charge repulsion. The fluctuation corrections to the site occupancies are not large when compared with the mean-field values. They only seem significant at positive binding energies where the site occupancies are shifted in the direction of the noninteracting system. Of course, the site occupancies are single-particle properties and do not measure spatial correlations.

**Figure 26:**
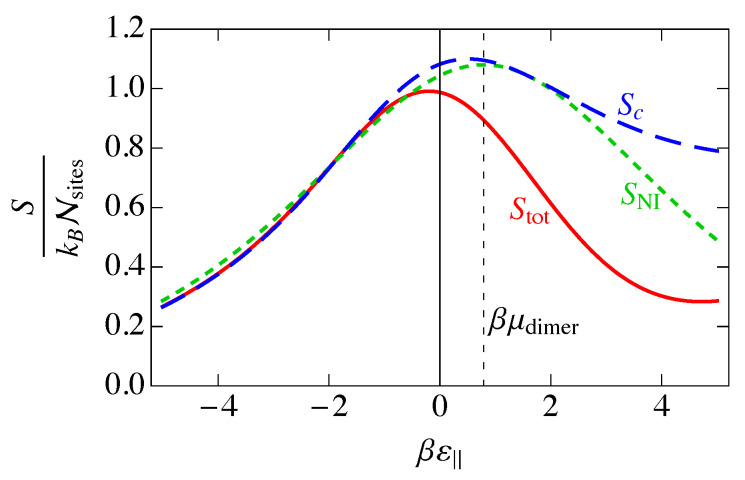
The entropy *S* per site, in units of the Boltzmann constant $k_{B}$, in the noninteracting (green, short dashes), mean-field (blue, long dashes), and inclusion of fluctuations (red, solid) approximations. These curves assume $\beta \mu_{\mathrm{dimer}}=0.79$ and $\varepsilon_{\|}=2\varepsilon_{⊥}$.

The behavior of the entropy is shown in Figure 26, in which the entropy per site is plotted versus $\beta \varepsilon_{\|}$ for all three levels of approximation. The three curves are practically indistinguishable for strong binding (negative values of $\beta \varepsilon_{\|}$) until the binding energy becomes quite weak (approaching zero), indicating little effect of correlations induced by electrostatic repulsion on the entropy in that region. This is because the dimers are closely packed, leaving little freedom for disorder. The pronounced differences are seen in the region of $\varepsilon_{\|}\approx \mu_{\mathrm{dimer}}$ and more positive binding energies ($\varepsilon_{\|}=2\varepsilon_{⊥}\gg 0$), because the system becomes more disordered. There, the dimers tend to leave the surface and enter the solution, and the fluctuation corrections to the entropy are greater in that region and act to reduce the entropy. This shows that the electrostatic correlations increase the order and reduce the entropy. Surprisingly, this decrease in entropy at large positive $\varepsilon_{\|}$ due to fluctuations is so significant that it *overcompensates* for the entropy generated at the mean-field level, leading to a total entropy $S_{\mathrm{tot}}$ that is even lower than in the noninteracting case $S_{\mathrm{NI}}$.

The form of the noninteracting entropy comes from the lattice gas model, in which parallel adsorption is treated as “local”, only occupying one site. When the geometrical blocking effect is included, in which parallel-adsorbed dimers occupy two sites, we would expect the entropy to differ significantly from the nlnn result even at the noninteracting level. It is unclear how large a contribution to the full entropy the blocking effect provides, but it is certainly lower for dimers than for longer polymers, for which the nonlocality is greater. Because of this, it will be important in future work to develop a way of incorporating the blocking effect into these types of field-theoretic models.

Dimers are certainly the simplest example of polymers, although biological polymers are typically considerably longer. There has been extensive work on the dimer model, beginning with the work of Fisher [52,53]. Some of this work has been motivated by the analogy between dimer models and quantum spin systems [36]. In this context, Fisher has derived expressions for the contributions of hard-core crowding effects to the partition function, including the geometrical blocking effect. Fisher’s results include an expression of the noninteracting partition function for dimers on a 1D linear lattice. In a future publication, we plan to adapt Fisher’s approach to study the statistical consequences of these blocking effects [54].

In addition, it has been suggested that methods can be adapted from analogous problems in particle physics [37] and condensed matter physics [55]. This involves using numerical codes like those of Adams and Chandrasekharan [37] that are used for simulations in lattice quantum chromodynamics.

Modeling the behavior of DNA and other biologically active polymeric molecules under physiological conditions is an area in which quantitative calculation is particularly difficult. This is because it is necessary to accommodate simultaneously the influences of geometry, electrostatic forces, and charge correlations. All of these play roles of varying significance in determining the properties of these molecules. Despite these challenges, considerable progress has been made in extending our physical understanding of these systems, and some surprising new physics, such as charge inversion and condensation, have been discovered in the process.

The work presented here focuses on electrostatic effects and especially on the role they play in the entropy, with the overall goal of determining the entropy as a contribution to the free energy and as a driving force in biochemical reactions. For the DNA-based model that we consider, we find that, particularly in the low-coverage regime, a uniform mean-field theory gives changes ∼20% from the usual noninteracting entropy term. We also find, though, that the fluctuation contributions are both considerably larger and in the opposite direction. This establishes that the fluctuations are significant, and the opposite sign of their contribution suggests that they lead to additional order in the system.

Several aspects of the formalism and calculations presented here have been performed in such a way as to be accessible to students and others wishing to extend these techniques to more realistic models of polyelectrolytes condensing on the surfaces of DNA. We have shown in Section 3 why the DNA helices can be treated as a pair of one-dimensional lattices (Figure 9), albeit with unusual interactions due to the three-dimensional nature of the screened Coulomb potential, as shown in Figure 12. We show that by writing the interaction term in the diagonal basis, as in Equation (71) and Figure 14, the problem can be greatly simplified. In addition, we have chosen a basis in which the eigenvalues and eigenvectors are real, in Equations (74) and (75), which makes the physical interpretation much more transparent. Then, we show in detail in Equation (87) and Appendix A how to perform a Hubbard–Stratonovich transformation of the partition function to decouple the sum over configurations from the sum over lattice sites, which allows one to perform the sum over all configurations of the system exactly. This introduces an integral over an auxiliary field $\tilde{\Delta }$. Often, such an auxiliary field emerges as a complicated complex matrix, but in our diagonal representation it is real and represents the charge per site in the diagonal basis, which was shown explicitly for the mean field by comparing Equations (123) and (130). Also, in this diagonal basis, it becomes clear how to choose a proper integration path to have a converging integral. One must then expand the effective action in a power series in the fluctuations of the auxiliary field $\delta \tilde{\Delta }$, as in Equation (104), which physically is in powers of the fluctuations of the charge per site about the mean field. Setting the first-derivative term in this expansion to zero yields the mean field, which itself includes terms of the interaction to all orders, as can be seen in Equation (122) or (123). Whether the expansion is useful depends on the size of the charge fluctuations. The series is an asymptotic one, which means that the infinite series does not necessarily converge. However, as is true for all asymptotic expansions, it is useful as long as the last term kept in the expansion is smaller than the term before it, which is true in our case. This indicates that the mean field, which is the saddle point, is a reasonable first approximation of the system. The fluctuations about this mean field then give insight into how the actual solution differs from the mean field.

In conclusion, we have shown with this simple model the importance of including fluctuation contributions beyond the mean field. The method we have used has allowed us to include vacancies, and we have found that the inclusion of the fluctuation term decreases the calculated entropy by ∼50% in the weak-binding regime. This is because, due to the presence of vacancies, the bound dimer concentration is low because of the competition between dimers being repelled from the DNA molecule and the chemical potential driving them from the solution onto the DNA surface. It is surprising that this decrease in entropy due to correlations is so significant that it overcompensates for the entropy increase at the mean-field level, so that the total entropy is even lower than in the absence of interactions between lattice sites. This effect of fluctuations could be important for other biological systems as well, and this approach can be useful where correlations are significant.

## Figures and Tables

**Figure A1:**
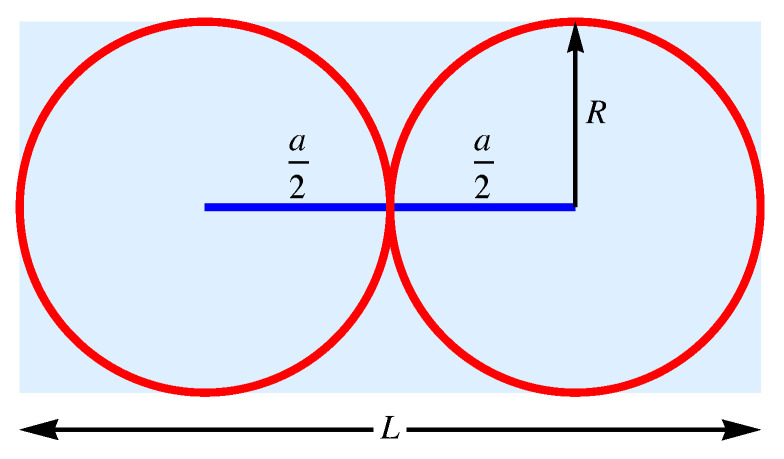
A free dimer approximated by a cylinder, shown in cross-section, where the dimer is designed to attach to two phosphates separated by lattice spacing *a*. The length of the cylinder is L=2a and the radius is $R=\frac{a}{2}$, making the ratio $\xi =\frac{L}{R}=4$.

## Data Availability

Not applicable.

## Appendix A. A Gaussian Integral Identity

In this appendix, we prove the integral identity that we use in the derivation of the partition function $Z_{G}$ for the interacting model. The identity is

(A1) $$I=\int \sqrt{\frac{\beta \lambda }{-2\pi }}d\tilde{\Delta }\;e^{\frac{\beta }{2}\lambda {\tilde{\Delta }}^{2}-\beta \lambda \tilde{\Delta }\tilde{\rho }}e^{\frac{\beta }{2}\lambda {\tilde{\rho }}^{2}}=1\;.$$

The integration path is over the real axis from −∞ to *∞* when λ<0, and over the imaginary axis from −i∞ to i∞ when λ>0. This integral can be evaluated by noticing that the exponent is a factor times a perfect square,

(A2) $$\frac{\beta }{2}\lambda {\tilde{\Delta }}^{2}-\beta \lambda \tilde{\Delta }\tilde{\rho }+\frac{\beta }{2}\lambda {\tilde{\rho }}^{2}=\frac{\beta }{2}\lambda {\left(\tilde{\Delta }-\tilde{\rho }\right)}^{2}\;.$$

Substituting, the integral becomes

(A3) $$I=\int \sqrt{\frac{\beta \lambda }{-2\pi }}d\tilde{\Delta }e^{\frac{\beta }{2}\lambda {\left(\tilde{\Delta }-\tilde{\rho }\right)}^{2}}\;.$$

Now it is clear why the path of integration must be chosen differently for positive versus negative λ, since it must be chosen such that the integral converges. If λ=0, the identity fails, but in that case it is clearly unnecessary. If λ<0, we can write λ=−|λ|, and the integral converges for $\tilde{\Delta }$ ranging over the real axis from −∞ to *∞*. Substituting $y=\tilde{\Delta }-\tilde{\rho }$ with *y* ranging from −∞ to *∞*, the integral can be evaluated as

(A4) $$\begin{array}{ccc} I & = & \int_{-\infty }^{\infty }\sqrt{\frac{\beta |\lambda |}{2\pi }}dy\;e^{-\frac{\beta }{2}\left|\lambda \right|y^{2}}=\sqrt{\frac{\beta |\lambda |}{2\pi }}\sqrt{\frac{2\pi }{\beta |\lambda |}}\\ & = & 1\;,\;\;(\lambda <0)\;. \end{array}$$

For λ>0, the situation is slightly more complicated. In that case, in order to have a decaying Gaussian in the integrand, we must integrate $\tilde{\Delta }$ over the imaginary axis from −i∞ to i∞. This is accomplished by substituting $y=-i\left(\tilde{\Delta }-\tilde{\rho }\right)$ for *y* ranging from −∞ to *∞*. In this case, we have

(A5) $$\begin{array}{ccc} I & = & i\int_{-\infty }^{\infty }\sqrt{\frac{\beta \lambda }{-2\pi }}dy\;e^{-\frac{\beta }{2}\lambda y^{2}}=i\sqrt{\frac{\beta \lambda }{-2\pi }}\sqrt{\frac{2\pi }{\beta \lambda }}\\ & = & 1, \end{array}$$

where the *i* in the numerator cancels the $\sqrt{-1}$ in the denominator. This proves the identity for positive λ. Therefore, we have seen that this identity works for positive and negative values of λ, and, although it does not work for λ=0, it is not needed in that case.

## Appendix B. Chemical Potential of Free Dimers

The chemical potential of a solution of free dimers is the derivative of the Helmholtz free energy of the solution of free dimers $F_{\mathrm{dimer}}$ with respect to the number of dimers $N_{\mathrm{dimer}}$, with temperature and volume held constant,

(A6) $$\mu_{\mathrm{dimer}}={\left(\frac{\partial F_{\mathrm{dimer}}}{\partial N_{\mathrm{dimer}}}\right)}_{T,V}\;.$$

Assuming that $F_{\mathrm{dimer}}$ is due only to electrostatic interactions, in a dilute solution the free energy is just the number of dimers times the electrostatic self-energy $E_{\mathrm{dimer}}$ of a single dimer. Then, the chemical potential becomes

(A7) $$\mu_{\mathrm{dimer}}={\left(\frac{\partial (N_{\mathrm{dimer}}E_{\mathrm{dimer}})}{\partial N_{\mathrm{dimer}}}\right)}_{T,V}=E_{\mathrm{dimer}}\;,$$

and so we need the electrostatic energy of a single dimer. Suppose we model a dimer as a cylinder of cross-sectional radius *R* and length *L* of total charge *Q*, as shown in Figure A1. An exact solution to this problem has been provided by Cifta [56], and the derivation proceeds as follows. The volume charge density of the cylinder is given by

(A8) $$\rho_{0}=\frac{Q}{\pi R^{2}L}\;,$$

and the Coulomb self-energy is given by

(A9) $$E_{s}=\frac{1}{4\pi \epsilon }\left(\frac{\rho_{0}^{2}}{2}\right)\int_{V}d^{3}r_{1}\int_{V}d^{3}r_{2}\frac{1}{|{\vec{r}}_{1}-{\vec{r}}_{2}|}\;,$$

where ϵ is the dielectric permeability of the medium surrounding the cylinder. In evaluating the integrals, the length to radius ratio is defined as

(A10) $$\xi =\frac{L}{R}\;,$$

and the exact result, as calculated by Cifta [56], is given by

(A11) $$E_{s}\left(\xi \right)=\frac{1}{4\pi \epsilon }\left(\frac{4Q^{2}}{R\xi^{2}}\right)\left\{\frac{\xi }{16}\left[4\ln\left(2\xi \right)-3\right]+\frac{32}{45\pi }-\left(\frac{1}{16\xi }\right){}_{4}F_{3}\left(\frac{1}{2},1,1,\frac{5}{2};2,3,4;-\frac{4}{\xi^{2}}\right)\right\}\;,$$

where the function ${}_{p}F_{q}\left(a_{1},a_{2},\ldots ,a_{p};b_{1},b_{2},\ldots ,b_{p};z\right)$ is the generalized hypergeometric function whose series expansion is

(A12) $${}_{p}F_{q}\left(a_{1},a_{2},\ldots ,a_{p};b_{1},b_{2},\ldots ,b_{p};z\right)=\sum_{n=0}^{\infty }\frac{{\left(a_{1}\right)}_{n}{\left(a_{2}\right)}_{n}\ldots {\left(a_{p}\right)}_{n}}{{\left(b_{1}\right)}_{n}{\left(b_{2}\right)}_{n}\ldots {\left(b_{q}\right)}_{n}}\frac{z^{n}}{n!}\;,$$

where the variable *z* can be either real or complex and ${\left(c\right)}_{n}$ is the Pochhammer symbol defined as

(A13) $${\left(c\right)}_{n}=\frac{\Gamma (c+n)}{\Gamma (c)}\;,\;n=1,2,\ldots \;\;.$$

Here, Γ(z) is the gamma function and ${\left(c\right)}_{0}=1$. For a long thin rod, the logarithm term would dominate, but a dimer approximated by a cylinder, pictured in Figure A1, does not fall into this regime.

As shown in the picture, for the dimer, the radius of the dimer is $R=\frac{a}{2}$, where *a* is the lattice spacing along a DNA helical chain. The length of the dimer is L=2a, and so the length ratio is $\xi =\frac{L}{R}=4$. The charge is Q=2e, where *e* is the magnitude of the charge of an electron, and the chemical potential becomes

(A14) $$\begin{array}{ccc} \mu_{\mathrm{dimer}} & = & E_{\mathrm{dimer}}=\\ & = & \frac{1}{4\pi \epsilon }\left(\frac{e^{2}}{8a}\right)\left\{\frac{1}{4}\left[4\ln\left(8\right)-3\right]+\frac{32}{45\pi }-\left(\frac{1}{128}\right){}_{4}F_{3}\left(\frac{1}{2},1,1,\frac{5}{2};2,3,4;-\frac{1}{4}\right)\right\}\;. \end{array}$$

For the parameters given in the text, the chemical potential for the dimer, in units of $\beta =\frac{1}{k_{B}T}$ at T=300 K, is given by $\beta \mu_{\mathrm{dimer}}=0.79$. It is interesting to note that approximately the same value was obtained by Nguyen and Shklovskii [27] and Bishop and McMullen [28] assuming that the dimer is an infinitely thin rod of length *L* but that there were upper and lower screening cutoffs. This exact solution avoids the use of those cutoffs.
